# Design, Synthesis, and Molecular Modeling Studies of a Novel Benzimidazole as an Aromatase Inhibitor

**DOI:** 10.1021/acsomega.2c01497

**Published:** 2022-04-28

**Authors:** Ulviye Acar Çevik, Ismail Celik, Jaime Mella, Marco Mellado, Yusuf Özkay, Zafer Asım Kaplancıklı

**Affiliations:** † Department of Pharmaceutical Chemistry, Faculty of Pharmacy, Anadolu University, Eskişehir 26470, Turkey; ‡ Department of Pharmaceutical Chemistry, Faculty of Pharmacy, Erciyes University, Kayseri 38039, Turkey; § Institute of Chemistry and Biochemistry, Faculty of Sciences, University of Valparaíso, Av. Great Britain, 1111 Valparaíso, Chile; ∥ Institute of Chemistry, Faculty of Sciences, Pontificia Universidad Católica de Valparaíso. Av. Universidad 330, Curauma, 0000 Valparaíso, Chile

## Abstract

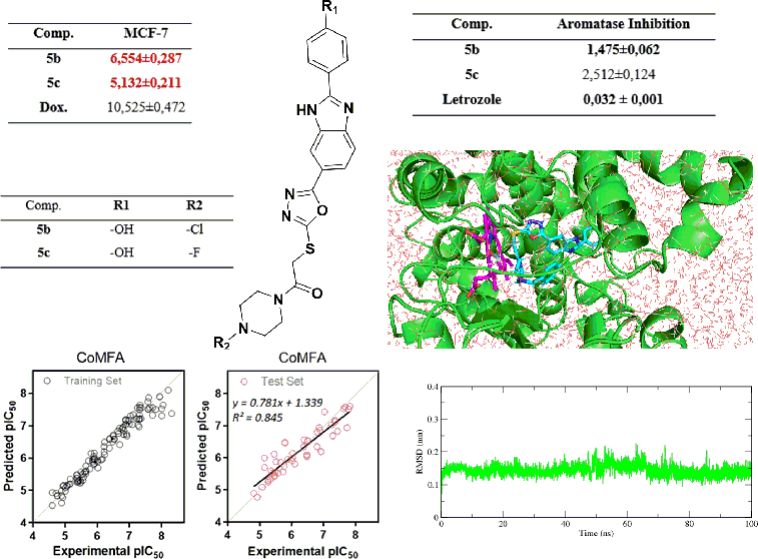

In this study, a series of novel 1,3,4-oxadiazole-benzimidazole derivatives were designed and synthesized. Their cytotoxic activities against five cancer cell lines, including A549, MCF-7, C6, HepG2, and HeLa, were evaluated by the MTT assay. The compounds **5b**,**c** showed satisfactory potencies with much higher anticancer activity in comparison to the reference drug doxorubicin against the studied cancer cell lines. *In vitro*, enzymatic inhibition assays of aromatase (ARO) enzymes were performed. Molecular docking, molecular dynamics simulations, and binding free energy analyses were used to better understand the structure–activity connections and mechanism of action of the aromatase inhibitors. Two types of satisfactory 3D-QSAR (CoMFA and CoMSIA) models were generated, to predict the inhibitory activities of the novel inhibitors. Molecular docking studies were also carried out to find their binding sites and types of their interactions with the aromatase enzyme. Additionally, molecular dynamics simulations were performed to explore the most likely binding modes of compounds **5b**,**c** with CYP19A1.

## 1 Introduction

Cancer is a multifactorial complex disorder, characterized by the uncontrolled growth and spread of abnormal cells.^1^ On the basis of a WHO survey, cancer has been responsible for almost 9.6 million deaths worldwide in 2018 and there will be around 13.1 million estimated new cases by 2030.^2,3^ After cardiovascular disease, cancer is the second leading cause of death.^4^ Although there are many drugs for the treatment of cancer, the need for continued research for new anticancer molecules remains due to selectivity, potency, and drug resistance.^5^

Breast tissue has an excess of the aromatase enzyme.^6^ Aromatase inhibitors (AIs) are a well-known endocrine therapy method that works by suppressing estrogen production, which is important for aromatase activity.^7^ AIs are divided into four generations based on their clinical development order: first, second, third, and fourth generation AIs. AIs are divided into two types: type I (steroidal) and type II (nonsteroidal) on the basis of their mechanism of action.^8^ The most effective, selective, and least toxic aromatase inhibitors are the third-generation aromatase inhibitors (anastrozole and letrozole). In actual practice, third-generation inhibitors provide a better therapeutic benefit with practically full specificity. Letrozole and anastrozole are nonsteroidal derivatives that have a triazole ring which interacts with the heme prosthetic group of aromatase and competes with androgen substrates by inhibiting them competitively.^9^

A benzimidazole ring, which is an important core in medicinal chemistry, constitutes structures found in current drugs. The ring structure is also an isostere of DNA bases that carry purine and pyrimidine cores, and it can as well be a purine antimetabolite.^5^ It has been proven by various studies that benzimidazole derivatives with anticancer activity show their activities by different mechanisms through DNA intercalation, topoisomerase I and II inhibition, an androgen receptor antagonistic effect, PARP-poly inhibition, dihydro-folate reductase (DHFR), aromatase inhibition, and microtubule inhibition.^10−15^ Various classes of therapeutically active benzimidazole derivatives are available in the market such as bendamustine, veliparp, carbendazim, and nocodazole.

In our previous studies, we synthesized a series of benzimidazole-oxadiazole derivatives which showed satisfactory anticancer activity with good IC_50_ values against various cell lines.^16,17^ As part of a continuing effort to find out and screen more active benzimidazole-oxadiazole derivatives, in the present study, a series of novel 4-phenyl- and 4-piperazinyl-substituted 2-{[5-(2-phenyl-1*H*-benzo[*d*]imidazol-6-yl)-1,3,4-oxadiazol-2-yl]thio}-1-piperazin-1-ylethan-1-one derivatives were synthesized which were later confirmed by ^1^H NMR, ^13^C NMR, and mass spectral techniques. Then, their cytotoxic activities against various cancer cell lines including A549 (human lung adenocarcinoma epithelial cell line), HepG2 (human colon cancer cell line), MCF-7 (breast adenocarcinoma), C6 (breast adenocarcinoma), and HeLa (ovarian cancer cell line) were evaluated. Using the crystal structure of aromatase retrieved from the Protein Data Bank (PDB ID 3EQM) and Autodock Vina software, docking experiments corroborated the results obtained from biological screening by predicting probable binding interactions of the target compounds with aromatase active sites. Molecular dynamic simulations were applied to evaluate the stability in the aromatase active site to justify their inhibitory activity.

## 2 Results and Discussion

### 2.1 Chemistry

**Scheme 1:**
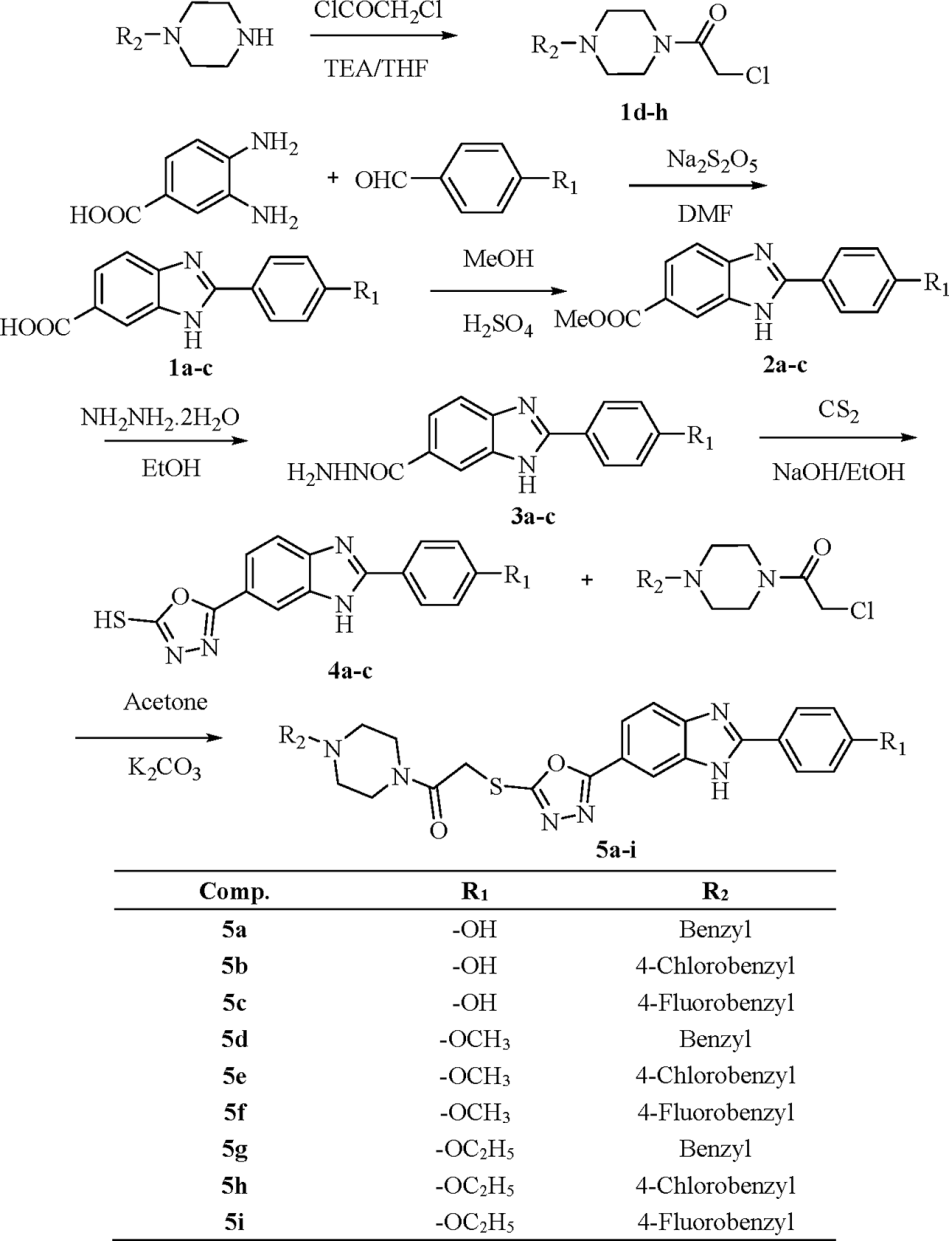
Synthesis of the Compounds **5a**–**i**

Scheme 1 depicts the synthesis of the target compounds **5a**–**i**. In the first stage, a 4-substituted benzaldehyde was heated with 3,4-diaminobenzoic acid in DMF and sodium metabisulfite to obtain the derivatives **1a**–**c**. By using a Fischer esterification procedure, compounds **1a**–**c** were transformed into the methyl ester compounds **2a**–**c.** To prepare compounds **3a**–**c**, appropriate solutions of compounds **2a**–**c** in ethanol (95%) were treated with hydrazine hydrate. Compounds **4a**–**c** were formed by reacting hydrazide derivatives **3a**–**c** with carbon disulfide in ethanolic potassium hydroxide. To make the target compounds **5a**–**i**, compounds **4a**–**c** were reacted with acetylated piperazine derivatives in acetone in the final reaction step. Using current analytical techniques such as ^1^H NMR, ^13^C NMR, and HRMS, the structures of synthesized compounds **5a**–**i** were studied.

The S–CH_2_ (methylene) protons were detected as a singlet between 4.53 and 4.63 ppm, according to a ^1^H NMR spectral analysis of compounds **5a**–**i**. At 3.04–3.86 ppm, the protons of the piperazine moiety appear as a multiplet. The protons of the methoxy substituent provided a singlet signal at 3.84–3.86 ppm in the ^1^H NMR spectra of compounds **5d**–**f** with a 4-methoxyphenyl group in the second position of the benzimidazole ring. The −OCH_2_ protons were found at 4.02–4.11 ppm in the −OC_2_H_5_ group of compounds **5g**–**I** on the phenyl ring, and CH_3_ protons were found at 1.24–1.35 ppm. Due to the H-5 proton, the benzimidazole proton was observed as a doublet of doublets in ^1^H NMR spectra at around 7.68–7.87. The carbon atoms of the molecule have chemical shift values that are similar to those reported in the literature in ^13^C NMR. The masses of the target compounds were validated by an HRMS analysis, which confirmed the estimated values.

### 2.2 Cytotoxicity Assay

In this study, all compounds were tested for antiproliferative activity using an MTT assay with doxorubicin as a control against MCF-7 (human breast adenocarcinoma cell line), A549 (lung carcinoma cell line), HepG2 (human liver carcinoma cell line), HeLa (cervical cell line), and C6 (rat glioma cell line) cell lines. Table 1 summarizes the results of four independent studies as the mean IC_50_ value (half-maximum inhibitory concentration).

**Table 1:** IC_50_ Values (μM) of the Compounds **5a**–**i** and Doxorubicin against A549, MCF-7, C6, HepG2, and HeLa

compd	A549	MCF-7	C6	HepG2	HeLa
**5a**	≥100	**9.056 ± 0.311**	≥100	38.778 ± 0.887	≥100
**5b**	≥100	**6.554 ± 0.287**	24.675 ± 1.213	55.231 ± 1.456	≥100
**5c**	≥100	**5.132 ± 0.211**	18.214 ± 1.459	17.331 ± 1.099	≥100
**5d**	≥100	≥100	88.512 ± 3.412	≥100	≥100
**5e**	≥100	45.643 ± 2.11	≥100	≥100	≥100
**5f**	≥100	≥100	≥100	≥100	≥100
**5g**	≥100	≥100	≥100	≥100	≥100
**5h**	≥100	≥100	≥100	≥100	≥100
**5i**	**4.523 ± 0.210**	**9.963 ± 0.312**	**5.995 ± 0.227**	28.662 ± 1.224	42.042 ± 1.317
**Dox.**	12.420 ± 0.521	10.525 ± 0.472	28.690 ± 1.228	16.482 ± 0.804	14.280 ± 0.704

The benzimidazole derivatives **5b**,**c** possessing 4-chlorobenzyl and 4-fluorobenzyl groups were the most potent compounds against the MCF-7 (IC_50_= 5.132 ± 0.211, 6.554 ± 0.287 μM) cell line, respectively. Furthermore, compounds **5a**,**I** exhibited moderate activity against MCF7. Compound **5c** was the most active agent against the HeLa cell line (IC_50_ = 7.316 ± 0.276 μM). Furthermore, compound **5i** exhibited the best activity against A549 and C6 cell lines. In addition, among the compounds **5a**–**i**, compounds **5a**,**i** exhibited anticancer activity similar to that of doxorubicin against MCF7 cell line with IC_50_ values of 9.963 ± 0.312 and 9.056 ± 0.311 μM, respectively.

According to the findings, the 4-hydroxyphenyl group boosted anticancer activity in MCF-7, C6, and HepG-2 cancer cell lines more than the 4-methoxyphenyl and 4-ethoxyphenyl groups. The presence of chlorobenzyl and fluorobenzyl groups at the fourth position of the piperazine scaffold also increased anticancer activity, in comparison to 4-chlorophenyl and benzyl substituents.

### 2.3 Aromatase Inhibition Assay

An *in vitro* fluorescence-based test (Aromatase (CYP19A) Inhibitor Screening Kit (Fluorimetric), BioVision) was used to determine the inhibitory activity of the produced compounds against human aromatase. The target drug was dissolved in acetonitrile, and the results were compared to those for the reference compound, letrozole. According to the result, compounds **5b**,**c** effectively caused 50% aromatase enzyme inhibition at 1.475 ± 0.062 and 2.512 ± 0.124 μM, respectively (Table 2).

**Table 2:** IC_50_ (μM) Values of Compounds

compd	aromatase inhibition
**5a**	38.612 ± 1.129
**5b**	**1.475 ± 0.062**
**5c**	2.512 ± 0.124
**5i**	80.375 ± 3.125
letrozole	**0.032 ± 0.001**

### 2.4 Molecular Docking Analysis

**Figure 1:**
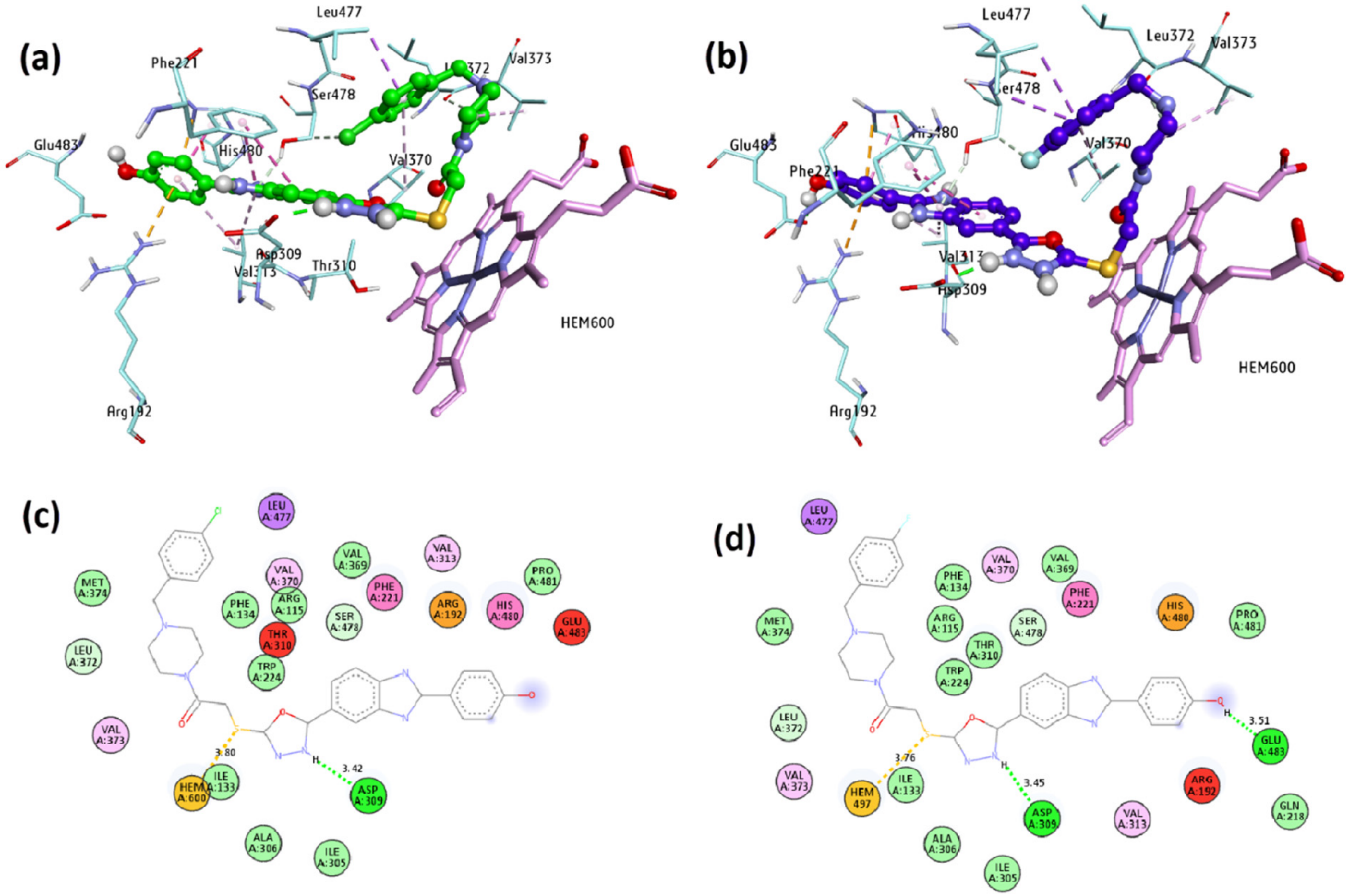
Autodock Vina molecular docking results of compounds **5b**,**c** against human placental aromatase cytochrome P450 protein (CYP19A1): (a) binding poses of **5b** (green) and (b) **5c** (blue) in the CYP19A1 active site together with Hem (magenta); schematic 2D protein–ligand interactions of (**c**) CYP19A1–**5b** and (d) CYP19A1–**5c** complexes.

A molecular docking analysis of **5b**,**c**, two of the most active compounds designed and synthesized in this study, was performed. For the molecular docking study, the 3D crystal structure of the aromatase enzyme was downloaded from the Protein Data Bank (PDB ID 3EQM, resolution 2.90 Å). Heteroatoms in the structure, except for the HEM structure, were removed. The active site amino acids of the protein were determined on the basis of the cocrystal 4-androstene-3,17-dione (ASD) natural ligand. Molecular docking validation was achieved by removing the ASD ligand found in the 3EQM crystal structure and redocking the same area. Between the cocrystal ligand and the binding mode obtained by docking, the RMSD was measured to be 0.46 Å. Ligand structures were minimized using a universal force field (UFF). As shown in Figure 1, compounds **5b**,**c** gave almost the same geometry binding pose at the CYP19A1 active site. Compound **5b** formed a conventional H bond and a Hem600 hydrophobic π-sulfur interaction between the NH of oxazole and Asp309 at the CYP19A1 active site. Other interactions of compound **5b** are given in Table 3. Compound **5c**, on the other hand, formed Glu493 with phenol −OH, conventional H bonds with oxazole NH and Asp309, and a hydrophobic π–sulfur interaction with Hem600. Electrostatic and hydrophobic interactions between CYP19A1 and **5c** are detailed in Table 3. Also, the interactions and binding poses of compounds **5b**,**c** and the reference compound letrozole used in the experimental study at the CYP19A1 active site were compared. Compounds **5b**,**c** formed hydrophobic π–Sulfur interactions with the Hem600 structure. However, letrozole formed π–π T-shaped hydrophobic and π-donor hydrogen bond interactions with the Hem600 structure. Compounds **5a**,**b** and and letrozole formed hydrophobic interactions with Phe221. Protein–ligand interaction details of letrozole with the CYP19A1 enzyme are given in Table 3.

**Table 3:** Molecular Protein–Ligand Interaction Details of Compounds **5b**,**c** and Letrozole against Human Placental Aromatase Cytochrome P450 Protein (CYP19A1)

enzyme–compd	interacting residue	distance (Å)	category	type
CYP19A1–**5b**	Hem600	3.80	hydrophobic	π-sulfur
	Asp309	1.97	hydrogen bond	conventional hydrogen bond
	Ser478	3.53	hydrogen bond	carbon–hydrogen bond
	Leu372	3.42	hydrogen bond	carbon–hydrogen bond
	Arg192	3.53	electrostatic	π–cation
	His480	3.83	electrostatic	π–cation
	Ser478	2.46	hydrogen bond	π-donor hydrogen bond
	Leu477	3.43	hydrophobic	π–σ
	Phe221	4.24	hydrophobic	π–π stacked
	His480	3.53	Hydrophobic	π–π stacked
	Phe221	3.69	hydrophobic	π–π stacked
	Val373	5.07	hydrophobic	alkyl
	Val313	5.09	hydrophobic	π–alkyl
	Val313	5.03	hydrophobic	π–alkyl
	Val370	5.43	hydrophobic	π–alkyl
				
CYP19A1–**5c**	Hem600	3.76	hydrophobic	π–sulfur
	Glu483	1.45	hydrogen bond	conventional hydrogen bond
	Asp309	1.94	hydrogen bond	conventional hydrogen bond
	Ser478	3.40	hydrogen bond	carbon–hydrogen bond
	Leu372	3.42	hydrogen bond	carbon–hydrogen bond
	Arg192	3.51	electrostatic	π–cation
	His480	3.84	electrostatic	π–cation
	Ser478	2.47	hydrogen bond	π-donor hydrogen bond
	Leu477	3.89	hydrophobic	π–σ
	Leu477	3.62	hydrophobic	π–σ
	Phe221	4.21	hydrophobic	π–π stacked
	His480	3.54	hydrophobic	π–π stacked
	Phe221	3.67	hydrophobic	π–π stacked
	Val373	5.23	hydrophobic	alkyl
	Val313	5.10	hydrophobic	π–alkyl
	Val313	5.02	hydrophobic	π–alkyl
	Val370	5.17	hydrophobic	π–alkyl
				
CYP19A1–letrozole	Hem600	3.00	hydrogen bond	π-donor hydrogen bond
	Hem600	5.24	hydrophobic	π–π T-shaped
	Met374	1.88	hydrogen bond	conventional hydrogen bond
	Arg115	2.84	hydrogen bond	conventional hydrogen bond
	Thr310	3.13	hydrophobic	π-σ
	Phe221	4.88	hydrophobic	π–π stacked

### 2.5 Molecular Dynamics Simulations

**Figure 2:**
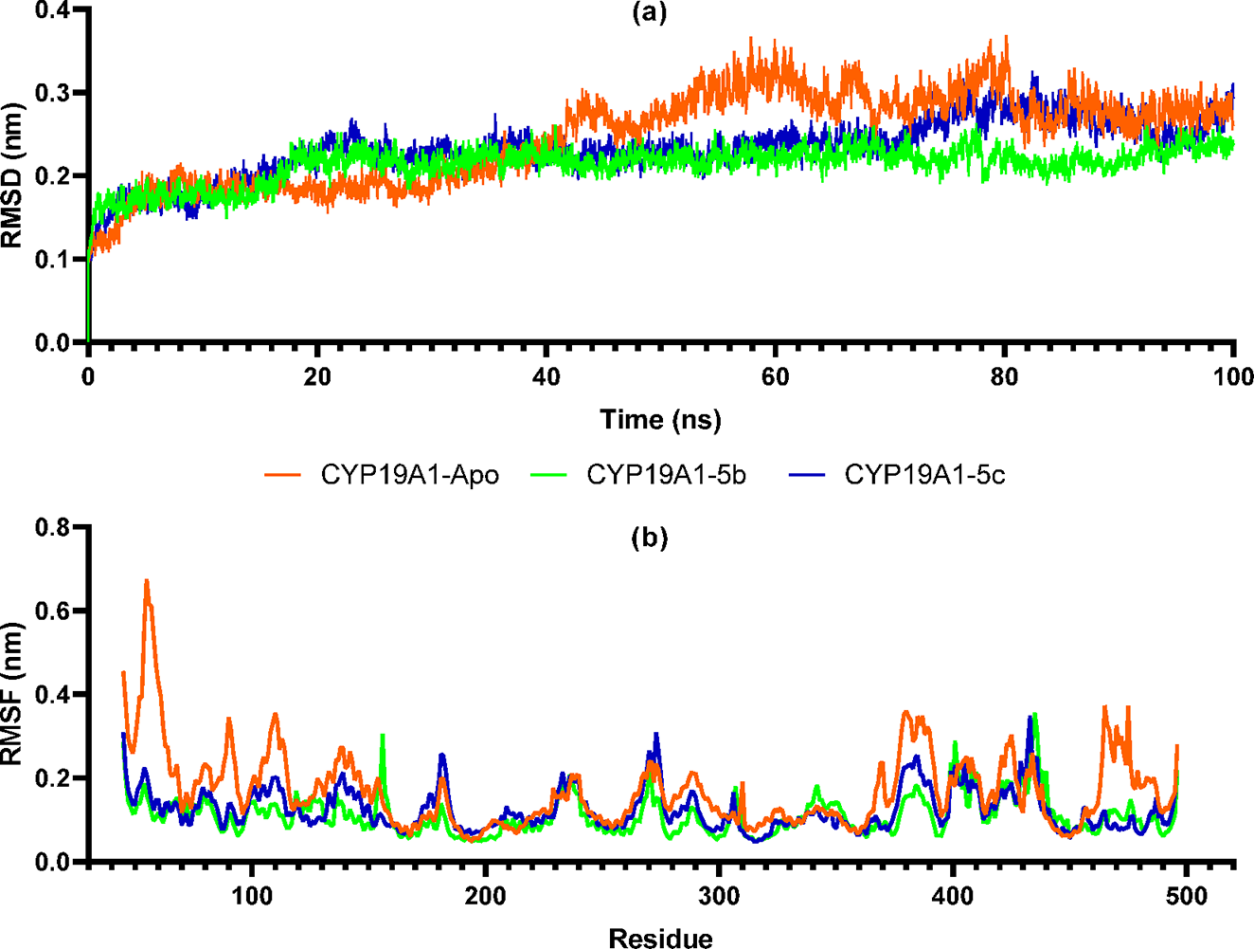
Trajectory analysis of molecular dynamics simulations of human placental aromatase cytochrome P450 protein (CYP19A1), apoprotein (CYP19A1–Apo), and compound **5b** (CYP19A1–**5b**) and compound **5c** (CYP19A1–**5c**) complexes: (a) RMSD of CYP19A1–**5b** and CYP19A1–**5c** complexes and CYP19A1–Apo apoprotein; (b) RMS fluctuation values during the period of 100 ns simulation.

Molecular dynamics (MD) simulations are frequently used to detect the *in silico* variation of protein–ligand interactions. The stability of the protein–ligand complex structure obtained from the molecular docking study can be estimated by calculating with an MD simulation. In this study, the protein–ligand complexes obtained from the molecular docking study of compounds **5b**,**c** against the aromatase enzyme and the apoprotein without ligand were run under the same conditions for 100 ns to provide an MD validation. Topology files of the **5b**,**c** ligands, the CYP19A1 enzyme, and the HEM structure it carries were obtained using the charmm36-feb2021 force field. RMSD and RMSF trajectory analyzes were performed. RMSD is a parameter that is frequently used to examine deviations and changes in protein structure. The RMSD measurement was measured by preferring the backbone atoms of the protein. CYP19A1 apoprotein, CYP19A1–**5b**, and CYP19A1–**5c** protein–ligand complex structures were measured as the means 0.32, 0.23, and 0.27 nm, respectively. As seen in Figure 2, the protein became more stable because of the interaction of **5b**,**c** with CYP19A1. In particular, the CYP19A1–**5b** complex was stabilized below 0.2 nm. The other analysis parameter, RMSF, shows fluctuations and conformational changes in the protein’s structure. A lower RMSF value indicates that the protein is more stable. As shown in Figure 2, residues at the N- and C-termini of the CYP19A1 apoprotein fluctuated more than the case for the CYP19A1–**5b** and CYP19A1–**5c** complex. Compounds **5b**,**c** formed strong interactions with the aromatase enzyme, making CYP19A1 more stable.

**Figure 3:**
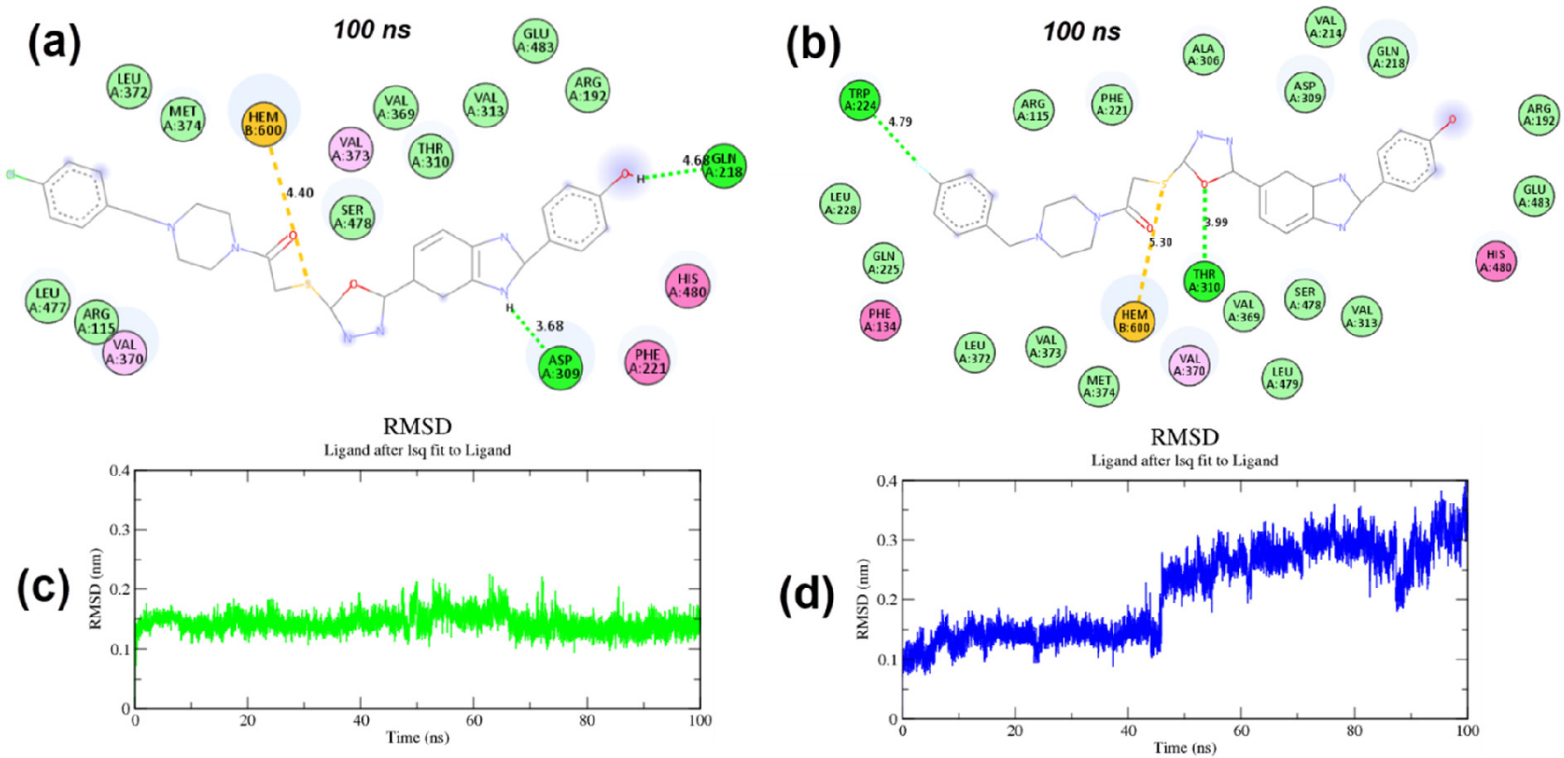
2D schematic protein–ligand interactions after 100 ns of (a) compound **5b** and (b) compound **5c** in the CYP19A1 active site. Ligand RMSD plots of (c) compound **5b** (average value 0.14 nm) and (d) compound **5c** (average value 0.21 nm) during 100 ns molecular dynamics simulations.

The 10000th frame protein–ligand interactions are presented in Figure 3 to explain the changes in the CYP19A1 active site at the end of 100 ns simulations of compounds **5b**,**c**. The π–sulfur interaction of Hem600 with the thioether group of **5b**,**c** was preserved. Compound **5b** formed Asp309 with the benzimidazole core −NH and Gln218 with the phenol −OH. In compound **5c**, H bonds were formed between the O atom of the oxazole ring and Thr310 and between fluorine and Trp224. As shown in Figure 3, **5b** was extremely stable (mean RMSD: 0.14 nm) during the MD simulation, while **5c** was stable up to about 40 nm, after which an increase to 0.4 nm (mean RMSD: 0.21 nm) was measured.

Another method to understand the stability of protein–ligand interactions in MD simulations is to measure protein–ligand interaction energies over time. The average short-range Coulombic interaction energy, the short-range Lennard–Jones energy, and MMPBSA for bindin -free energy (BFE) were measured for the protein–ligand interaction energy formed by compounds **5b**,**c** by the CYP19A1 enzyme. BFE was obtained from the sum of van der Waals, electrostatic, polar solvation, and SASA energies. As seen in Table 4 compounds **5b**,**c** formed strong interactions at the CYP19A1 enzyme active site with small deviations.

**Table 4:** Results of MM-PBSA Interaction Binding Free Energies, Average Short-Range Coulombic Interaction Energy, and the Short-Range Lennard–Jones Energy Calculation of CYP19A1-**5b** and CYP19A1-**5c** Complexes

	protein–ligand binding energy		
compd	MMPBSA binding free energy (kJ/mol)	Coulombic interaction energy (kJ/mol)	Lennard–Jones interaction energy (kJ/mol)
**5b**	–159.756 ± 16.25	–69.739 ± 5.1	–186.707 ± 5.1
**5c**	–176.875 ± 18.84	–67.712 ± 6.0	–201.912 ± 4.4

### 2.6 3D-QSAR Study

In order to complement the experimental analysis, we carried out a 3D-QSAR study using a comparative molecular field analysis (CoMFA) and a comparative molecular similarity index analysis (CoMSIA). The study was performed according to the previous report of our research group.^18−22^ Due to the scarce information on benzimidazole derivatives as aromatase inhibitors, we used the indole and benzofuran cores for the molecular alignment (all details are given in Table S1). All of the compounds span a biological activity range of more than 3 logarithmic units (Figure S2). The best model of each analysis (CoMFA and CoMSIA) was selected by a combination of the following feature combination: *q*^2^ > 0.5, a low number of components, and high value for the regression coefficient *r*^2^_ncv_. The models CoMFA-SE and CoMSIA-SH met these statistical criteria (Tables S2 and S3). A statistical summary of the best models (*q*^2^, *N*, SEP, SEE, *r*^2^_ncv_, *F*, PRESS, SD, and *r*^2^_pred_), is reported in Table S4.

**Figure 4:**
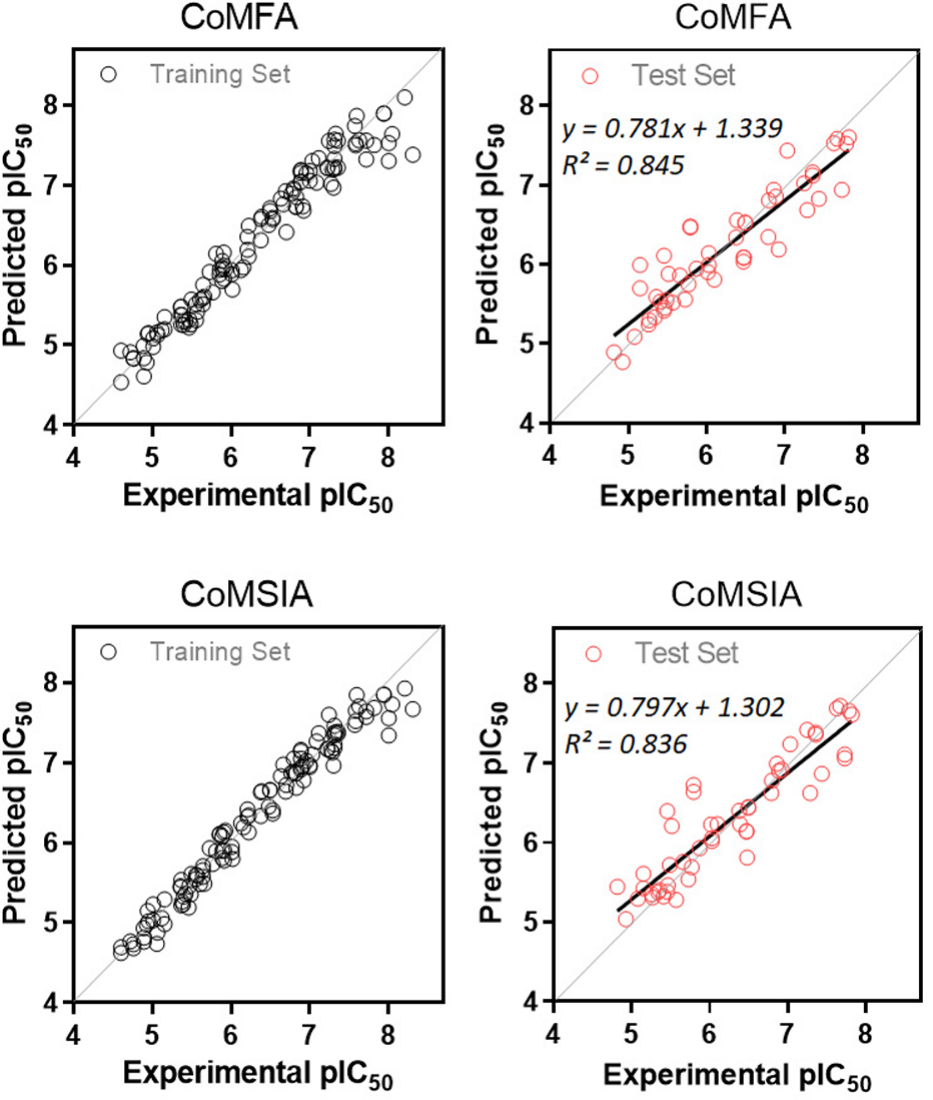
Plots of experimental values of aromatase inhibitors and predicted pIC_50_ values: (top left) CoMFA training set; (top right) CoMFA test set; (bottom left) CoMSIA training set; (bottom right) CoMSIA test set. For the test set, the regression line for *r*^2^ is shown as a boldface line.

The aromatase inhibition values, with the predictions made by the selected CoMFA-SE and CoMSIA-SH models, are presented in Table S5. The experimental versus predictive activity graphs for the best CoMFA and CoMSIA models were created to visualize whether there is an adequate linear distribution of the predictive results for both models (Figure 4). A good data distribution along the line *y = x* for both CoMFA and CoMSIA models for aromatase inhibitors in the training and test sets was observed. Moreover, each model was validated statistically using the Golbraikh and Tropsha methods.^23−26^ The CoMFA and CoMSIA models reported here fulfilled the main conditions for internal and external validation (Table S6). Finally, to avoid a random correlation, a *Y*-random test was executed, giving a low value of *q*^2^ (*q*^2^ = −0.225 to 0.012) and r^2^_ncv_ values below 0.5, confirming that both models developed are not a product of chance correlation (Table S7).

Finally, we performed the calculation of the applicability domain to corroborate the chemical suitability of all the compounds used in the studies. The applicability domain (AD) is a theoretical region in chemical space encompassing both the model descriptors and modeled response. It allows estimating the uncertainty in the prediction of a compound on the basis of how similar it is to the training compounds employed in the model. In this work, we used the method developed by Roy et al. for the determination of AD which is based on the basic theory of the standardization approach.^22^ In our cases, all the training and test set molecules, from both CoMFA and CoMSIA, were found within the applicability domain.

#### 2.6.1 Contour Map Analysis

**Figure 5:**
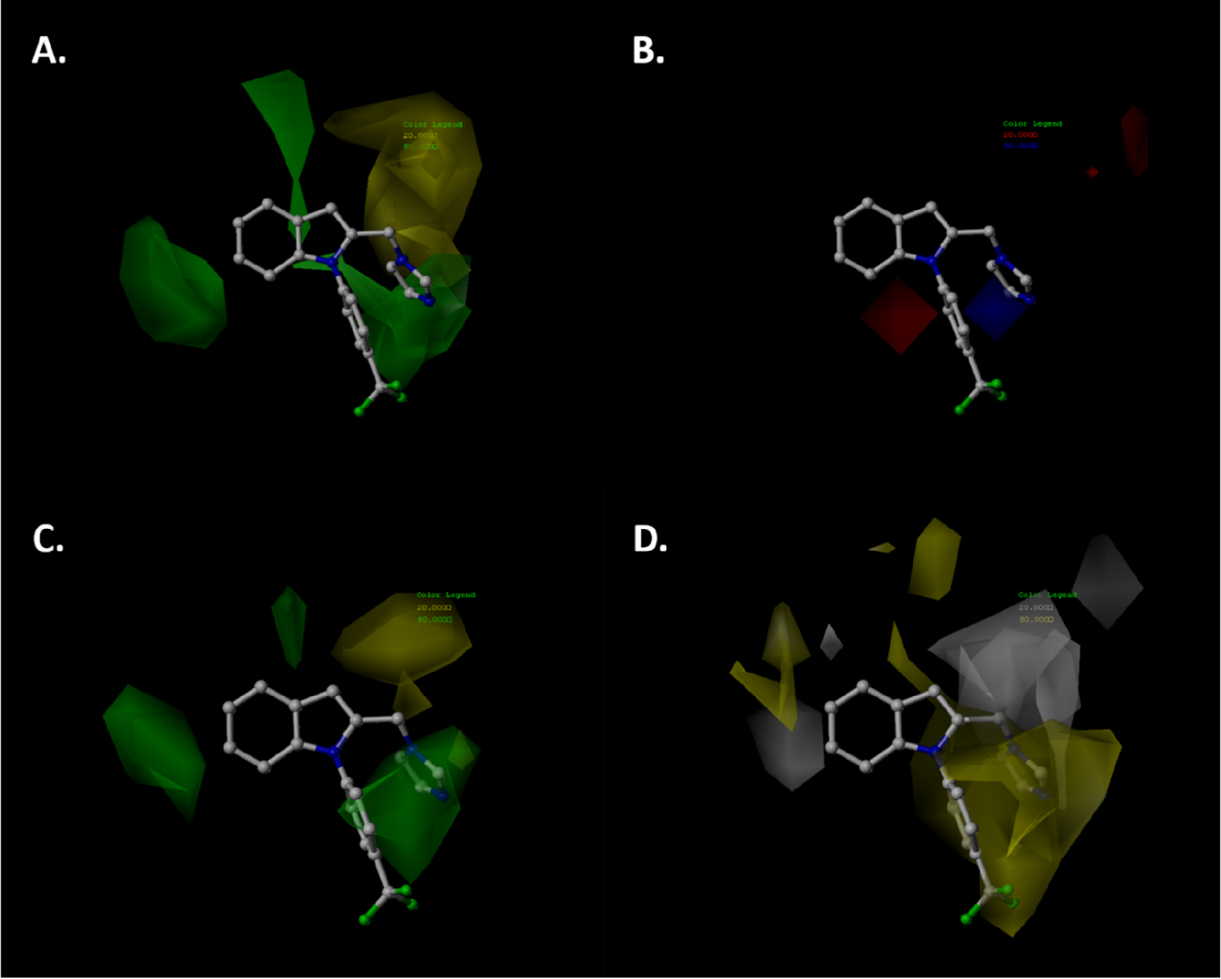
Contour maps of CoMFA and CoMSIA studies of aromatase inhibitors based on benzimidazole analogue pharmacophores: (A) steric map of the CoMFA study; (B) electrostatic map of the CoMFA study; (C) steric map of the CoMSIA study; (D) hydrophobic map of the CoMSIA study. Color code: green, bulky groups are favorable for activity; yellow, small groups are favorable for activity; red, negative charge is favorable for activity; blue, positive charge is favorable for activity.

The advantage of the use of the 3D-QSAR technique is obtaining contour maps around the target molecule. These contour maps show the main features as being favorable or unfavorable for the biological activity on the basis of the different colored polyhedra around a molecule. To visualize the contour maps, the compound 2-((1*H*-imidazol-1-yl)methyl)-1-(4-(trifluoromethyl)phenyl)-1*H*-indole (**13**) was used in the CoMFA-SE and CoMSIA-SH diagrams (Figure 5).

The CoMFA and CoMSIA steric maps of aromatase inhibitors are very similar (Figure 5A–C). In both figures, we can see a green polyhedron around position 6 of the indole core. This means that the bulk substituent favors the inhibitory activity. In fact, several indole derivatives with bulky substituents on position 6 have pIC_50_ > 6.0, for example, the compounds **11**–**28** (Me, OMe, or Cl substituents), as well as a similar substituent on position 6 of benzofuran core as in compounds **89**–**99**. Moreover, the substituent on position 5 of the indole core can project some fragments to near the green polyhedron, as for compound **63** with the phenyl group bonded to amino-sulfonyl. Interestingly, compounds **1**–**8** have a bulky substituent on chiral carbon that can project the *N*-imidazole or phenyl rings toward the green polyhedra. This phenomenon favors aromatase inhibition activity, concordant with pIC_50_ > 6.0. Moreover, a bulky substitution on the N-atom of the indole core improves the aromatase inhibition activity. This is due to the substituent being near the green polyhedron (Figure 5A–C), concordant with the high activity of derivatives **4**, **10**–**28**, **67**, **69**, **71**, **108**–**112**, and **117**–**121**. Also, between positions 2 and 3 of the indole core, there is a yellow polyhedron. This means that there is a decrease in aromatase inhibitory activity when a bulky substituent is bonded to these positions (e.g., compounds **29**–**45**, **47**–**51**, **53**–**62**, **64**–**66**, **68**, **70**, **72**, **104**–**107**, **113**–**116**, **122**, and **123**). All of these compounds showed an aromatase inhibitory activity pIC_50_< 6.0.

On the other hand, the CoMFA electrostatic map (Figure 5B) shows two main areas: the first between position 7 and the N-substituent of the indole core (red polyhedron) and the second between the N-substituent and position 2 of the indole core (blue polyhedron). In the first case, an electron-rich substituent improves the aromatase inhibitory activity as in compounds **108**, **109**, and **112**–**115** (pIC_50_ > 6.0). All of them have a halogen atom oriented toward the red polyhedron. In the second case, an electron-rich substituent causes a decrease in the aromatase inhibitory activity (pIC_50_< 6.0), as in compounds **47**–**51** and **53**–**62**. These compounds have a 2-indolin substituent bonded to position 2 of the indole core, oriented to the blue polyhedron.

The hydrophobic map of the CoMSIA model (Figure 5D) shows a white polyhedron near positions 5 and 6 of the indole core; this means that a hydrophobic substituent decreases the aromatase activity as in compounds **9**, **66**, **68**, **70**, **72**, **79**–**81**, **100**, **122**, and **123**, which have a methyl, halogen, or methoxy group and other substituents, as well as a value of pIC_50_ < 6.0. A similar effect is shown between positions 2 and 3 of the indole core, where a hydrophobic substituent causes a decrease in aromatase inhibitory activity as in compounds **29**–**45**, **56**–**62**, **64**–**66**, **68**, **70**, **72**, **100**–**110**, **113**–**116**, **122**, and **123**, which have analkyl or aromatic ring bonded to an alkyl group. Moreover, between the N-substituent and the group bonded to position 2 of the indole core, there is a yellow polyhedron, which means that the hydrophobic group causes an increased aromatase inhibition activity (pIC_50_ > 6.0). Some of the compounds with a hydrophobic group in this position are **11**–**28**, **67**, **69**, **71**, **73**–**78**, **89**–**99**, **108**–**112**, and **117**–**1**21 (e.g., *N*-alkyl, *N*-aryl, or an alkyl-aromatic substituent bonded to position 2 of indole core).

#### 2.6.2 Experimental Validation

To externally validate the models, we carried out the synthesis of four benzimidazole-type molecules. These compounds were predicted to have biological activity by the models reported here, and their experimental activity was determined by enzymatic assays. The results obtained are shown in Table 5.

**Table 5:** Experimental Validation of 3D-QSAR Model on Aromatase Inhibitors

compd	IC_50_ (μM)	exptl pIC_50_	CoMFA pIC_50_	residual	CoMSIA pIC_50_	residual
**5a**	3.86	4.413	6.066	–1.65	6.694	–2.28
						
**5b**	1.48	5.831	6.199	–0.37	6.490	–0.66
**5c**	2.51	5.600	6.268	–0.67	6.520	–0.92
**5i**	8.04	4.095	6.304	–2.21	6.539	–2.44

**Figure 6:**
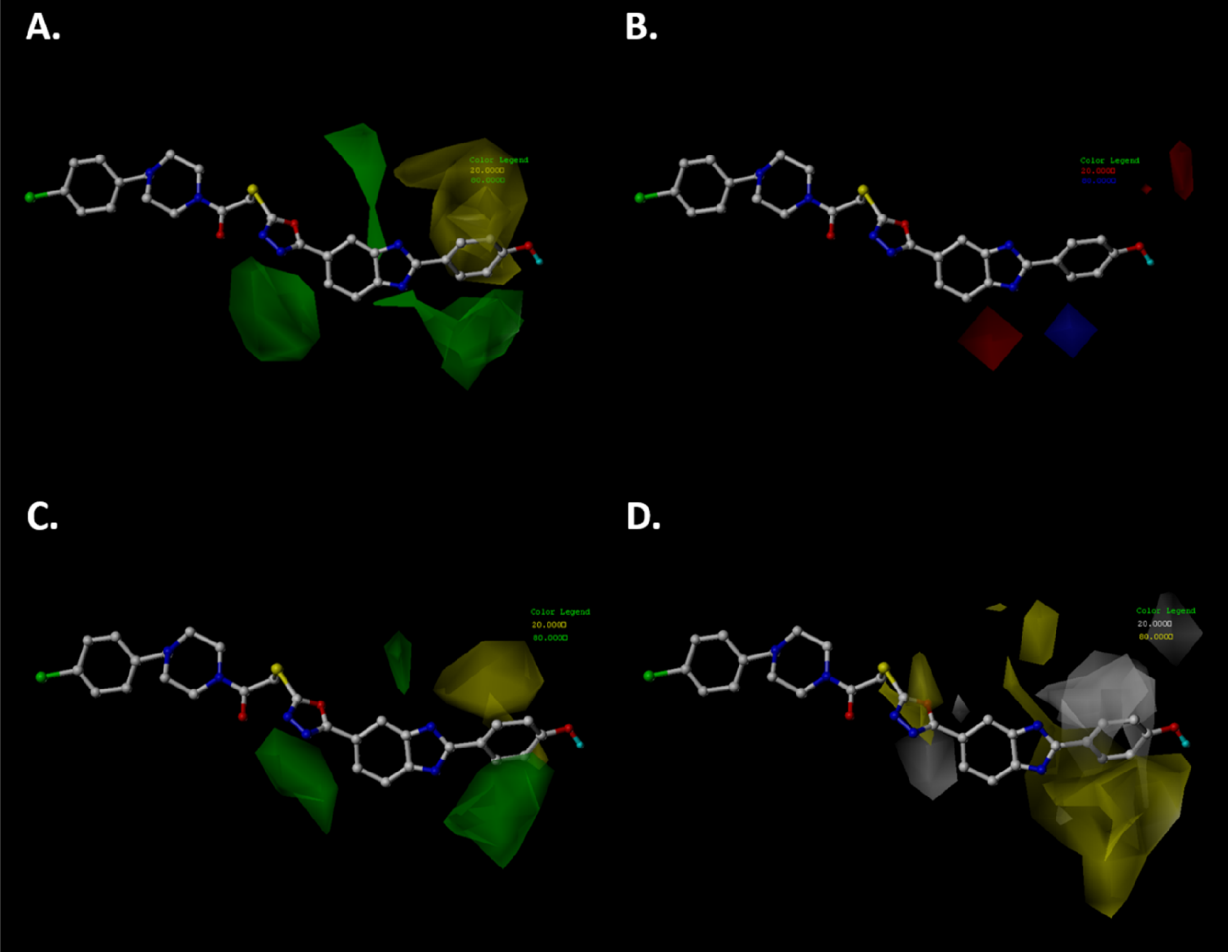
Compound **5b** overlaid on the CoMFA and CoMSIA contour map: (A) steric map of the CoMFA study; (B) electrostatic map of the CoMFA study; (C) steric map of the CoMSIA study; (D) hydrophobic map of the CoMSIA study. The color code is the same as that in Figure 5.

The predictive values based on CoMFA and CoMSIA models of aromatase inhibitors showed higher values in comparison to the experimental values. To explain this phenomenon, we overlaid compound **5b** on the contour map obtained from the CoMFA and CoMSIA study (Figure 6). In the steric map, the phenyl ring bonded to the benzimidazole moiety is oriented toward the yellow polyhedra, causing a decrease in the aromatase inhibition activity (Figure 6A–C). Moreover, the 1,3,4-oxadiazole fragment is projected near the green polyhedra, favoring the inhibition activity on aromatase (Figure 6A–C). In the case of the electrostatic map, no substituents on **5b** are close to the red or blue polyhedron (Figure 6B), and so there is no increase or decrease in aromatase inhibition activity. Finally, in the hydrophobic map (Figure 6D), the 1,3,4-oxadiazole fragment bonded to the benzimidazole core is close to the yellow polyhedra, favoring the aromatase inhibition activity; however, the phenyl ring bonded to position 2 of benzimidazole core is mainly near the white polyhedron, causing a decrease in\aromatase inhibition activity.

## 3 Conclusion

In the present investigation, benzimidazole-1,3,4-oxadiazole derivatives **5a**–**i** have been synthesized and evaluated for their *in vitro* anticancer properties. The structures of synthesized compounds **5a**–**i** were characterized by using various modern analytical techniques such as ^1^H NMR, ^13^C NMR, and HRMS. Among the compounds, **5b**,**c** were found to be the most potent compounds against the MCF-7 cell line. According to the result of *in vitro* aromatase inhibitory activity, compounds **5b**,**c** cause 50% aromatase enzyme inhibition effectively at 1.475 ± 0.062 and 2.512 ± 0.124 μM, respectively. Molecular docking studies were performed to find the interaction types with the aromatase enzyme for compounds **5b**,**c**. Furthermore, a molecular dynamics simulation was performed out to explore the most likely binding mode of compounds **5b**,**c** with CYP19A1. Two types of satisfactory 3D-QSAR (CoMFA and CoMSIA) models were generated, to predict the inhibitory activities of novel inhibitors. In our cases, all of the training and test set molecules, from both CoMFA and CoMSIA, were found to be within the applicability domain.

## 4 Materials and Methods

Sigma-Aldrich Chemicals (Sigma-Aldrich Corp., St. Louis, MO, USA) and Merck Chemicals provided all of the chemicals used in the synthetic technique (Merck KGaA, Darmstadt, Germany). The MP90 digital melting point instrument (Mettler Toledo, Toledo, OH, USA) was used to determine the melting points of the compounds. Bruker 300 and 75 MHz digital FT-NMR spectrometers (Bruker Bioscience, Billerica, MA, USA) were used to record the ^1^H NMR and ^13^C NMR spectra of the produced compounds in DMSO-*d*_6_, respectively. In the NMR spectra, splitting patterns were identified as follows: s, singlet; d, doublet; t, triplet; m, multiplet. Coupling constants (*J*) are reported in hertz. Shimadzu LC/MS ITTOF equipment was used to determine M + 1 peaks (Shimadzu, Tokyo, Japan). All reactions were monitored using silica gel and thin-layer chromatography (TLC).

### 4.1 Chemistry

#### 4.1.1 Synthesis of 2-(4-Substituted phenyl)-1*H*-benzo[*d*]imidazole-6-carboxylic Acid Derivatives **1a**–**c**

In DMF, a combination of a 4-substituted benzaldehyde (0.03 mol) and sodium metabisulfide (0.03 mol, 5.7 g) was irradiated with microwaves for 5 min at 240 °C and 10 bar (Anton-Paar Monowave 300). After the mixture had cooled, 3,4-diaminobenzoic acid (0.03 mol, 4.56 g) was added and the reaction conditions were maintained. TLC was used to track the reaction’s progress. The mixture was put into crushed ice after the reaction was completed.

The filtered residue was washed with water, dried, and recrystallized from EtOH.^16^

#### 4.1.2 Synthesis of 2-Chloro-1-(4-substituted piperaz-1-yl)ethan-1-one Derivatives **1d**–**h**

In an ice bath, chloroacetyl chloride (0.014 mol, 1.056 mL) in THF (15 mL) was placed. Dropwise additions of 4-substituted piperazine derivatives (0.012 mol) and TEA (0.0132 mol, 1.90 mL) in THF (50 mL) were carried out. After the additions, the reaction mixture was stirred for 1 h at room temperature.^16^ The precipitated product was filtered, washed, and dried.

#### 4.2.3 Synthesis of Methyl 2-(4-Substituted phenyl)-1*H*-benzo[*d*]imidazole-6-carboxylate derivatives **2a**–**c**

In methanol, were dissolved compounds **1a**–**c** (0.025 mol), and several drops of sulfuric acid were added. The mixture was refluxed for 72 h before filtering the precipitate.^16^

#### 4.2.4 Synthesis of 2-(4-Substituted phenyl)-1*H*-benzo[*d*]imidazole-6-carbohydrazide derivatives **3a**–**c**

In a microwave synthesis reactor vial (30 mL), compounds **2a**–**c** (0.018 mol) were combined with an excess of hydrazine hydrate (5 mL) in EtOH (15 mL) and irradiated with microwaves (Anton-Paar Monowave 300). The reaction mixture was heated for 10 min at 240 °C and 10 bar. The mixture was cooled and then poured into ice water, filtered, rinsed with water, dried, and recrystallized from EtOH.^16^

#### 4.2.5 Synthesis of 2-((4-Substituted phenyl)-(6-(5-mercapto-1,3,4-oxadiazol-2-yl)-1*H*-benzo[*d*]imidazole Derivatives **4a**–**c**

In a solution of NaOH (0.01 mol, 0.4 g) in ethanol were dissolved compounds **3a**–**c** (0.01 mol), and carbon disulfide (0.01 mol, 0.60 mL) was added to the mixture. The mixture was refluxed for 8 h. The solution was cooled and acidified to pH 4–5 using concentrated hydrochloric acid solution after the reaction was completed to obtain the target compound.^16^

#### 4.2.6 General Synthesis Method of Target Compounds **5a**–**i**

In acetone were dissolved compounds **4a**–**c** (0.001 mol), potassium carbonate (0.001 mol, 0.138 g), and 2-chloro-1-(4-substituted epiperazin-1-yl)ethane-1-one (0.0015 mol) and refluxed for 6 h. The solvent was evaporated after TLC control, the residue was washed with water, dried, and then recrystallized from ethanol to produce final compounds **5a**–**i**.^16^

##### 4.2.6.1 2-((5-(2-(4-Hydroxyphenyl)-1*H*-benzo[*d*]imidazol-6-yl)-1,3,4-oxadiazol-2-yl)thio)-1-(4-benzylpiperazin-1-yl)ethan-1-one (**5a**)

Yield: 62%. Mp: 266.5–267.9 °C. ^1^H NMR (300 MHz, DMSO-d_6_): δ 3.40–3.50 (3H, m, piperazine), 3.67–3.87 (5H, m, piperazine), 4.18 (2H, s, −CH_2_), 4.61 (2H, s, −CH_2_), 6.97 (2H, d, *J* = 8.55 Hz, 1,4-disubstituted benzene), 7.33–7.41 (4H, m, aromatic CH), 7.57–7.59 (2H, m, aromatic CH), 7.58 (1H, d, *J* = 8.31 Hz, benzimidazole-C4), 7.79 (1H, dd, *J*_*1*_ = 8.52 Hz, *J*_*2*_ = 1.32 Hz, benzimidazole-C_5_), 8.13 (2H, d, *J* = 7.92 Hz, 1,4-disubstituted benzene), 10.31 (1H, s, O–H), 10.59 (1H, s, N–H). ^13^C NMR (75 MHz, DMSO-*d*_*6*_): δ 42.57, 49.01, 50.76, 59.66, 60.65, 72.69, 116.28, 116.66, 120.55, 120.73, 128.09, 128.82, 128.90, 129.09, 129.15, 129.43, 129.89, 130.26, 131.44, 154.70, 160.37, 163.05, 165.51, 166.51. HRMS (*m*/*z*): [M + H]^+^ calcd for C_28_H_26_N_6_O_3_S, 527.1859; found, 527.1860.

##### 4.2.6.2 2-((5-(2-(4-Hydroxyphenyl)-1*H*-benzo[*d*]imidazol-6-yl)-1,3,4-oxadiazol-2-yl)thio)-1-(4-(4-chlorobenzyl)piperazin-1-yl)ethan-1-one (**5b**)

Yield: 68%. Mp: 155.5–157.4 °C. ^1^H NMR (300 MHz, DMSO-*d*_6_): δ 3.04–3.05 (4H, m, piperazine), 3.84–3.86, (4H, m, piperazine), 4.27 (2H, s, −CH_2_), 4.61 (2H, s, −CH_2_), 6.97 (2H, d, *J* = 8.61 Hz, 1,4-disubstituted benzene), 7.48 (2H, d, *J* = 8.19 Hz, 1,4-disubstituted benzene), 7.59–7.62 (2H, m, aromatic CH), 7.71 (1H, d, *J* = 8.37 Hz, benzimidazole-C_4_), 7.80 (1H, dd, *J*_*1*_ = 8.43 Hz, *J*_*2*_ = 1.14 Hz, benzimidazole-C_5_), 8.11–8.14 (3H, m, aromatic CH), 10.27 (1H, s, O–H), 10.63 (1H, s, NH). ^13^C NMR (75 MHz, DMSO-*d*_6_): δ 43.39, 50.81, 51.28, 58.79, 60.67, 72.72, 116.28, 116.71, 117.21, 118.29, 120.50, 120.76, 121.52, 129.07, 129.17, 130.64, 131.02, 133.22, 134.14, 154.64, 160.39, 163.05, 165.48, 166.48. HRMS (*m*/*z*): [M + H]^+^/2 calcd for C_28_H_25_N_6_O_3_SCl, 281.0768; found, 281.0771.

##### 4.2.6.3 2-((5-(2-(4-Hydroxyphenyl)-1*H*-benzo[*d*]imidazol-6-yl)-1,3,4-oxadiazol-2-yl)thio)-1-(4-(4-fluorobenzyl)piperazin-1-yl)ethan-1-one (**5c**)

Yield: 71%. Mp: >400 °C. ^1^H NMR (300 MHz, DMSO-d_6_): δ 3.48–3.53 (8H, m, piperazine), 3.95 (2H, s, −CH_2_), 4.54 (2H, s, −CH_2_), 6.86–6.89 (4H, m, aromatic CH), 7.12 (1H, d, *J* = 8.82 Hz, benzimidazole-C4), 7.30 (1H, s, benzimidazole-C7), 7.65–7.66 (2H, m, aromatic CH), 7.69–7.71 (1H, m, aromatic CH), 8.04 (2H, d, *J* = 8.52 Hz, 1,4-disubstituted benzene). ^13^C NMR (75 MHz, DMSO-d_6_): δ 45.86, 52.40, 52.91, 60.67, 61.28, 72.73, 61.28, 113.54, 115.39 (d, *J* = 20.96 Hz), 115.72, 116.65, 128.80, 129.01, 131.18 (d, *J* = 7.73 Hz), 134.40 (d, *J* = 2.78 Hz), 143.77, 156.07, 156.49, 161.76 (d, *J* = 240.93 Hz), 162.36, 162.86, 165.11, 166.03, 166.79, 168.20. HRMS (*m*/*z*): [M + H]^+^ calcd for C_28_H_25_N_6_O_3_FS, 545.1740; found, 545.1766.

##### 4.2.6.4 2-((5-(2-(4-Methoxyphenyl)-1*H*-benzo[*d*]imidazol-6-yl)-1,3,4-oxadiazol-2-yl)thio)-1-(4-benzylpiperazin-1-yl)ethan-1-one (**5d**)

Yield: 74%. Mp: 172.6–174.8 °C. ^1^H NMR (300 MHz, DMSO-*d*_6_): δ3.29 (4H, br.s., piperazine), 3.53 (4H, br s, piperazine), 3.86 (3H, s, −OCH_3_), 4.35 (2H, s, −CH_2_), 4.63 (2H, s, −CH_2_), 7.16 (2H, d, *J* = 8.94 Hz, 1,4-disubstituted benzene), 7.44–7.47 (3H, m, aromatic CH), 7.63–7.66 (2H, m, aromatic CH), 7.77 (1H, d, *J* = 8.49 Hz, benzimidazole-C_4_), 7.87 (1H, dd, *J*_*1*_ = 8.43 Hz, *J*_*2*_ = 1.56 Hz, benzimidazole-C_5_), 8.14–8.16 (1H, m, aromatic CH), 8.27 (2H, d, *J* = 8.85 Hz, 1,4-disubstituted benzene). ^13^C NMR (75 MHz, DMSO-*d*_6_): δ 36.71, 42.61, 45.77, 50.35, 50.80, 55.96, 59.03, 113.21, 113.79, 115.09, 115.91, 117.58, 120.82, 121.57, 128.58, 129.24, 129.43, 129.96, 130.37, 131.96, 153.57, 162.09, 163.28, 165.60, 166.21. HRMS (*m*/*z*): [M + H]^+^ calcd for C_29_H_28_N_6_O_3_S, 541.2002; found, 541.2016.

##### 4.2.6.5 2-((5-(2-(4-Methoxyphenyl)-1*H*-benzo[*d*]imidazol-6-yl)-1,3,4-oxadiazol-2-yl)thio)-1-(4-(4-chlorobenzyl)piperazin-1-yl)ethan-1-one (**5e**)

Yield: 78%. Mp: 138.2–140.1 °C. ^1^H NMR (300 MHz, DMSO-*d*_6_): δ3.57–3.64 (4H, m, piperazine), 3.73 (4H, br.s., piperazine), 3.84 (3H, s, -OCH_3_), 4.23–4.26 (2H, m, −CH_2_), 4.57 (2H, s, −CH_2_), 7.12 (2H, d, *J* = 8.91 Hz, 1,4-disubstituted benzene), 7.39–7.42 (4H, m, aromatic CH), 7.72 (1H, d, *J* = 8.43 Hz, benzimidazole-C_4_), 7.79 (1H, dd, *J*_1_ = 8.46 Hz, *J*_2_ = 1.47 Hz, benzimidazole-C_5_), 8.13 (1H, s, benzimidazole-C_7_), 8.22 (2H, d, *J* = 8.79 Hz, 1,4-disubstituted benzene). ^13^C NMR (75 MHz, DMSO-*d*_6_): δ 36.96, 44.95, 45.72, 51.83, 52.31, 55.87, 60.39, 114.90, 115.72, 116.78, 116.81, 120.79, 120.81, 122.41, 128.80, 129.00, 131.88, 132.02, 132.79, 154.29, 161.52, 163.17, 165.23, 166.45, 169.47. HRMS (*m*/*z*): [M + H]^+^ calcd for C_29_H_27_N_6_O_3_SCl, 575.1624; found, 575.1627.

##### 4.2.6.6 2-((5-(2-(4-Methoxyphenyl)-1*H*-benzo[*d*]imidazol-6-yl)-1,3,4-oxadiazol-2-yl)thio)-1-(4-(4-fluorobenzyl)piperazin-1-yl)ethan-1-one (**5f**)

Yield: 62%. Mp: 133.4–135.8 °C. ^1^H NMR (300 MHz, DMSO-*d*_6_): δ3.65 (8H, br.s., piperazine), 3.86 (3H, s, -OCH_3_), 4.24 (2H, s, −CH_2_), 4.61 (2H, s, −CH_2_), 7.10–7.13 (3H, m, aromatic CH), 7.39–7.40 (2H, m, aromatic CH), 7.70–7.72 (3H, m, aromatic CH), 8.01 (1H, s, aromatic CH), 8.21–8.24 (2H, m, aromatic CH). ^13^C NMR (75 MHz, DMSO-*d*_*6*_): δ 34.79, 42.63, 45.72, 48.94, 55.85, 55.88, 60.46, 114.91, 115.56 (d, *J* = 21.15 Hz), 116.28, 120.39, 120.79, 122.26, 129.05, 132.21, 132.33, 134.82 (d, *J* = 7.84 Hz), 133.73 (d, *J* = 2.79 Hz), 154.37, 160.47, 161.57, 163.07, 163.69, 167.99 (d, *J* = 219.83 Hz), 177.65. HRMS (*m*/*z*): [M + H] calcd for C_29_H_27_N_6_O_3_FS, 559.1895; found, 559.1922.

##### 4.2.6.7 2-((5-(2-(4-Ethoxyphenyl)-1*H*-benzo[*d*]imidazol-6-yl)-1,3,4-oxadiazol-2-yl)thio)-1-(4-benzylpiperazin-1-yl)ethan-1-one (**5g**)

Yield: 72%. Mp: 184.4–186.8 °C. ^1^H NMR (300 MHz, DMSO-*d*_6_): δ1.24 (3H, t, *J* = 7.08 Hz, −CH_3_), 3.38–3.50 (8H, br s, piperazine), 3.83 (2H, s, −CH_2_), 4.02–4.09 (2H, m, CH_2_), 4.32 (2H, s, −CH_2_), 7.07 (2H, d, *J* = 8.91 Hz, aromatic CH), 7.27–7.32 (6H, m, aromatic CH), 7.66 (1H, d, *J* = 8.28 Hz, aromatic CH), 7.75 (1H, s, aromatic CH), 8.23 (2H, d, *J* = 8.70 Hz, aromatic CH). ^13^C NMR (75 MHz, DMSO-*d*_6_): δ 15.71, 37.38, 42.09, 45.91, 52.53, 53.27, 62.26, 64.56, 114.60, 115.59, 119.63, 121.62, 124.66, 127.23, 127.50, 128.56, 128.70, 129.20, 129.34, 138.28, 149.47, 156.57, 160.78, 165.85.

##### 4.2.6.8 2-((5-(2-(4-Ethoxyphenyl)-1*H*-benzo[*d*]imidazol-6-yl)-1,3,4-oxadiazol-2-yl)thio)-1-(4-(4-chlorobenzyl)piperazin-1-yl)ethan-1-one (**5h**)

Yield: 73%. Mp: 177.5–178.4 °C. ^1^H NMR (300 MHz, DMSO-*d*_6_): δ1.35 (3H, t, *J* = 6.96 Hz, −CH_3_), 3.49–3.53 (8H, br.s., piperazine), 4.06–4.08 (2H, m, CH_2_), 4.10–4.11 (2H, m, CH_2_), 4.53 (2H, s, −CH_2_), 7.01–7.05 (3H, m, aromatic CH), 7.31–7.38 (2H, m, aromatic CH), 7.61–7.67 (2H, m, aromatic CH), 8.05–8.08 (1H, m, aromatic CH), 8.18–8.22 (3H, m, aromatic CH). ^13^C NMR (75 MHz, DMSO-*d*_6_): δ 15.11, 36.98, 42.23, 45.86, 52.45, 52.93, 61.27, 63.63, 113.64, 114.14, 114.92, 115.01, 116.08, 119.14, 119.45, 125.23, 128.64, 128.81, 131.11, 132.01, 137.37, 159.97, 162.52, 165.14, 167.16, 167.96. HRMS (*m*/*z*): [M + H]^+^ calcd for C_30_H_29_N_6_O_3_SCl, 589.1791; found, 589.1783.

##### 4.2.6.9 2-((5-(2-(4-Ethoxyphenyl)-1*H*-benzo[*d*]imidazol-6-yl)-1,3,4-oxadiazol-2-yl)thio)-1-(4-(4-fluorobenzyl)piperazin-1-yl)ethan-1-one (**5i**)

Yield: 67%. Mp: 189.9–191.4 °C. ^1^H NMR (300 MHz, DMSO-*d*_6_): δ1.35 (3H, t, *J* = 6.93 Hz, −CH_3_), 2.31-.34 (2H, m, −CH_2_), 3.49–3.53 (8H, m, piperazine), 4.10 (2H, q, *J* = 6.93 Hz, −CH_2_), 4.53 (2H, s, −CH_2_), 7.03 (2H, d, *J* = 8.94 Hz, 1,4-disubstituted benzene), 7.11–7.17 (2H, m, aromatic CH), 7.32–7.36 (2H, m, aromatic CH), 7.62–7.65 (1H, m, Benzimidazole-C_4_), 7.68 (1H, dd, *J*_*1*_ = 8.7 Hz, *J*_*2*_ = 1.59 Hz, benzimidazole-C_5_), 8.08 (1H, s, benzimidazole-C_7_), 8.20 (2H, d, *J* = 8.88 Hz, 1,4-disubstituted benzene). ^13^C NMR (75 MHz, DMSO-*d*_6_): δ 15.10, 36.99, 42.23, 45.86, 52.40, 52.90, 61.28, 63.66, 114.06, 114.99, 115.19, 115.39 (d, *J* = 20.93 Hz), 116.06, 119.48, 124.62, 128.84, 131.19 (d, J= 7.95 Hz), 134.41 (d, *J* = 2.86 Hz), 142.45, 145.00, 157.06, 160.14, 162.67, 165.12, 165.19 (d, *J* = 272.33 Hz). HRMS (*m*/*z*): [M + H]^+^ calcd for C_30_H_29_N_6_O_3_FS, 573.2079; found, 573.2062.

### 4.2 Cytotoxicity Assay

MTT tests were used to screen the anticancer activities of compounds **5a**–**i**. The MTT assays were carried out as stated previously.^16,17^ The anticancer activity of the final compounds was tested on five cancer cell lines: A549 (lung carcinoma cell line), HeLa (cervical cell line), MCF-7 (human breast adenocarcinoma cell line), HepG2 (human liver carcinoma cell line), C6 (rat glioma cell line), and NIH3T3 (human embryonic kidney cell line) (mouse embryo fibroblast cell line). In the MTT experiments, doxorubicin and Hoechst 33342 were employed as reference drugs.

### 4.3 Aromatase Inhibition Assay

The BioVision Aromatase (CYP19A) Inhibitor Screening Kit (Fluorometric) was used to carry out this experiment.

### 4.4 Molecular Docking

The human placental aromatase cytochrome P450 protein structure (PDB ID: 3EQM) (https://www.rcsb.org/structure/3EQM) was imported into UCSF Chimera 1.15 software.^27^ Except for HEM, heteroatoms in the protein crystal structure were removed. Compound 2D structures were drawn with ChemDraw Professional 19.0 software, and minimized 3D structures were created using a universal force field. CYP19A1 enzyme and compound structures were converted to the pdbqt file format using the PyRx 0.8 software. On the basis of the ASD cocrystal ligand in the CYP19A1 crystal structure; the coordinates of the active site were determined as *x* 85.7, *y* 54.1, *z* 46.1, and a 20 × 20 × 20 Å^3^ grid box was formed. The molecular docking study was carried out with AutoDock Vina 1.1.2^28^ via PyRx software. The analysis and visualization of 2D and 3D protein–ligand interactions were performed with BIOVIA Discovery Studio Visualizer v2021.

### 4.5 Molecular Dynamics

Molecular dynamics simulations were performed to investigate the CYP19A1–**5b** and CYP19A1–**5b** protein–ligand complex stability using the Gromacs 2020.4 version (Groningen Machine for Chemical Simulations).^29^ The topology file of CYP19A1 including the HEM structure was created with the Charmm36 force field, and ligand topology files were created via the CHARMM General Force Field (CGenFF) server.^30^ The topology files for CYP19A1 and compounds **5b**,**c** were combined and solved with the TIP3 water model, and the appropriate Na^+^ and Cl^–^ ions were added. The energy of the system that formed CYP19A1, HEM, ligand, ion, and solvent was minimized. The system was equilibrated with 0.2 ns NVT and 0.3 ns NPT stages at 1 atm pressure and 300 K temperature according to a V-rescale thermostat and Berendsen barostat, respectively, and 100 ns molecular dynamics simulations were performed to 2 fs. The trajectory analysis was performed with gmx scripts, and the root-mean-square deviation (RMSD) and the root-mean-square fluctuation (RMSF) were measured. The trajectory results were visualized with VMD (Visual Molecular Dynamics), and RMSD and RMSF graphs were created with GraphPad Prism 8 software. The binding free energy calculation by the molecular mechanics Poisson–Boltzmann surface area (MM-PBSA) was calculated between 80 and 100 ns using RashmiKumari’s g_mmpbsa package.^31^

### 4.6 3D-QSAR Study

3D-QSAR studies were performed with Sybyl X-1.2 software installed in a Windows 10 environment on a PC with an Intel core i7 CPU, according to previous reports of our research group.^18−20^ The details of the procedure, data set, statistical values, and the validation of models are given in the Supporting Information.

## Supporting Information Available

^1^H NMR, ^13^C NMR, and mass spectra of compounds **5a**–**i** and the QSAR method (PDF)
