# Supplementary material for: Design, Synthesis, and Molecular Modeling Studies of a Novel Benzimidazole as an Aromatase Inhibitor
Source: ACS Omega. 2022 Apr 28;7(18):16152–63. doi: 10.1021/acsomega.2c01497 (PMC9097188; doi:10.1021/acsomega.2c01497)
Supplement: Supplementary file 1 — ao2c01497_si_001.pdf [file ao2c01497_si_001.pdf]

Design, synthesis, molecular modeling studies of novel benzimidazole as an aromatase inhibitor

Ulviye Acar Çevik [1,], Ismail Celik*[2], Jaime Mella[3], Marco Mellado[4], Yusuf Özkay[1], Zafer Asım Kaplancıklı[1]

[1] Department of Pharmaceutical Chemistry, Faculty of Pharmacy, Anadolu University, Eskişehir 26470, Turkey.

[2] Department of Pharmaceutical Chemistry, Faculty of Pharmacy, Erciyes University, Kayseri 38039, Turkey.

[3] Institute of Chemistry and Biochemistry, Faculty of Sciences, University of Valparaíso, Av. Great Britain, 1111 Valparaíso, Chile.

[4] Institute of Chemistry, Faculty of Sciences, Pontificia Universidad Católica de Valparaíso. Av. Universidad 330, Curauma, Valparaíso.

* Corresponding Author. E-mail: ismailcelik@erciyes.edu.tr

Address: Erciyes University, Faculty of Pharmacy, Department of Pharmaceutical Chemistry, 38039, Kayseri, Turkey.

Contents:

Mass, ^{1}H-NMR, and ^{13}C-NMR spectrums of all compounds……………………………………………..……S2-S18

Qsar Methods details……………………………………………………………………………………….S19-S42

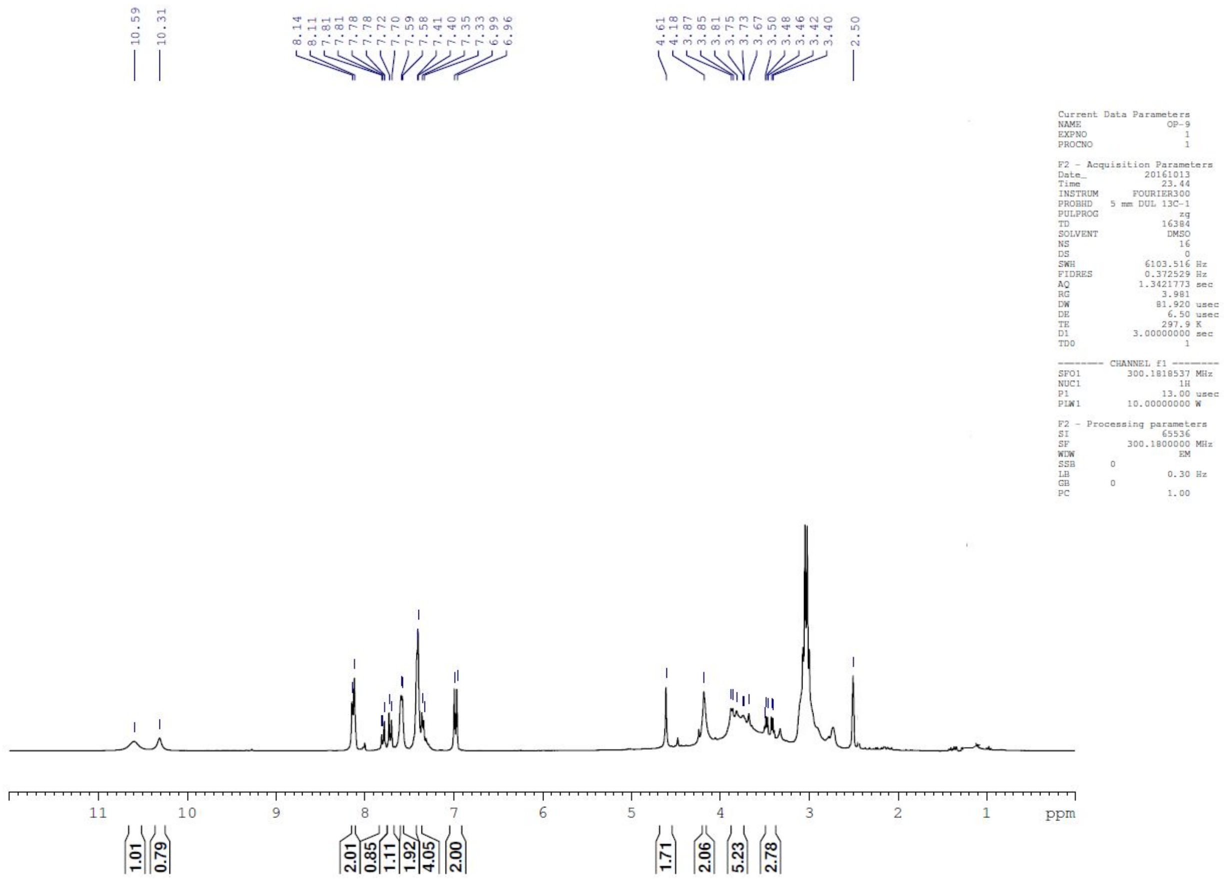

Figure s1. ^{1}H-NMR spectrum of compound **5a**

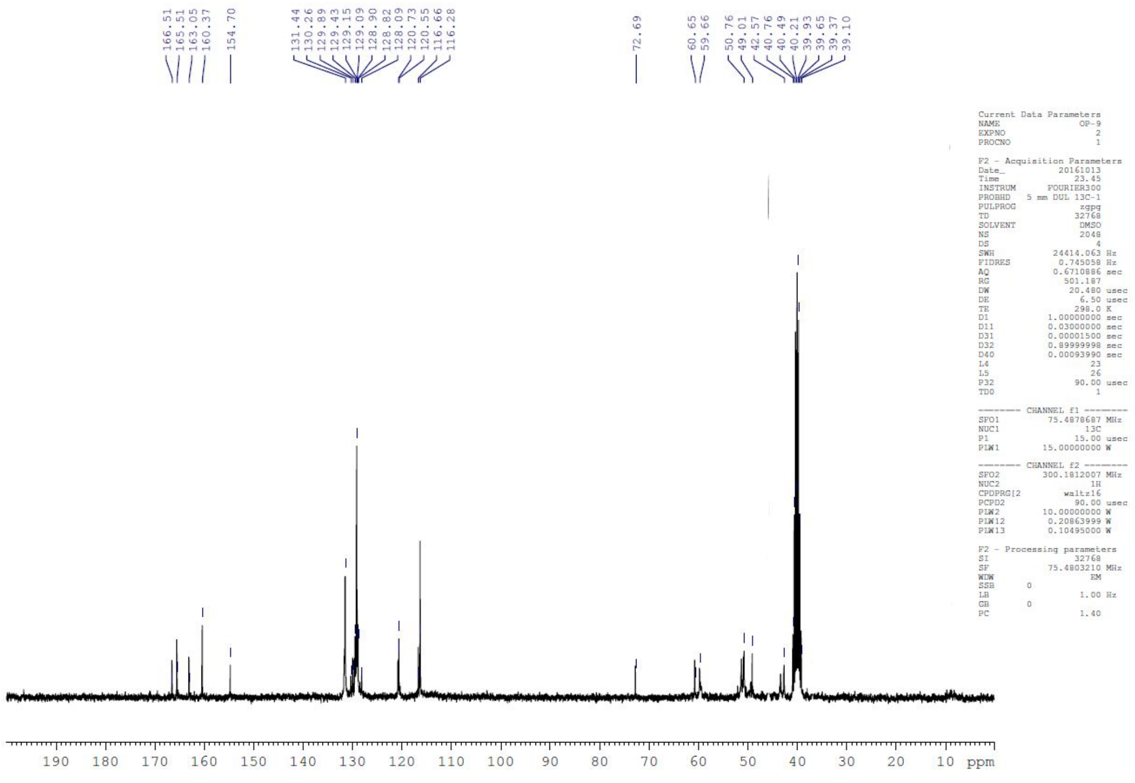

Figure s2. ^{13}C-NMR spectrum of compound **5a**

Error Margin (ppm):	5	DBE Range:	12.0 - 20.0	Electron Ions:	both
HC Ratio:	unlimited	Apply N Rule:	yes	Use MSn Info:	yes
Max Isotopes:	3	Isotope RI (%):	1.00	Isotope Res:	9000
MSn Iso RI (%):	10.00	MSn Logic Mode:	AND	Max Results:	500

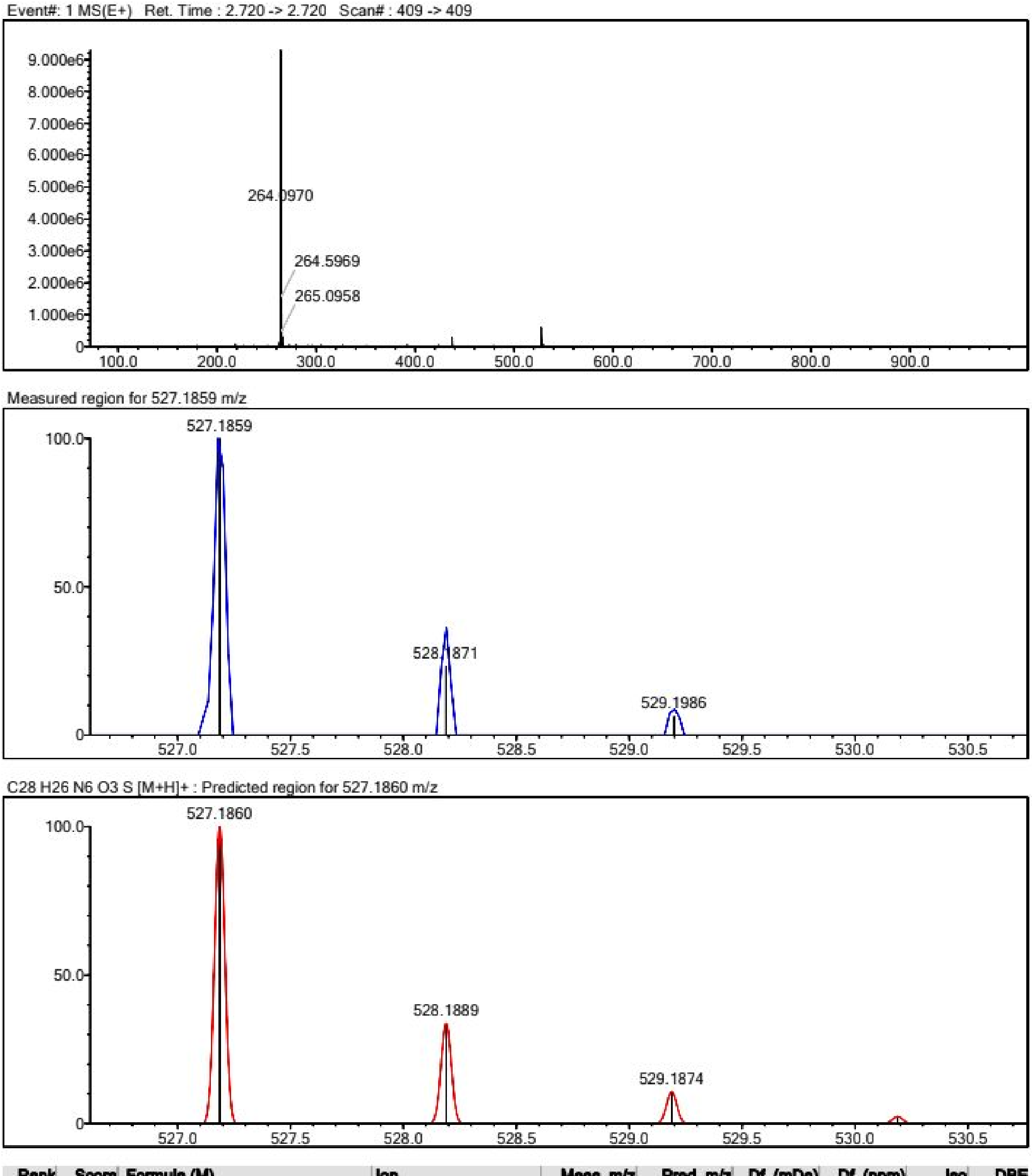

Rank	Score	Formula (M)	Ion	Meas. m/z	Pred. m/z	Df. (mDa)	Df. (ppm)	Iso	DBE
1	69.25	C28 H26 N6 O3 S	[M+H]+	527.1859	527.1860	-0.1	-0.19	69.25	19.0

Figure s3. Mass spectrum of compound **5a**

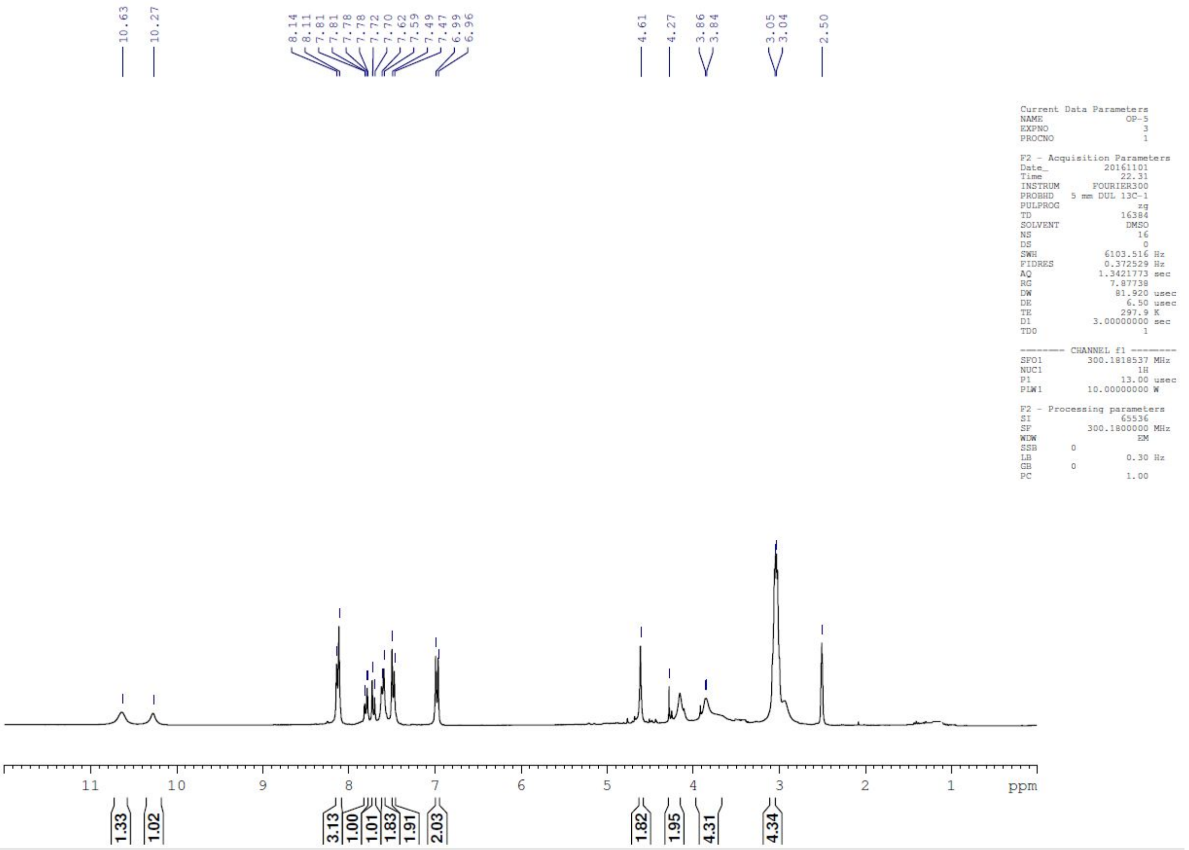

Figure s4. ^{1}H-NMR spectrum of compound **5b**

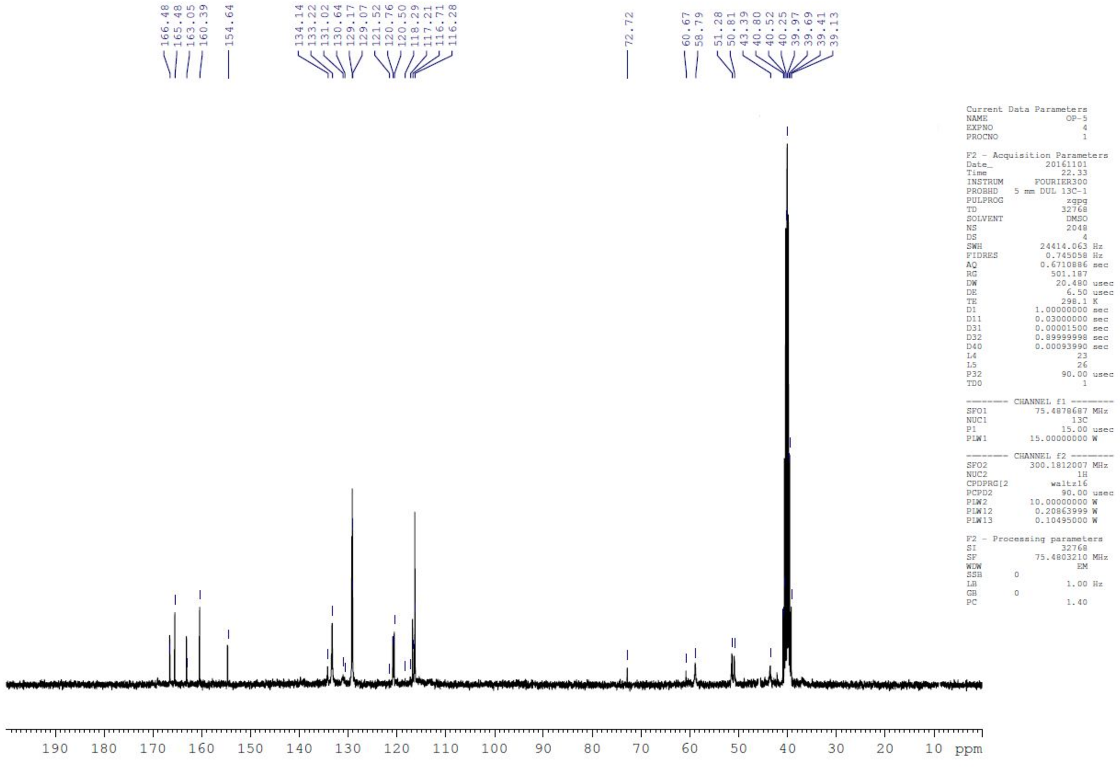

Figure s5. ^{13}C-NMR spectrum of compound **5b**

Error Margin (ppm):	5	DBE Range:	12.0 - 20.0	Electron Ions:	both
HC Ratio:	unlimited	Apply N Rule:	yes	Use MSn Info:	yes
Max Isotopes:	3	Isotope RI (%):	1.00	Isotope Res:	9000
MSn Iso RI (%):	10.00	MSn Logic Mode:	AND	Max Results:	500

Event#: 1 MS(E+) Ret. Time : 3.627 Scan# : 545

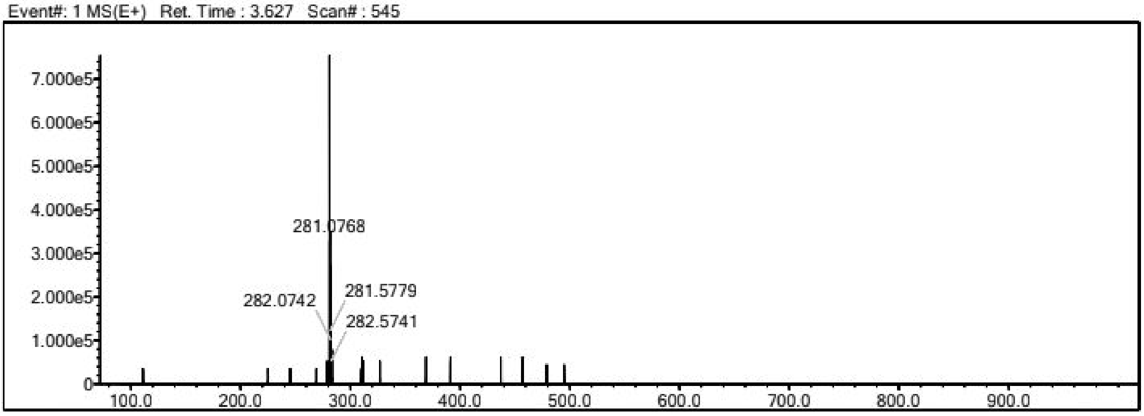

Measured region for 281.0768 m/z

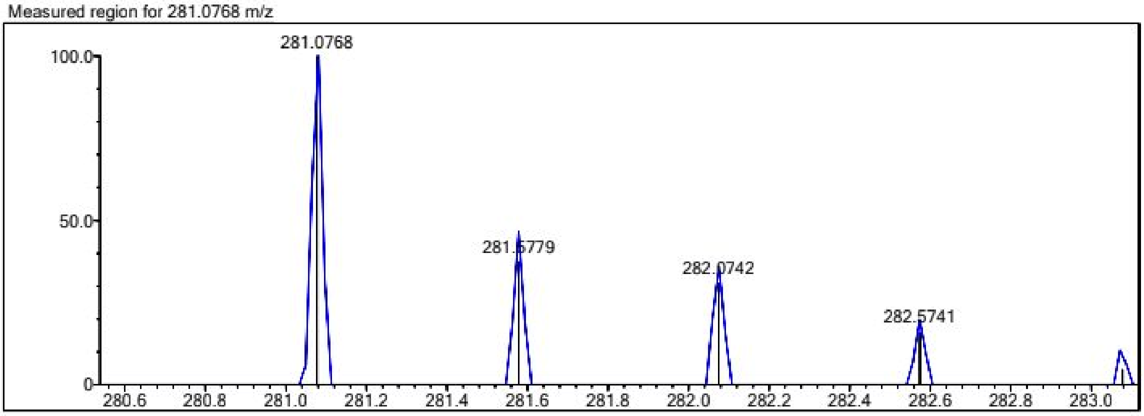

C28 H25 N6 O3 S Cl [M+2H]2+ : Predicted region for 281.0771 m/z

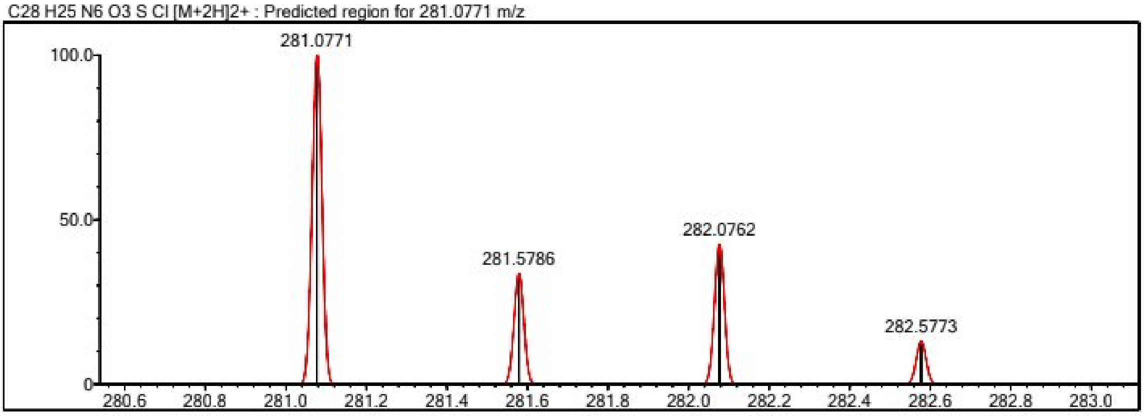

Rank	Score	Formula (M)	Ion	Meas. m/z	Pred. m/z	Df. (mDa)	Df. (ppm)	Iso	DBE
1	60.56	C28 H25 N6 O3 S Cl	[M+2H]2+	281.0768	281.0771	-0.3	-1.07	60.66	19.0

Figure s6. Mass spectrum of compound **5b**

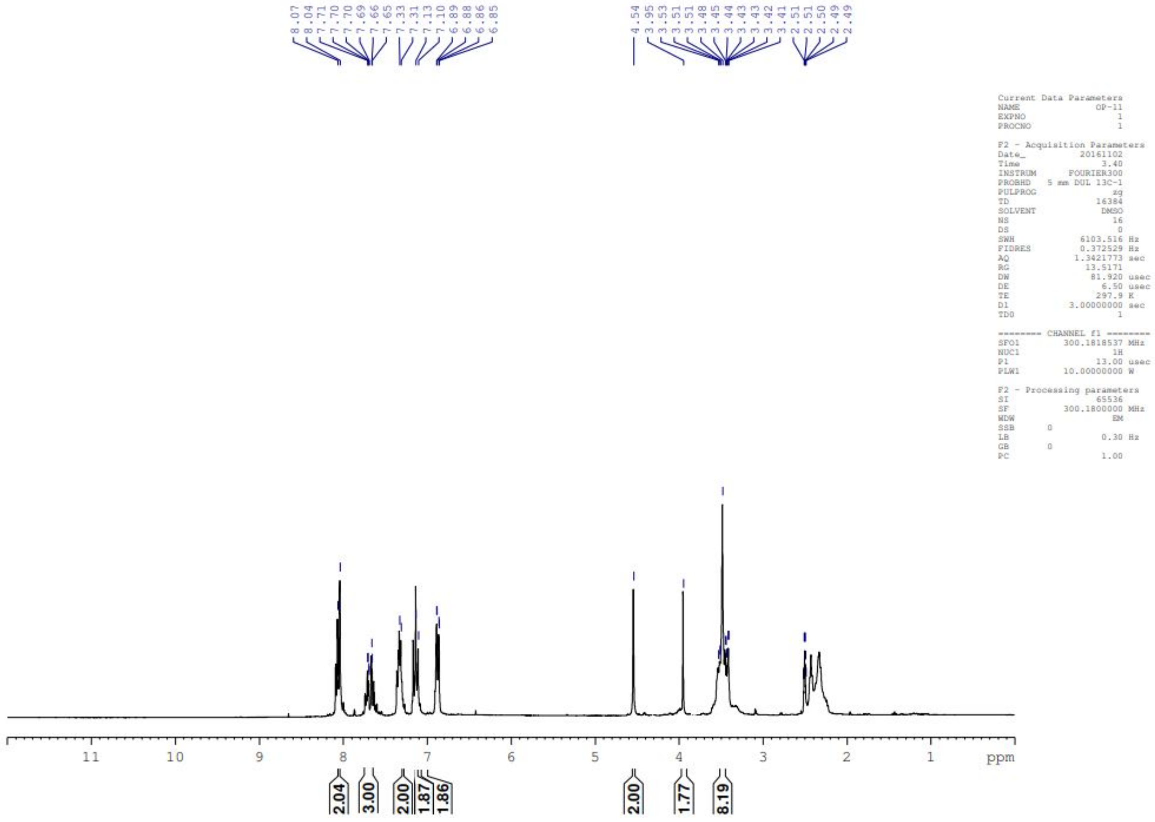

Figure s7. ^{1}H-NMR spectrum of compound **5c**

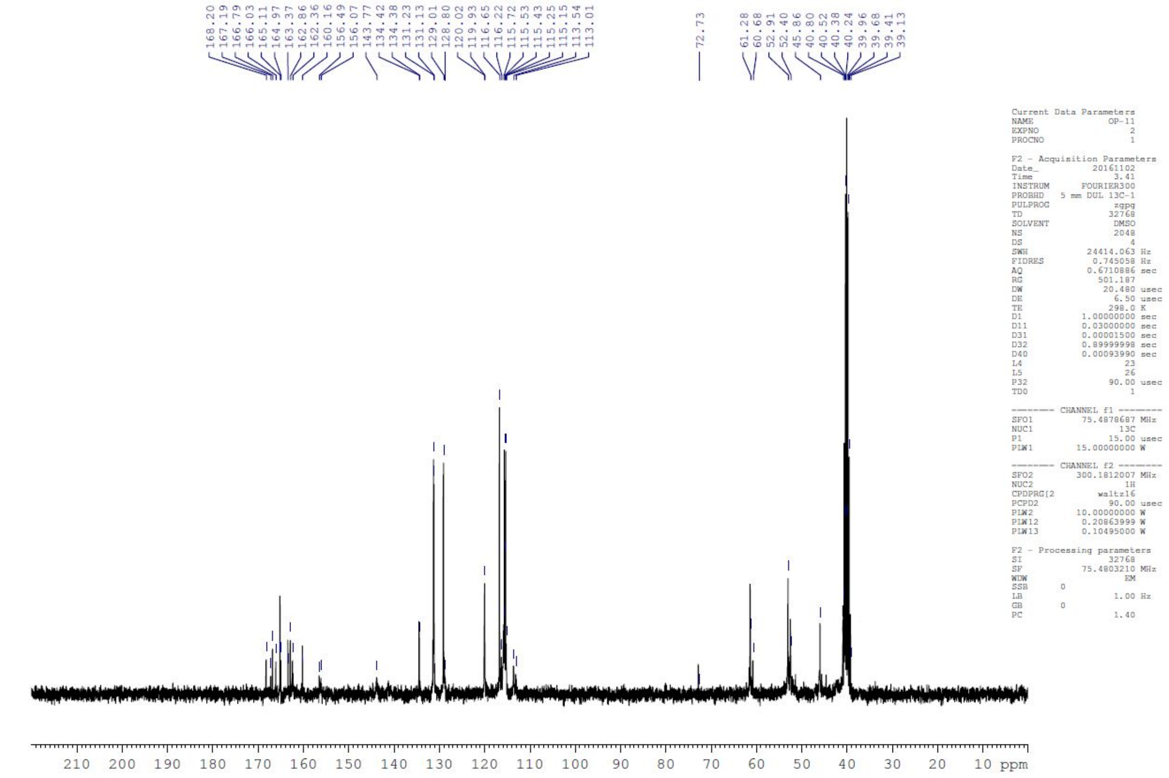

Figure s8. ^{13}C-NMR spectrum of compound **5c**

Error Margin (ppm):	5	DBE Range:	16.0 - 20.0	Electron Ions:	both
HC Ratio:	unlimited	Apply N Rule:	yes	Use MSn Info:	yes
Max Isotopes:	3	Isotope RI (%):	1.00	Isotope Res:	9000
MSn Iso RI (%):	10.00	MSn Logic Mode:	AND	Max Results:	500

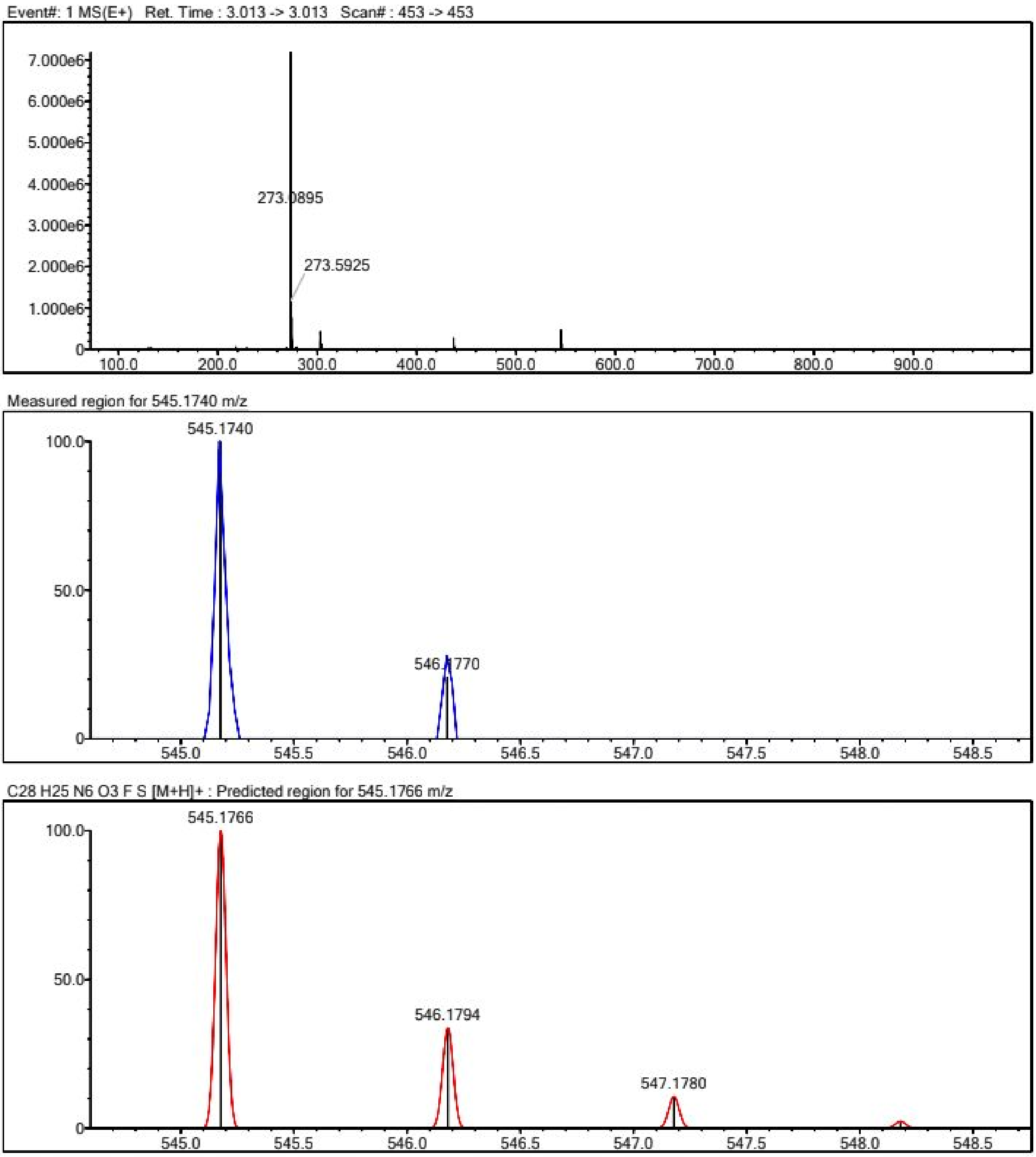

Rank	Score	Formula (M)	Ion	Meas. m/z	Pred. m/z	Df. (mDa)	Df. (ppm)	Iso	DBE
1	0.00	C28 H25 N6 O3 F S	[M+H]+	545.1740	545.1766	-2.6	-4.77	0.00	19.0

Figure s9. Mass spectrum of compound **5c**

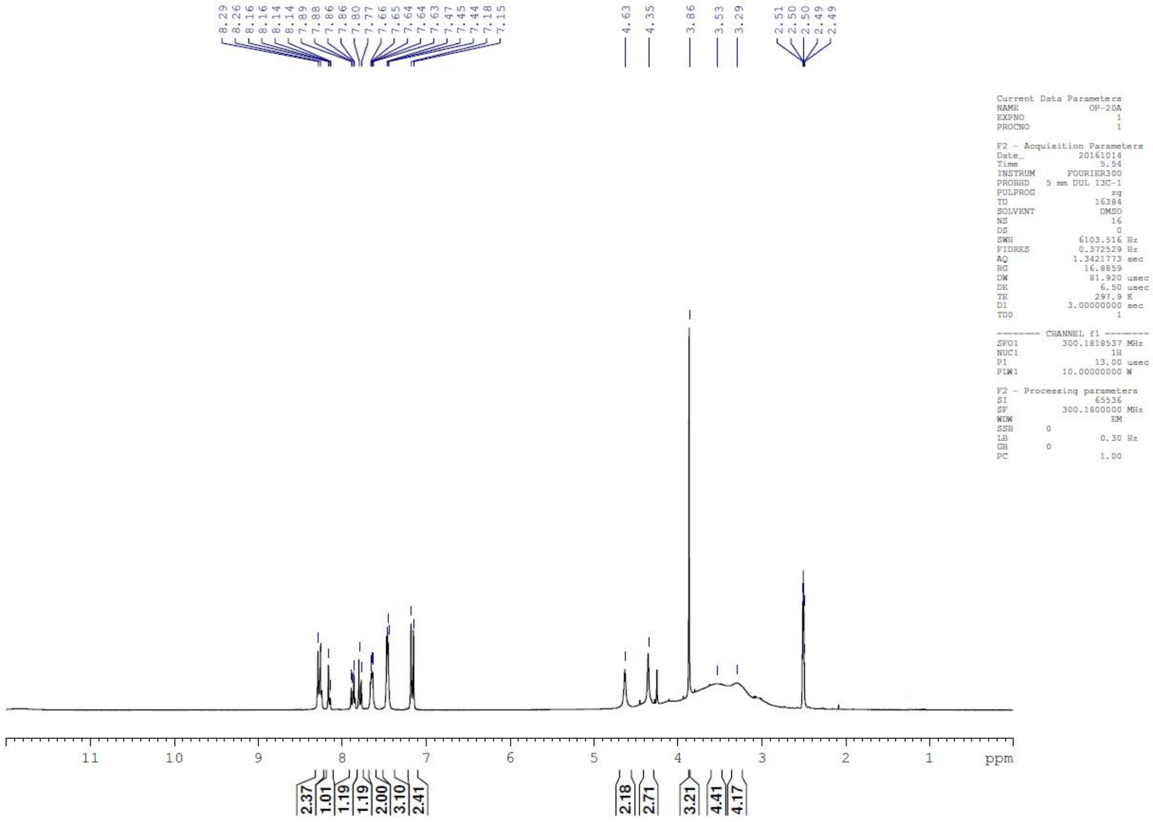

Figure s10. ^{1}H-NMR spectrum of compound **5d**

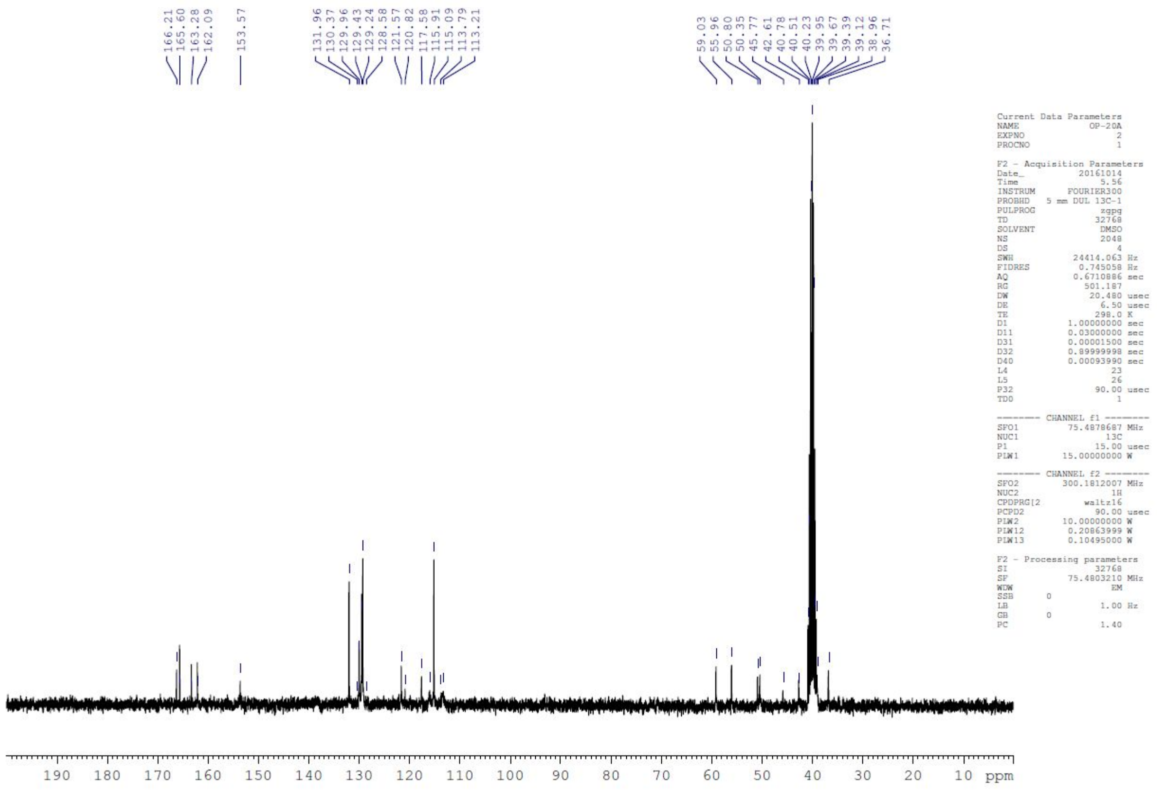

Figure s11. ^{13}C-NMR spectrum of compound **5d**

Error Margin (ppm): 5 | DBE Range: 16.0 - 20.0 | Electron Ions: both
HC Ratio: unlimited | Apply N Rule: yes | Use MSn Info: yes
Max Isotopes: 3 | Isotope RI (%): 1.00 | Isotope Res: 9000
MSn Iso RI (%): 10.00 | MSn Logic Mode: AND | Max Results: 500

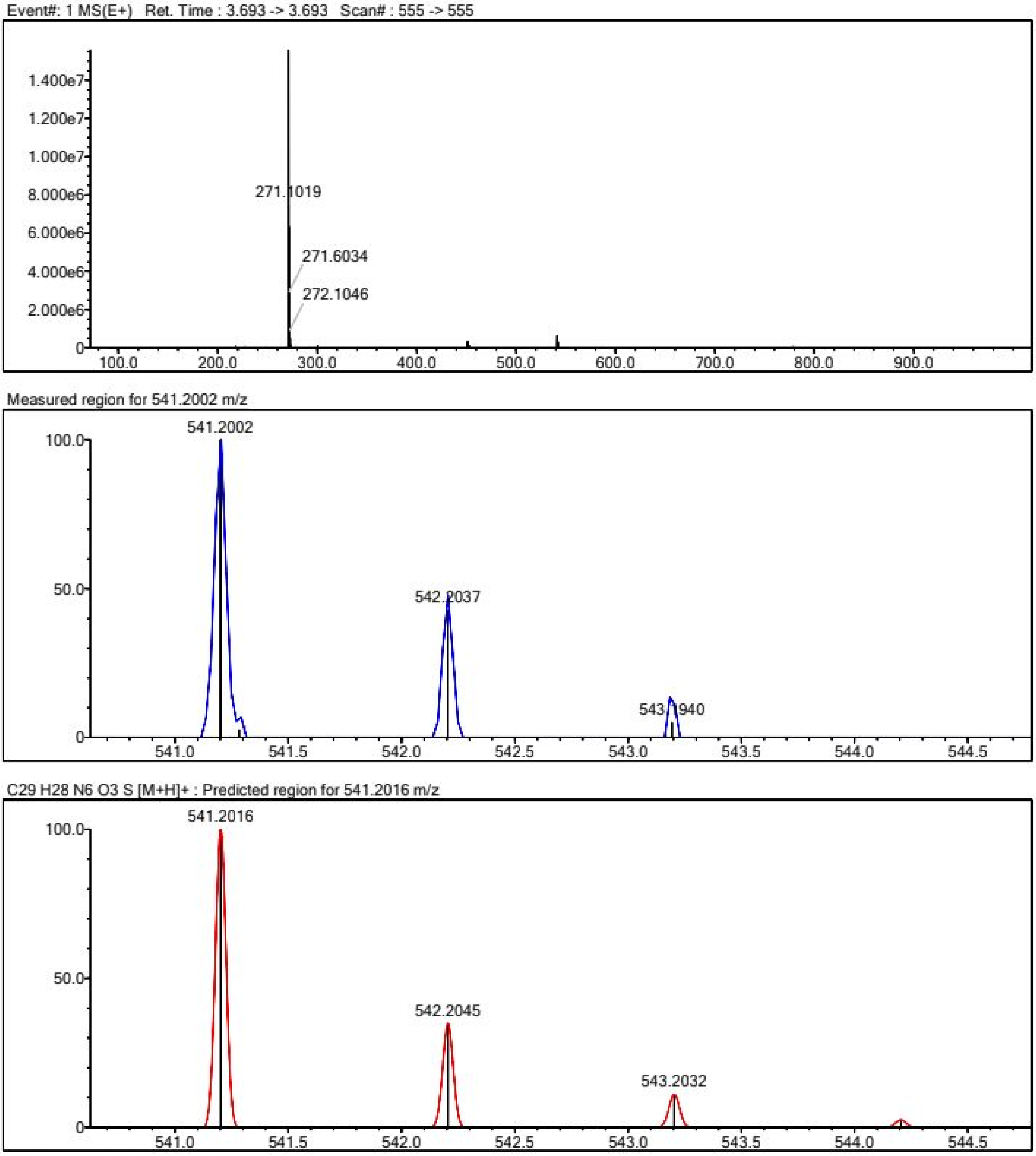

Rank	Score	Formula (M)	Ion	Meas. m/z	Pred. m/z	Df. (mDa)	Df. (ppm)	Iso	DBE
1	45.66	C29 H28 N6 O3 S	[M+H]+	541.2002	541.2016	-1.4	-2.59	47.55	19.0

Figure s12. Mass spectrum of compound **5d**

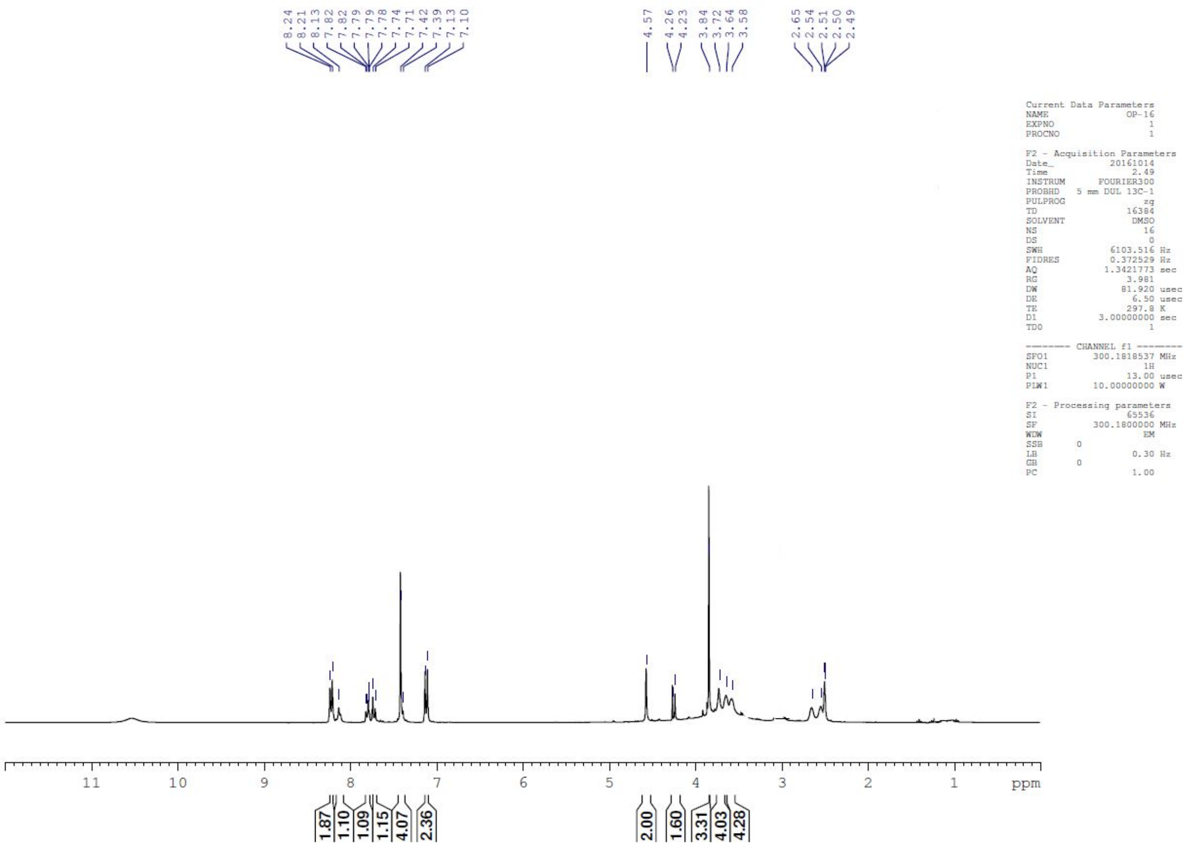

Figure s13. ^{1}H-NMR spectrum of compound **5e**

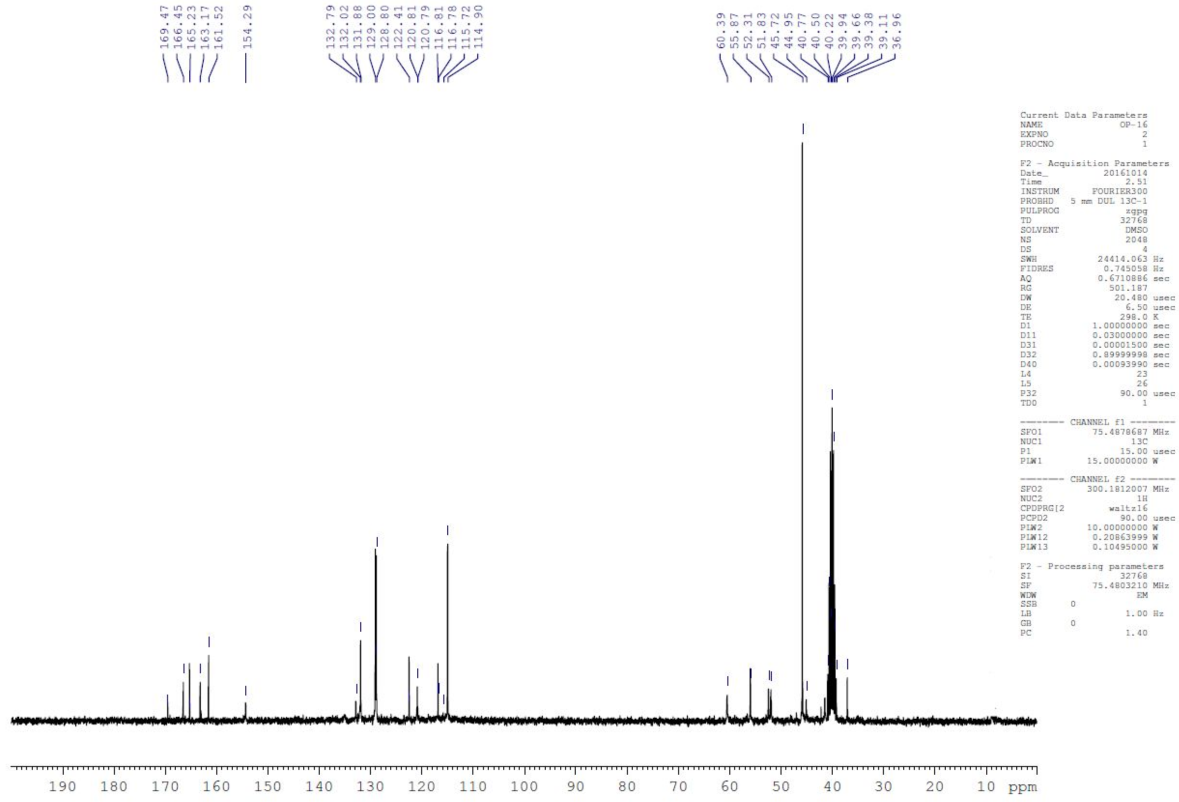

Figure s14. ^{13}C-NMR spectrum of compound **5e**

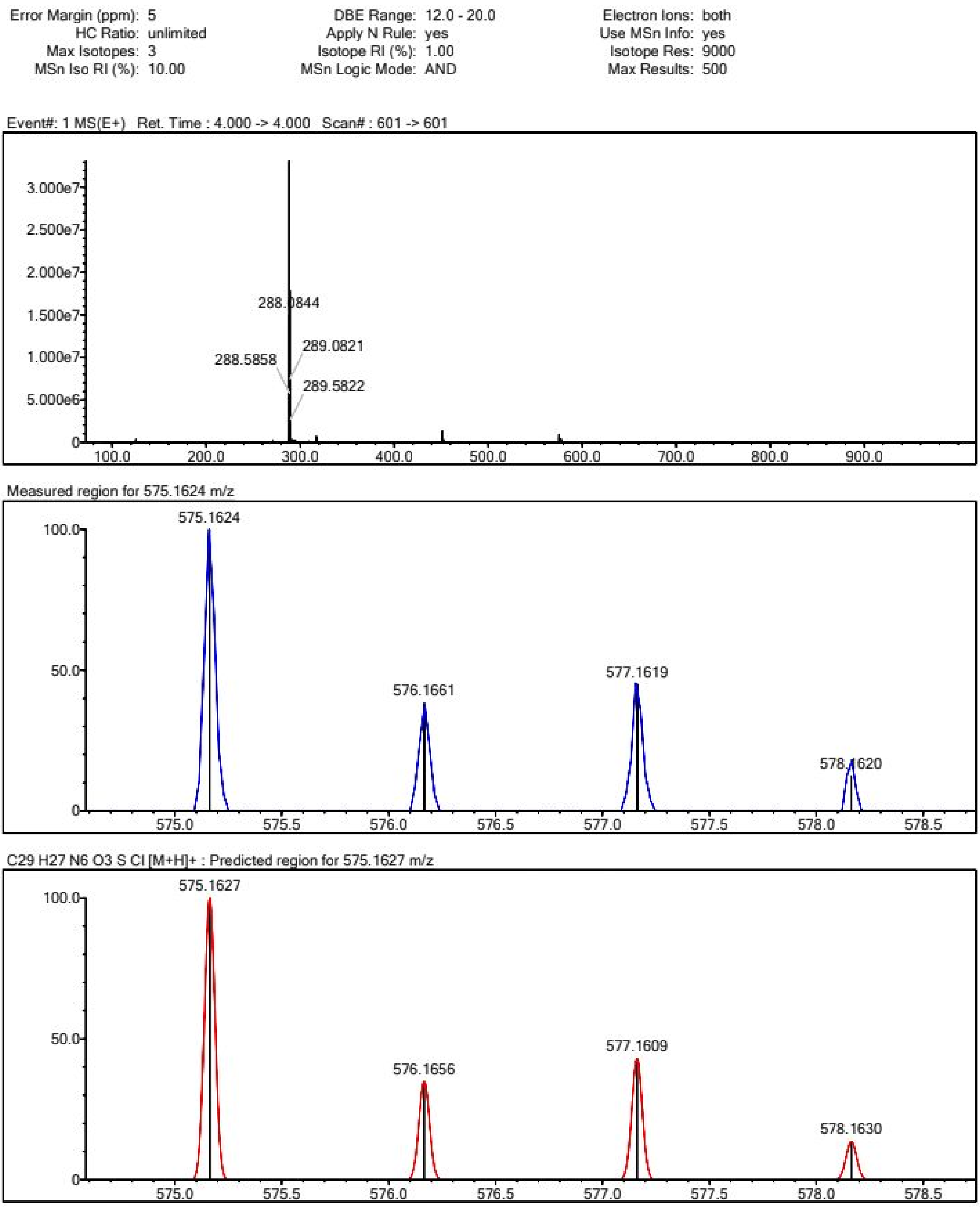

Rank	Score	Formula (M)	Ion	Meas. m/z	Pred. m/z	Df. (mDa)	Df. (ppm)	Iso	DBE
1	82.78	C29 H27 N6 O3 S Cl	[M+H]+	575.1624	575.1627	-0.3	-0.52	82.78	19.0

Figure s15. Mass spectrum of compound **5e**

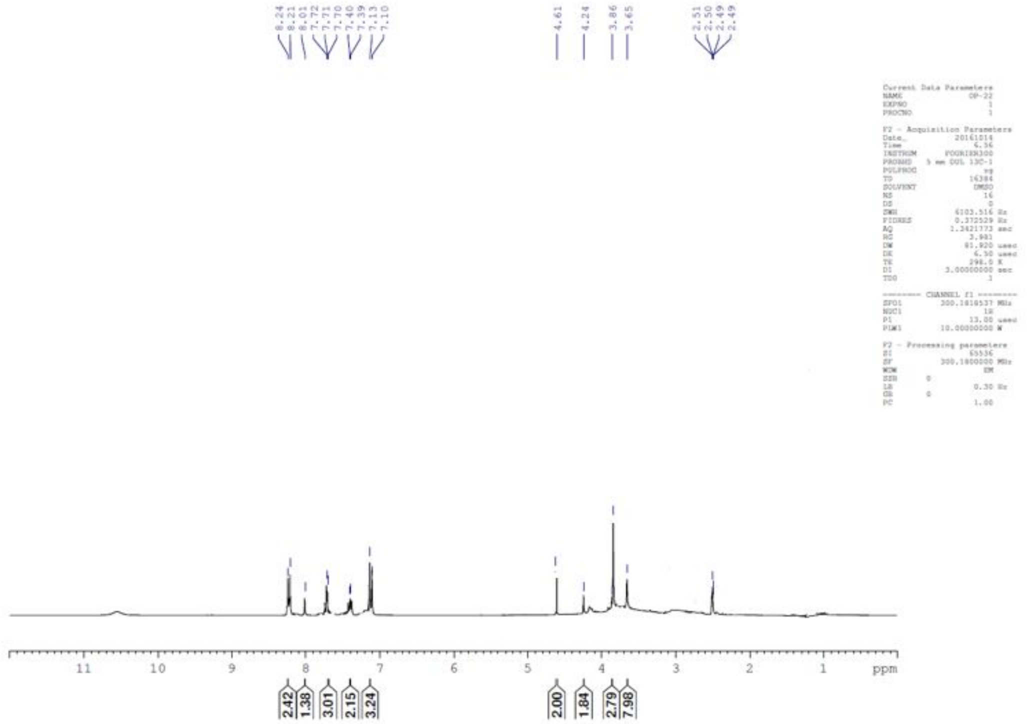

Figure s16. ^{1}H-NMR spectrum of compound **5f**

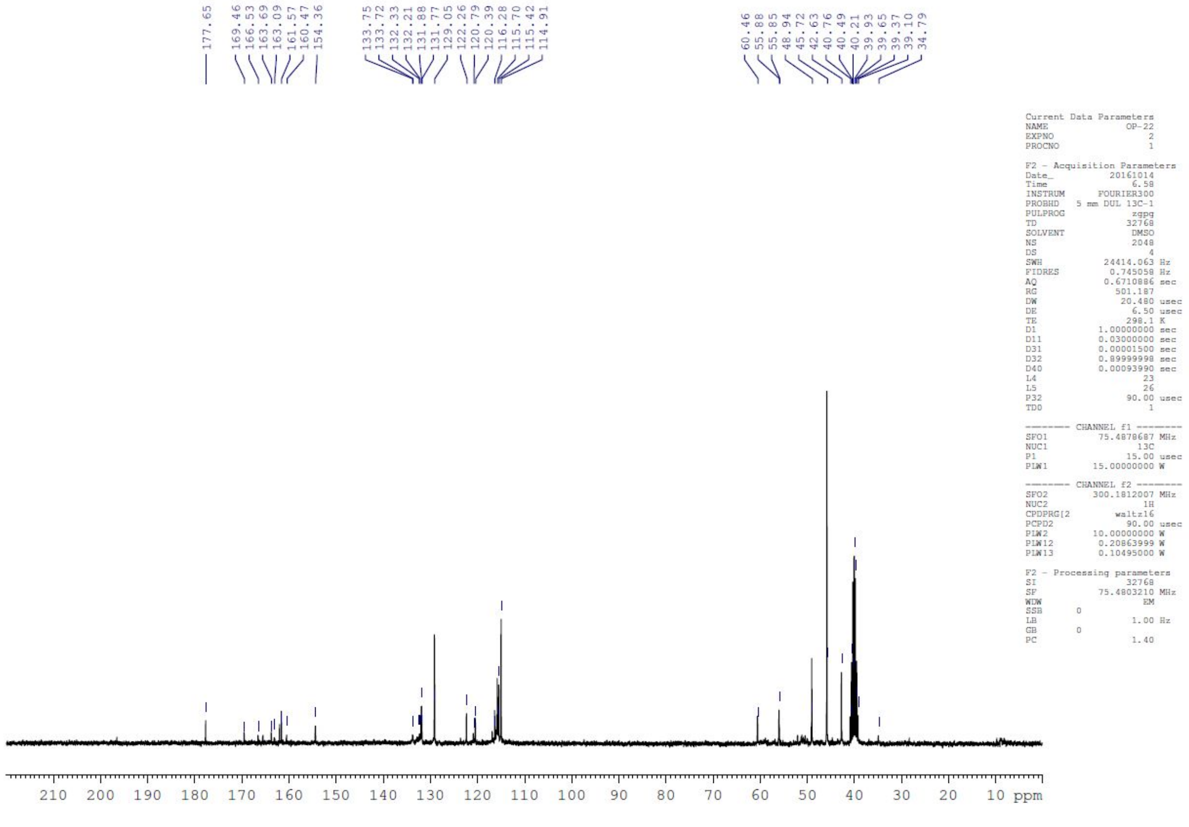

Figure s17. ^{13}C-NMR spectrum of compound **5f**

Error Margin (ppm):	15	DBE Range:	16.0 - 20.0	Electron Ions:	both
HC Ratio:	unlimited	Apply N Rule:	yes	Use MSn Info:	yes
Max Isotopes:	3	Isotope RI (%):	1.00	Isotope Res:	9000
MSn Iso RI (%):	10.00	MSn Logic Mode:	AND	Max Results:	500

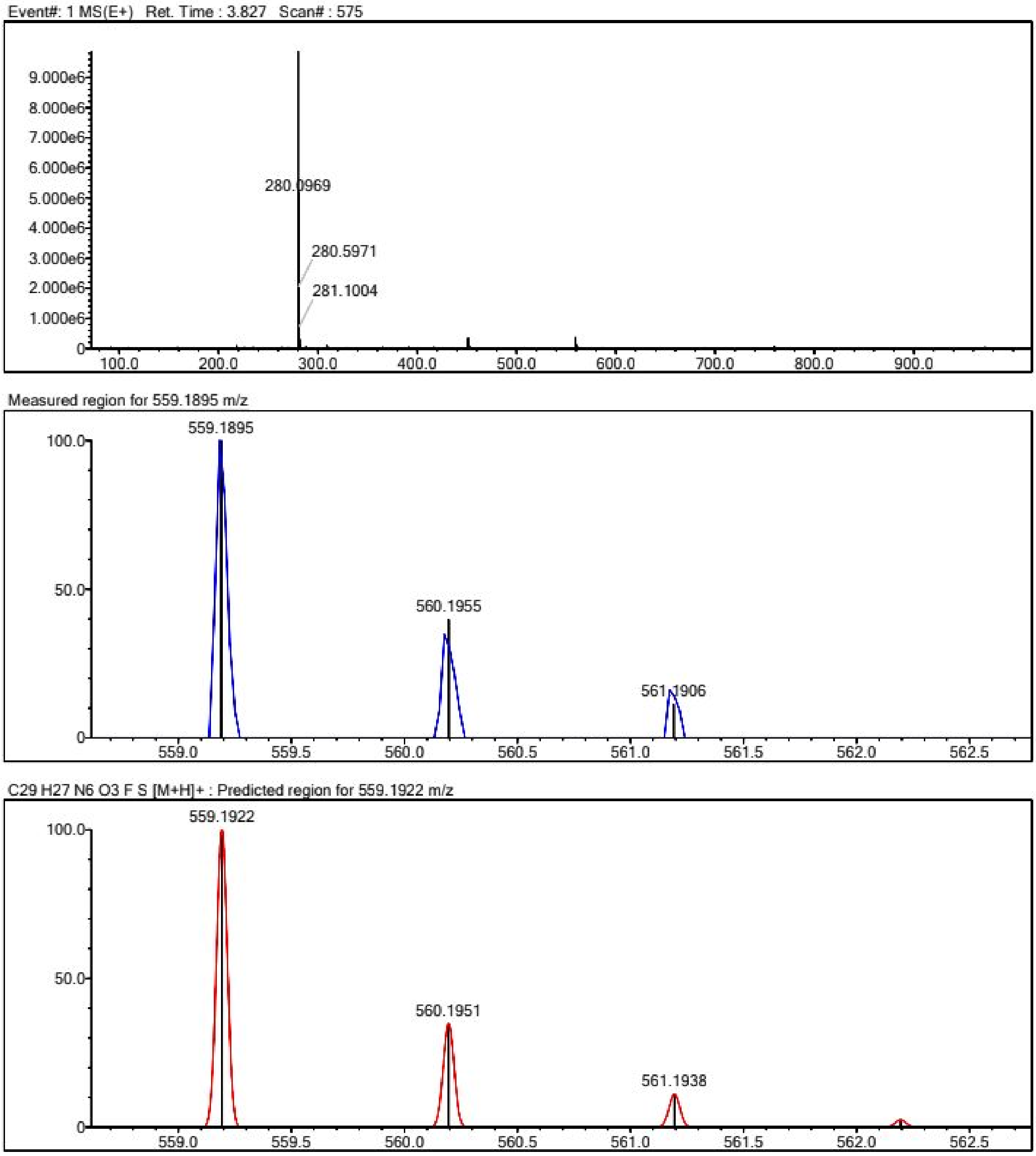

Rank	Score	Formula (M)	Ion	Meas. m/z	Pred. m/z	Df. (mDa)	Df. (ppm)	Iso	DBE
1	48.63	C29 H27 N6 O3 F S	[M+H]+	559.1895	559.1922	-2.7	-4.83	53.78	19.0

Figure s18. Mass spectrum of compound **5f**

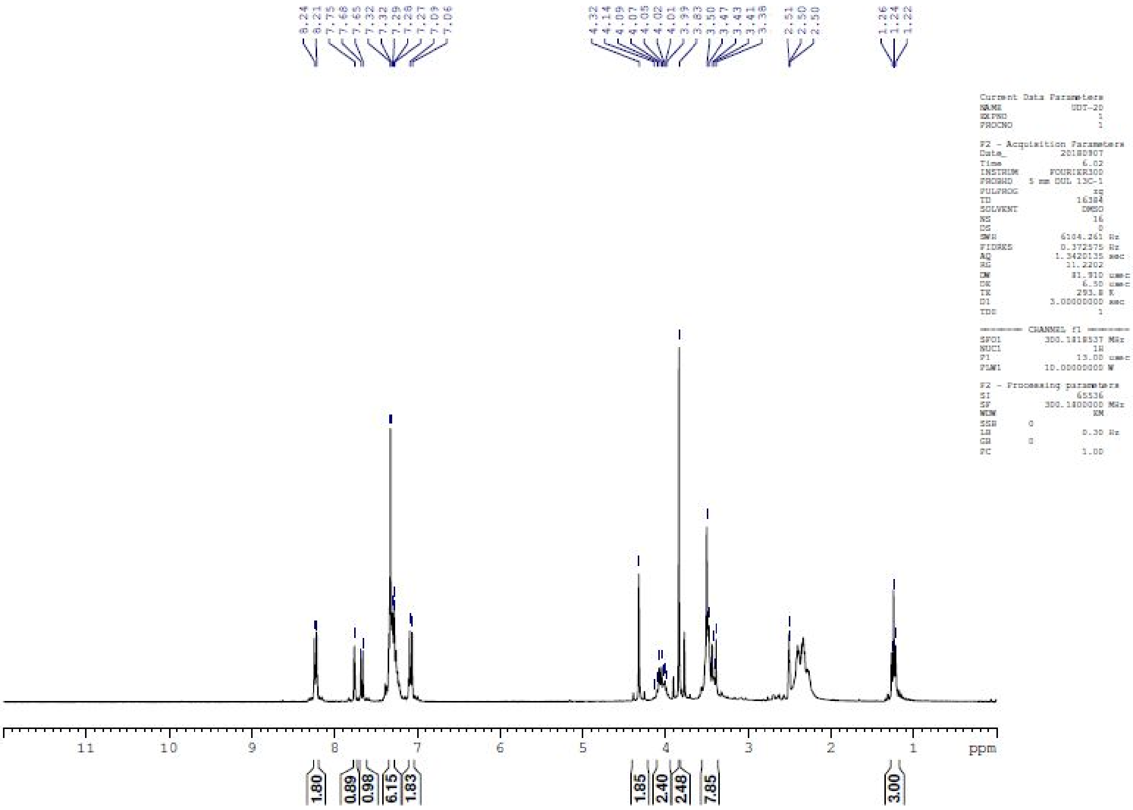

Figure s19. ^{1}H-NMR spectrum of compound **5g**

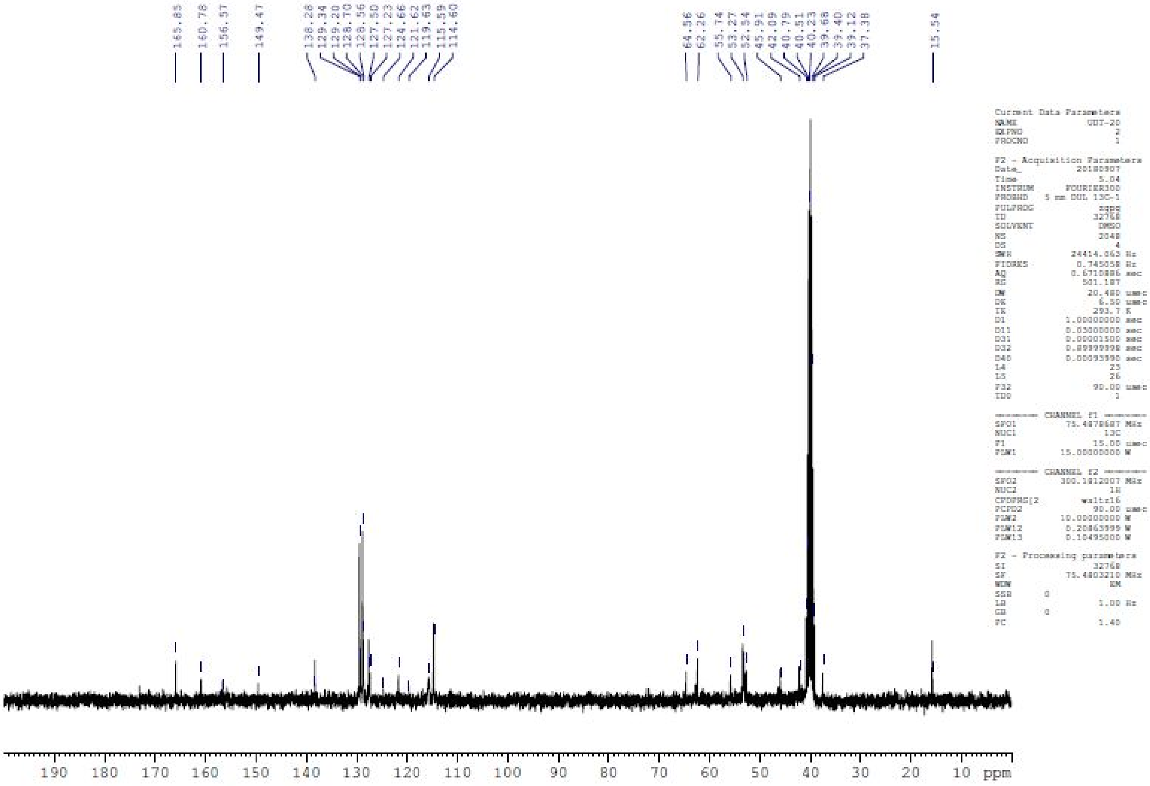

Figure s20. ^{13}C-NMR spectrum of compound **5g**

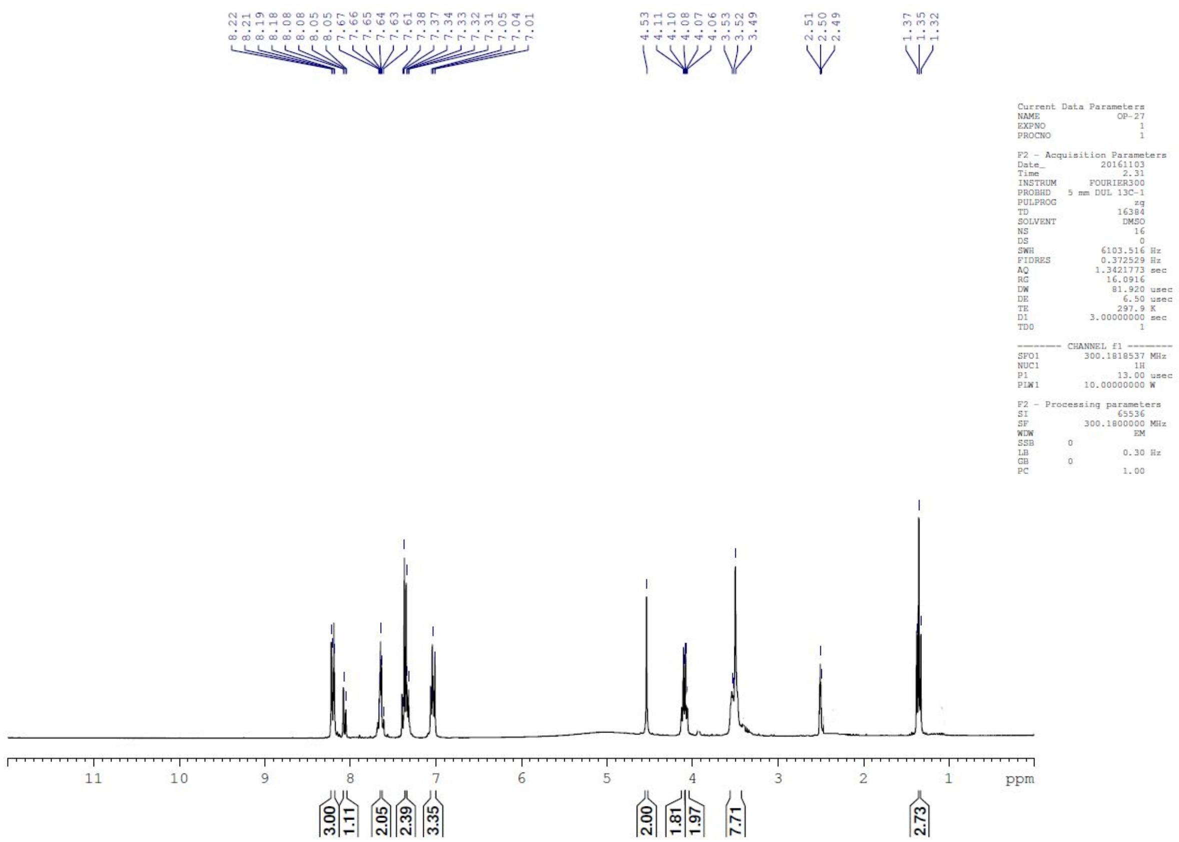

Figure s21. ^{1}H-NMR spectrum of compound **5h**

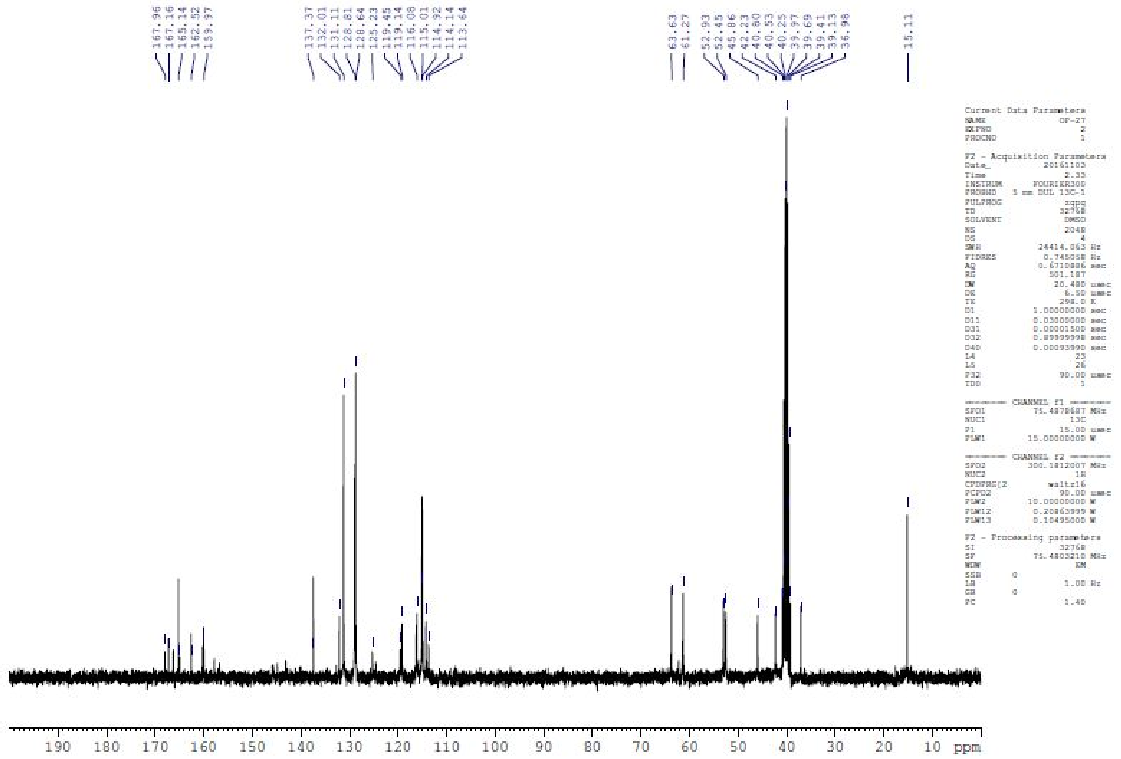

Figure s22. ^{13}C-NMR spectrum of compound **5h**

Error Margin (ppm): 5
HC Ratio: unlimited
Max Isotopes: 3
MSn Iso RI (%): 10.00

DBE Range: 16.0 - 20.0
Apply N Rule: yes
Isotope RI (%): 1.00
MSn Logic Mode: AND

Electron Ions: both
Use MSn Info: yes
Isotope Res: 9000
Max Results: 500

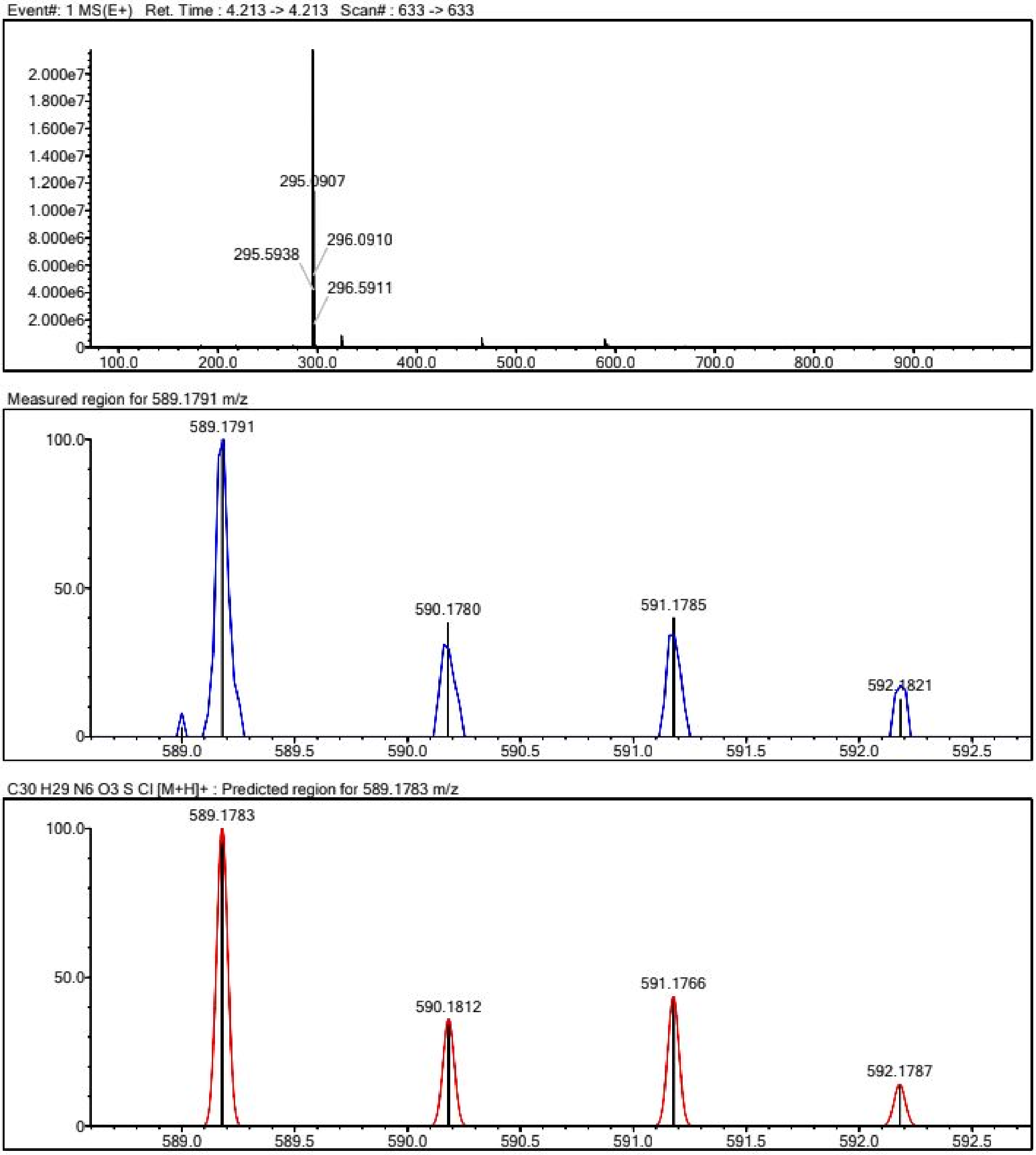

Rank	Score	Formula (M)	Ion	Meas. m/z	Pred. m/z	Df. (mDa)	Df. (ppm)	Iso	DBE
1	66.27	C30 H29 N6 O3 S Cl	[M+H]+	589.1791	589.1783	0.8	1.36	66.87	19.0

Figure s23. Mass spectrum of compound **5h**

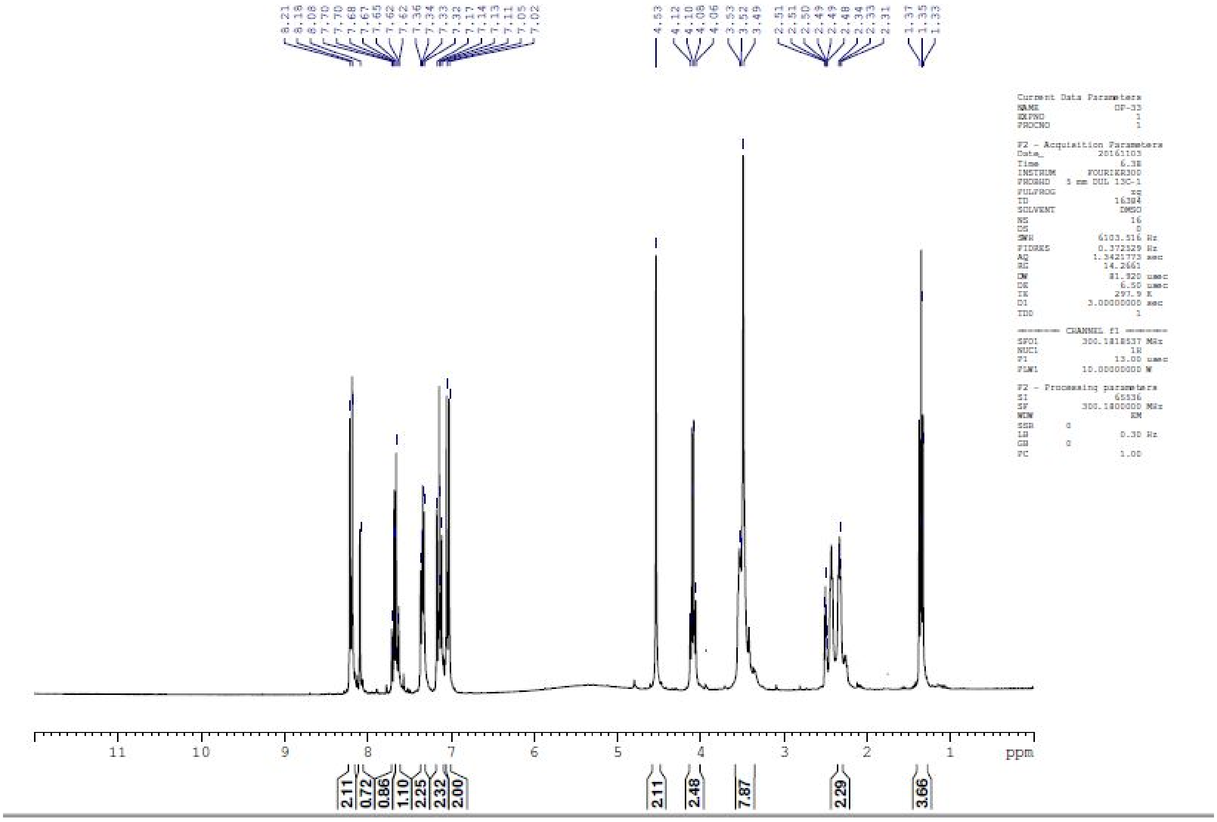

Figure s24. ^{1}H-NMR spectrum of compound **5i**

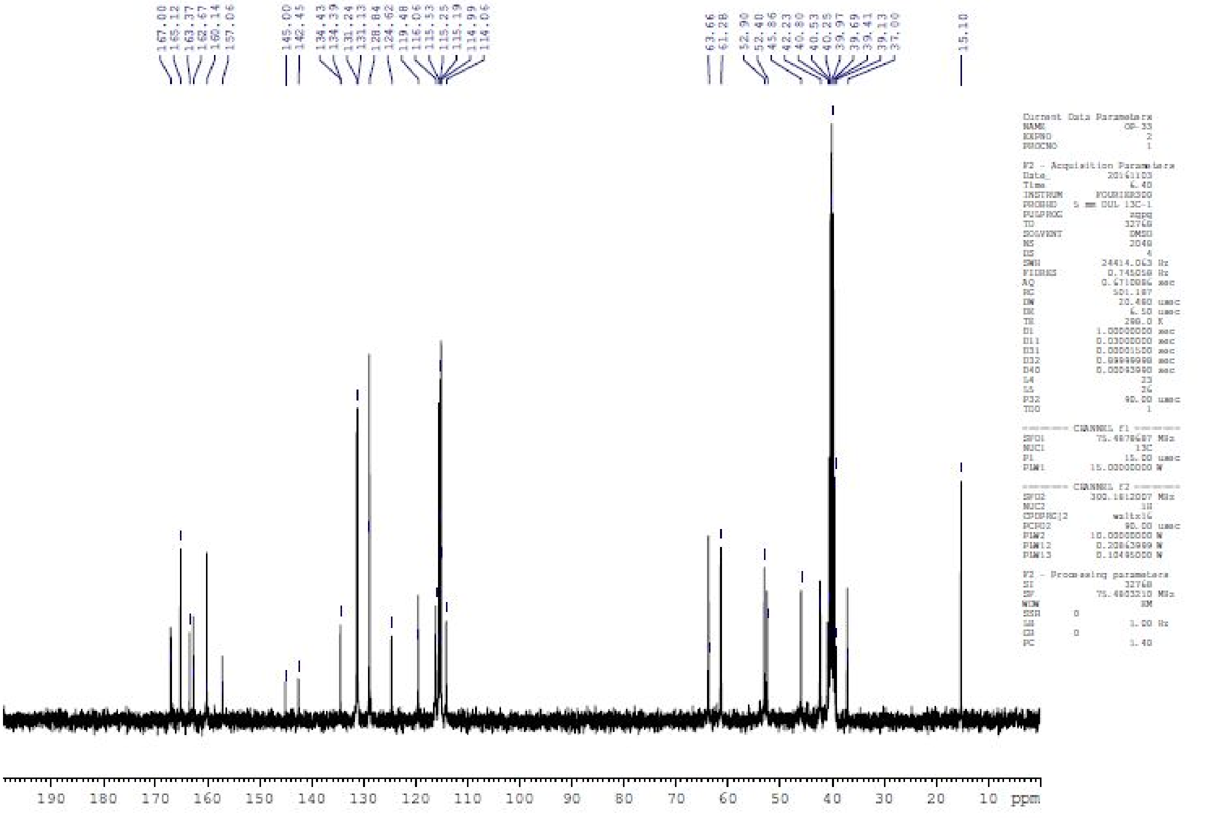

Figure s25. ^{13}C-NMR spectrum of compound **5i**

Data File: C:\LabSolutions\Data\Analiz\uac\OP-33_15.lcd

Elmt	Val.	Min	Max	Elmt	Val.	Min	Max	Elmt	Val.	Min	Max	Elmt	Val.	Min	Max	Use Adduct
H	1	20	40	O	2	1	5	S	2	1	1	Ru	2	0	0	H
C	4	15	30	F	1	0	1	Cl	1	0	1	Pd	2	0	0	
N	3	6	8	P	3	0	0	Br	1	0	1	I	3	0	0	

Error Margin (ppm):	5	DBE Range:	16.0 - 20.0	Electron Ions:	both
HC Ratio:	unlimited	Apply N Rule:	yes	Use MSn Info:	yes
Max Isotopes:	3	Isotope RI (%):	1.00	Isotope Res:	9000
MSn Iso RI (%):	10.00	MSn Logic Mode:	AND	Max Results:	500

Event#: 1 MS(E+) Ret. Time : 4.013 -> 4.213 Scan# : 603 -> 633

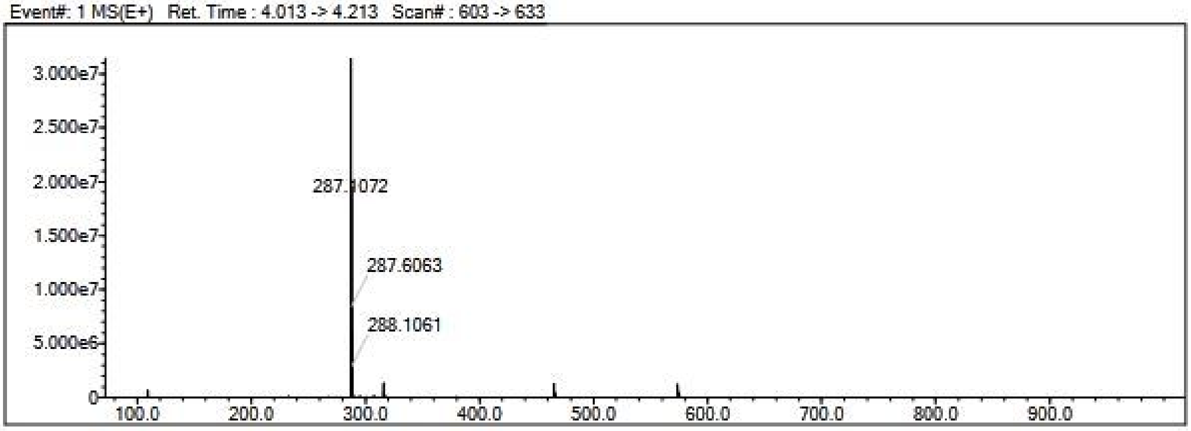

Measured region for 573.2062 m/z

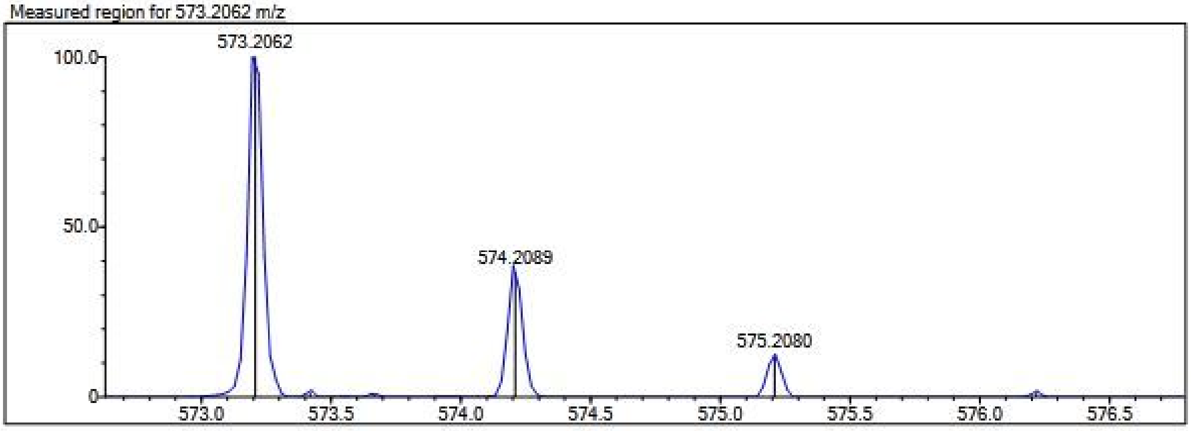

C30 H29 N6 O3 F S [M+H]+ : Predicted region for 573.2079 m/z

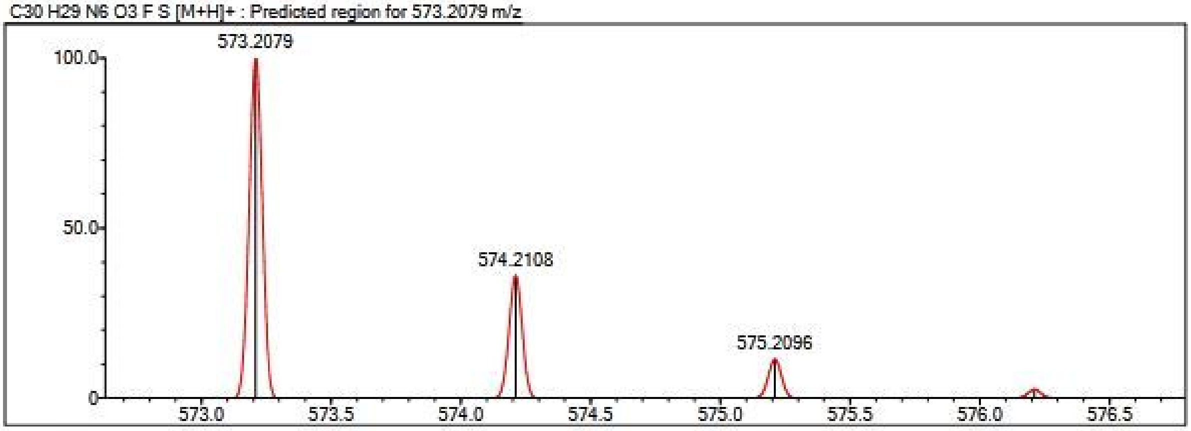

Rank	Score	Formula (M)	Ion	Meas. m/z	Pred. m/z	Df. (mDa)	Df. (ppm)	Iso	DBE
1	88.89	C30 H29 N6 O3 F S	[M+H]+	573.2062	573.2079	-1.7	-2.97	93.50	19.0

Figure s26. Mass spectrum of compound

QSAR methods details

1. Experimental Section

1.1. Molecular Alignment

3D-QSAR studies were performed with Sybyl X-1.2 software installed in a Windows 10 environment on a PC with an Intel core i7 CPU. In order to acquire the best conformers for each molecule, every compound was drawing in ChemDraw and then were subjected to a preliminary geometry optimization using MM2 molecular mechanics. Following, the structures were further minimized by Tripos force field implemented in Sybyl. Merck Molecular Force Field (MMFF94) charges were assigned to each atom. The molecular alignment was done atom-by-atom alignments using a benzimidazole nucleus (**Figure s27**), previously optimized their minima energy conformation from DFT-B3LYP-6-31G(d,p).

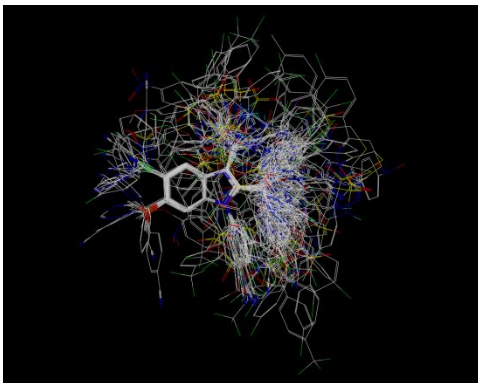

Figure s27. The superimposed structures of all compounds used in the 3D-QSAR model based in atom by atom method.

1.2. CoMFA and CoMSIA field calculation

To derive the CoMFA and CoMSIA descriptor fields, the aligned training set molecules were placed in a three-dimensional cubic lattice with a grid spacing of 2Å in the x, y, and z directions such that the entire set was included in it. The CoMFA steric and electrostatic field energies were calculated using an sp^3 carbon probe atom with a van der Waals radius of 1.52 Å and a charge of +1.0. Cut-off values for both steric and electrostatic fields were set to 30.0 kcal/mol. For CoMSIA analysis, the standard settings (probe with charge +1.0, radius 1Å, hydrophobicity +1.0, H-bond donating +1.0, and H-bond accepting +1.0 [1]) were used to calculate five different fields: steric, electrostatic, hydrophobic, donor, and acceptor. Gaussian-type distance dependence was used to measure the relative attenuation of the field position of each atom in the lattice and led to a much smoother sampling of the fields around the molecules when compared to CoMFA. The default value of 0.3 was set for attenuation factor α.

1.3. Data Set Selection and inhibitory activity

3D-QSAR studies were performed on a set of 169 compounds (**Table S1**). The derivatives displayed aromatase inhibitory activity. The IC_{50} values were converted to pIC_{50} ($-logIC_{50}$). The compounds were randomly divided into training and test sets in a 7:3 ratio. The distribution of pIC_{50} values for the whole set, the training set and the test set is shown in **Figure s28**.

Table s1: Chemical structure of all the compounds used in the 3D-QSAR study

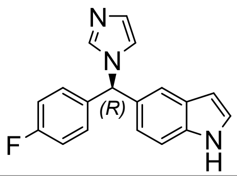	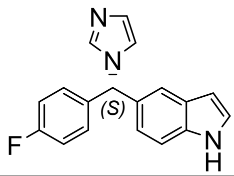	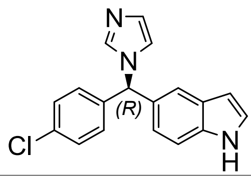

1R [2]	1S [2]	2R [2]
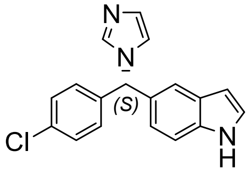	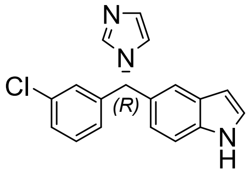	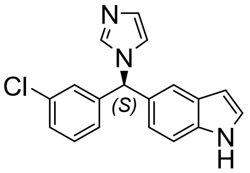
2S [2]	3R [2]	3S [2]
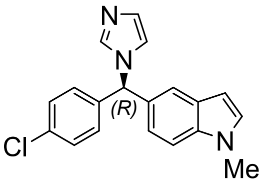	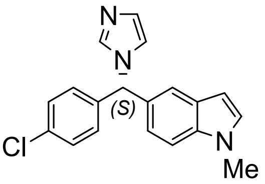	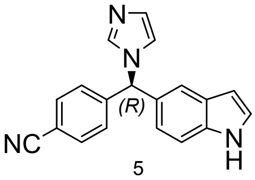
4R [2]	4S [2]	5R [2]
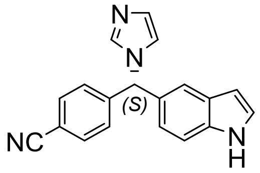	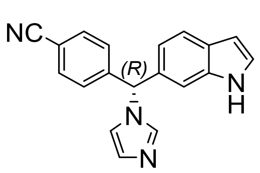	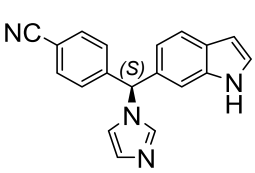
5S [2]	6R [3]	6S [3]
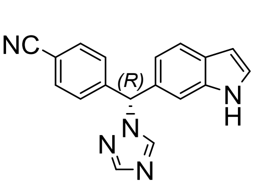	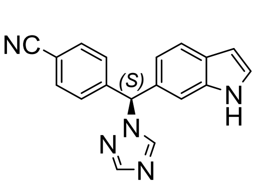	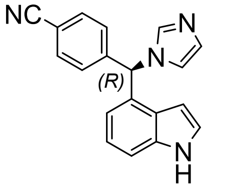
7R [3]	7S [3]	8R [3]
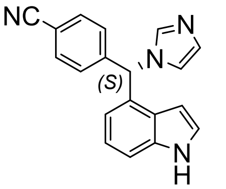	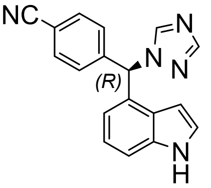	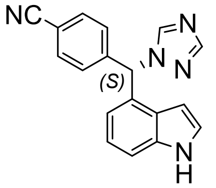
8S [3]	8R [3]	9S [3]
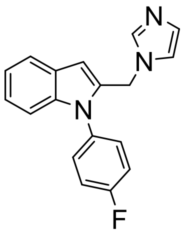	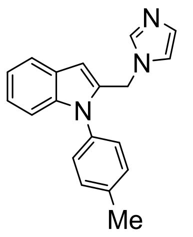	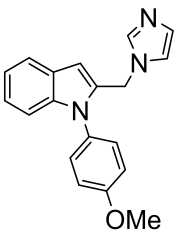
10 [4]	11 [4]	12 [4]
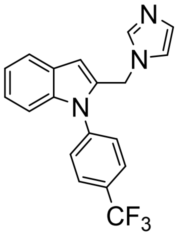	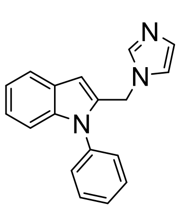	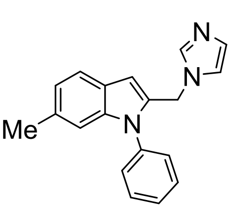
13 [4]	14 [4]	15 [4]

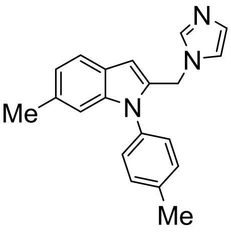

16 [4]

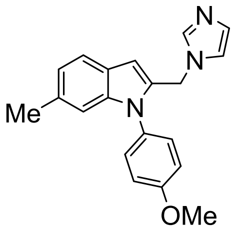

17 [4]

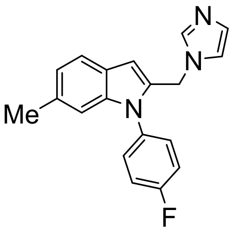

18 [4]

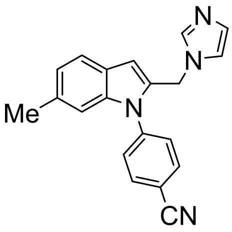

19 [4]

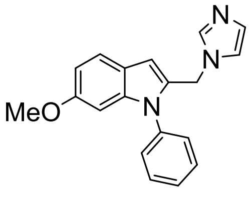

20 [4]

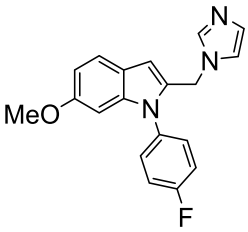

21 [4]

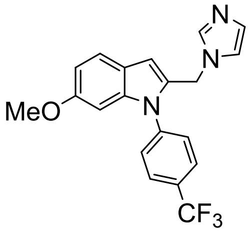

22 [4]

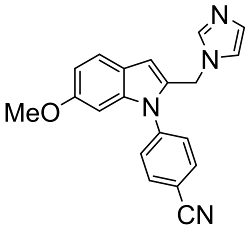

23 [4]

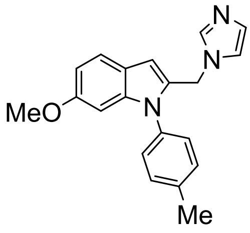

24 [4]

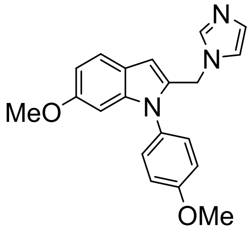

25 [4]

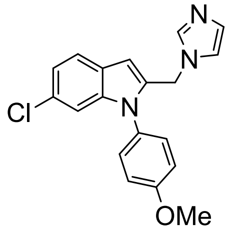

26 [4]

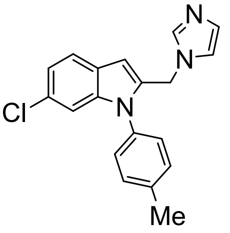

27 [4]

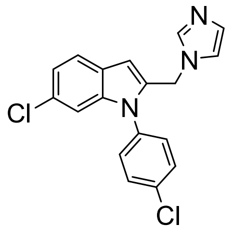

28 [4]

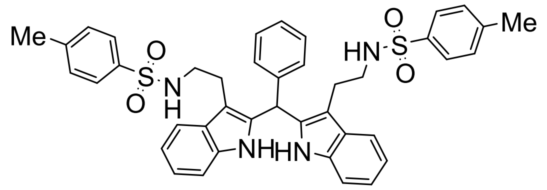

29 [5]

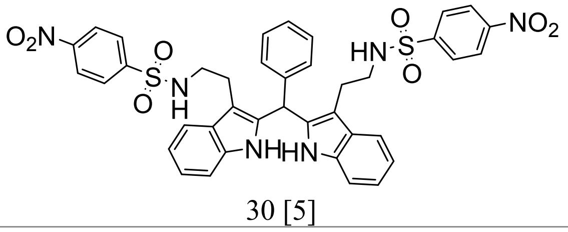

30 [5]

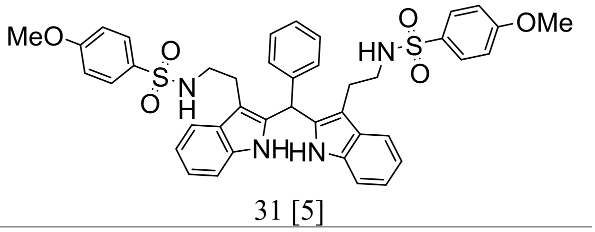

31 [5]

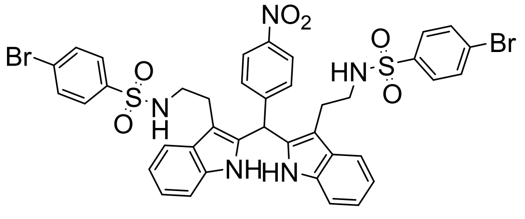

32 [5]

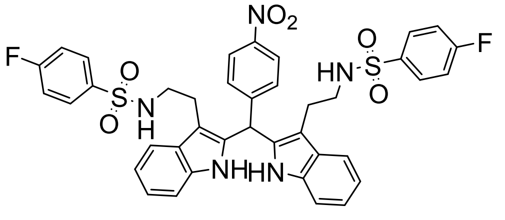

33 [5]

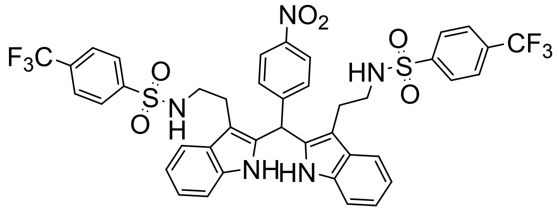

34 [5]

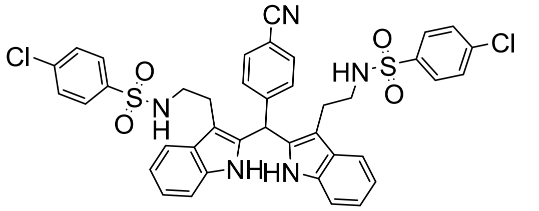

35 [5]

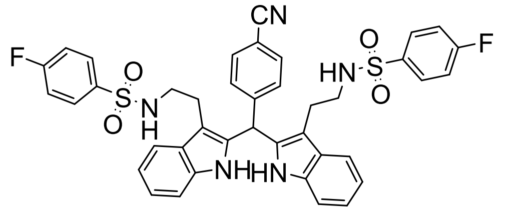

36 [5]

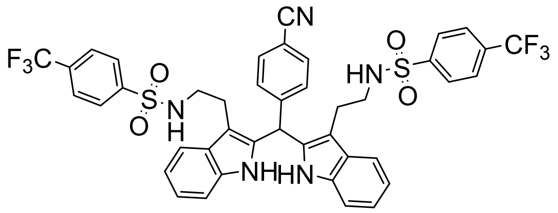

37 [5]

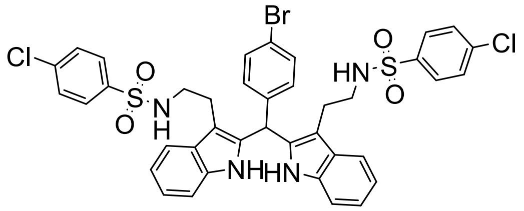

38 [5]

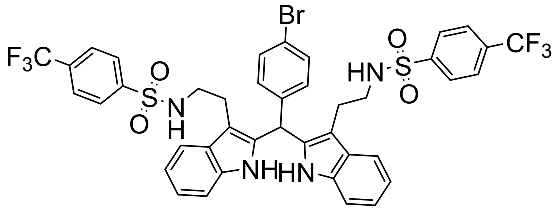

39 [5]

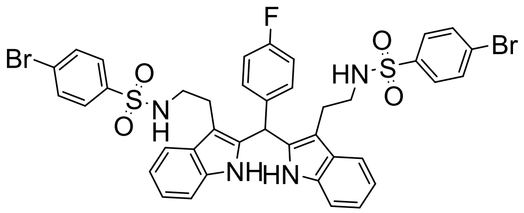

40 [5]

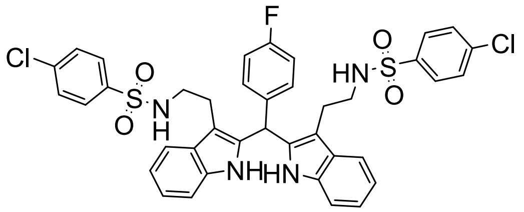

41 [5]

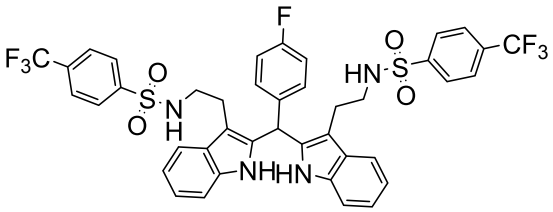

42 [5]

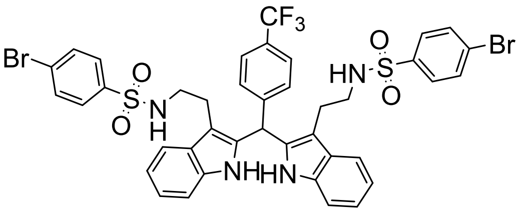

43 [5]

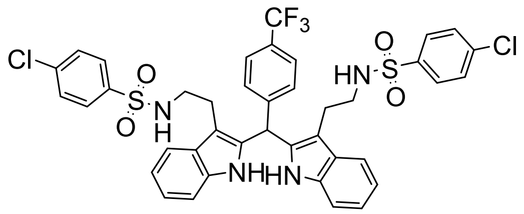

44 [5]

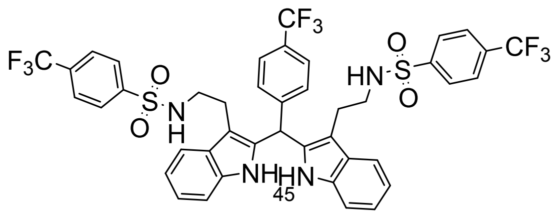

45 [5]

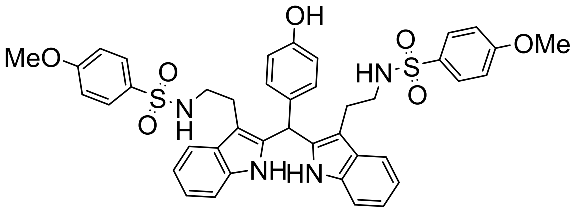

46 [5]

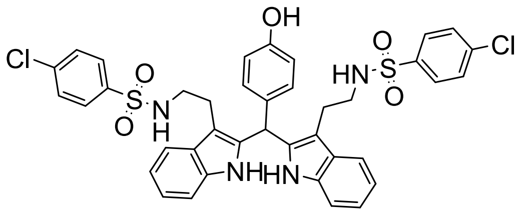

47 [5]

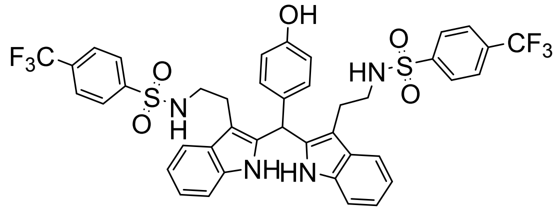

48 [5]

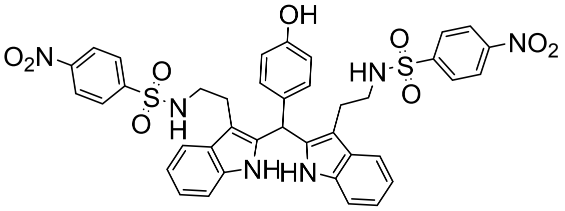

49 [5]

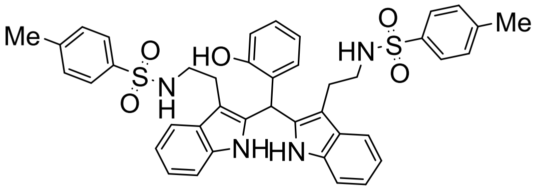

50 [5]

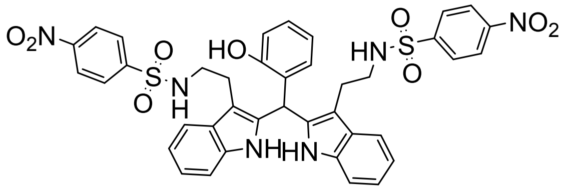

51 [5]

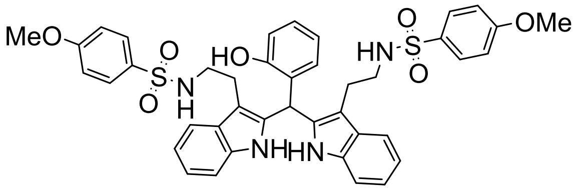

52 [5]

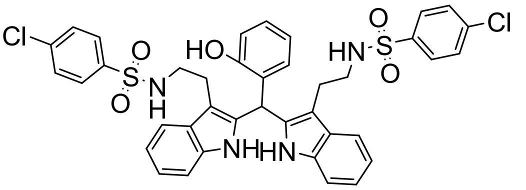

53 [5]

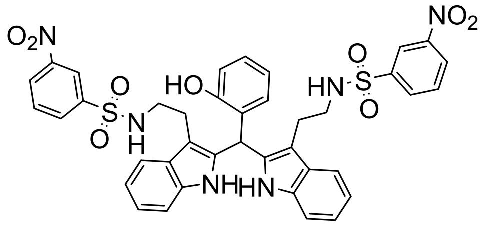

54 [5]

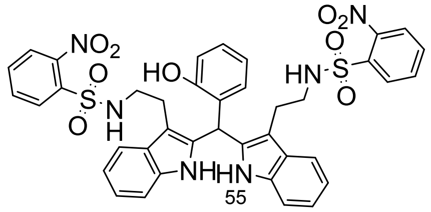

55 [5]

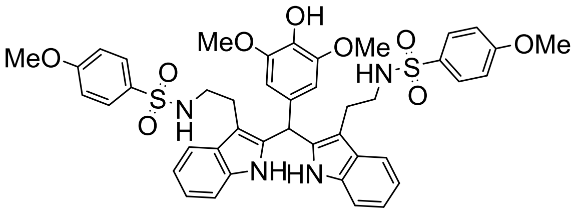

56 [5]

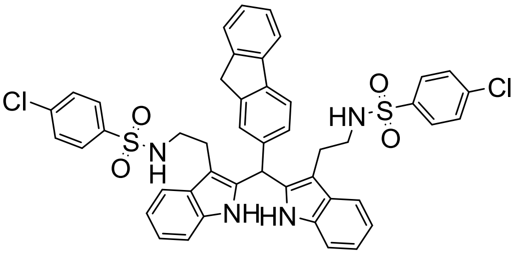

57 [5]

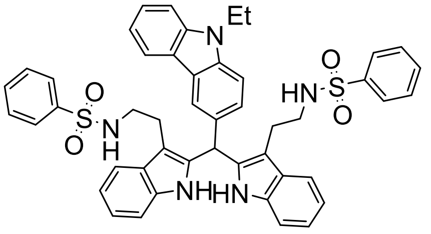

58 [5]

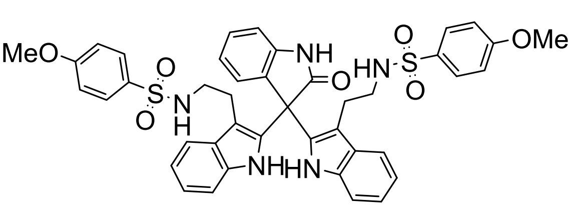

59 [5]

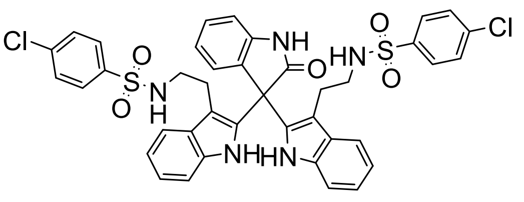

60 [5]

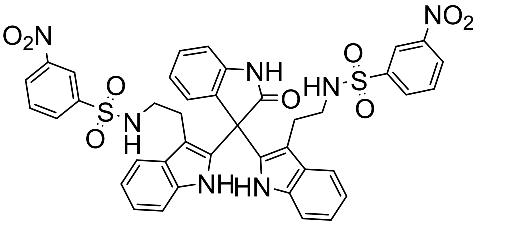

61 [5]

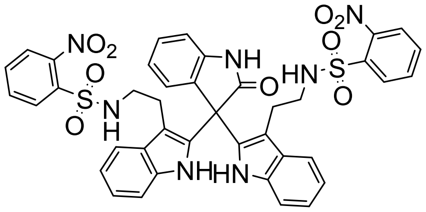

62 [5]

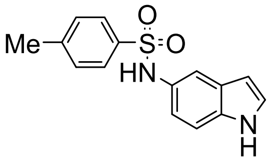

63 [6]

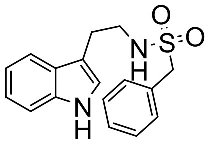

64 [6]

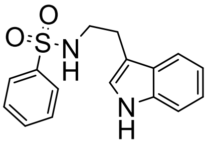

65 [6]

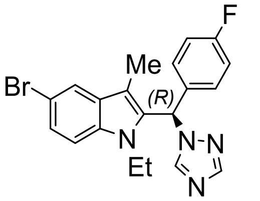

66R [7]

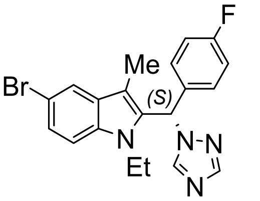

66S [7]

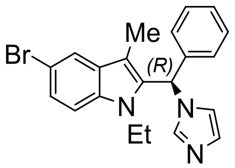

67R [7]

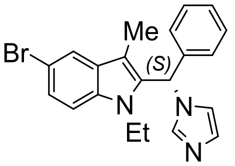

67S [7]

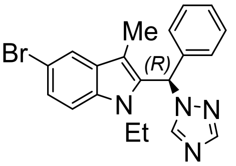

68R [7]

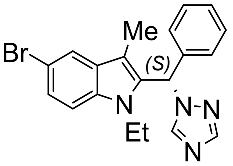

68S [7]

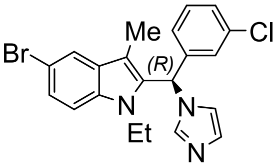

69R [7]

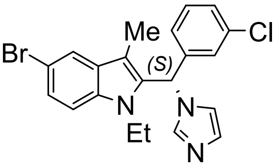

69S [7]

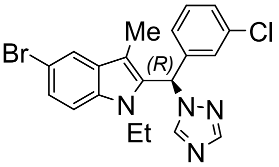

70R [7]

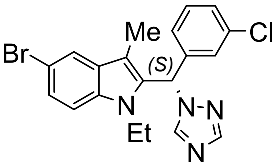

70S [7]

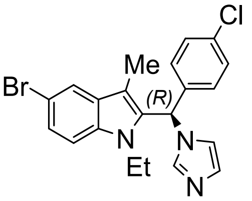

71R [7]

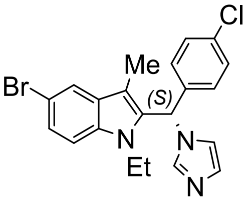

71S [7]

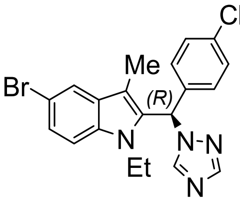

72R [7]

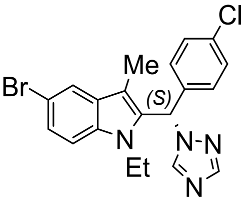

72S [7]

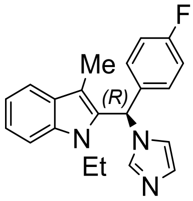

73R [7]

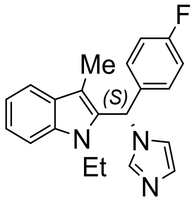

73S [7]

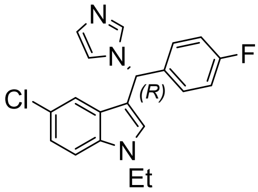

74R [7]

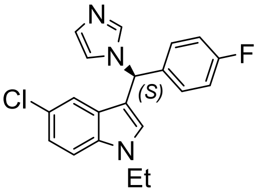

74S [7]

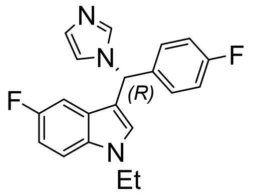
75R [7]

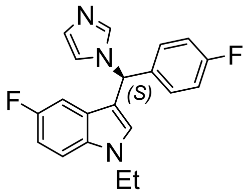
75S [7]

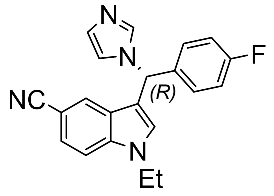
76R [7]

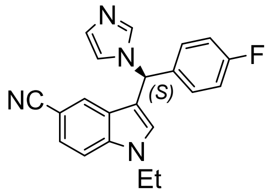
76S [7]

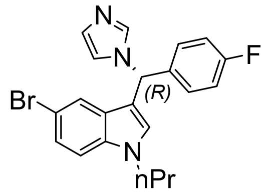
77R [7]

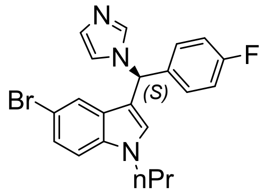
77S [7]

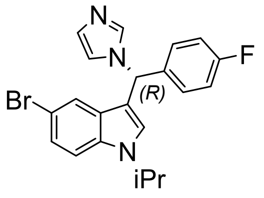
78R [7]

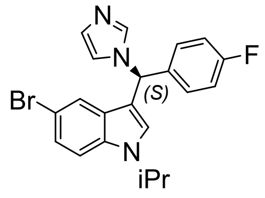
78S [7]

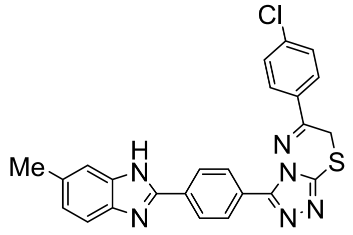
79 [8]

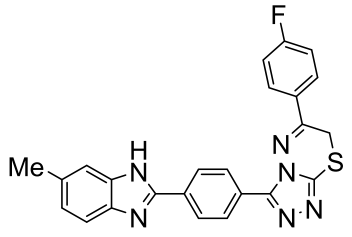
80 [8]

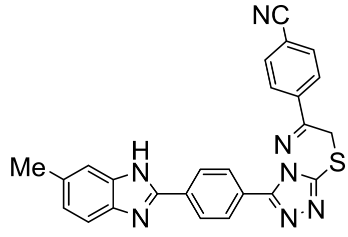
81 [8]

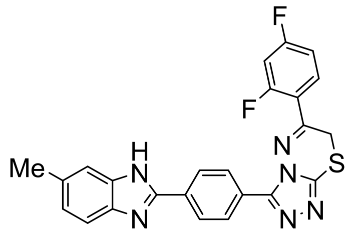
82 [8]

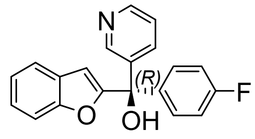
83R [9]

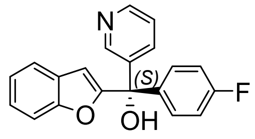
83S [9]

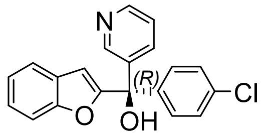
84R [9]

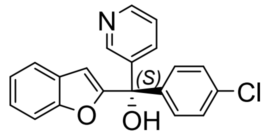
84S [9]

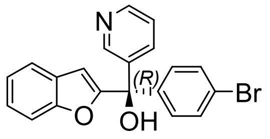
85R [9]

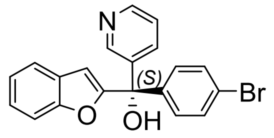
85S [9]

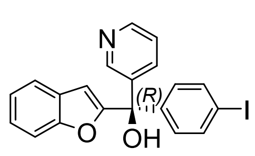
86R [9]

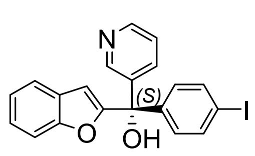
86S [9]

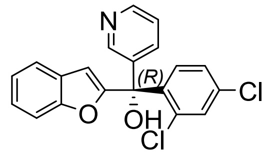
87R [9]

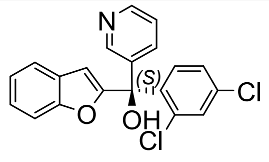
87S [9]

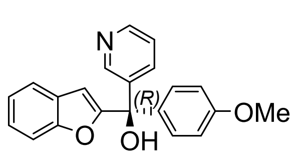
88R [9]

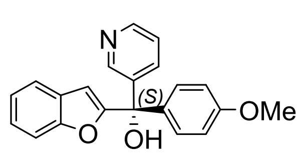
88S [9]

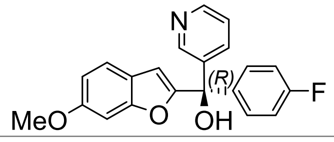

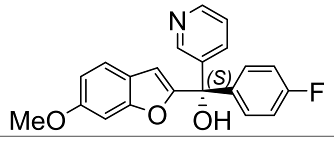

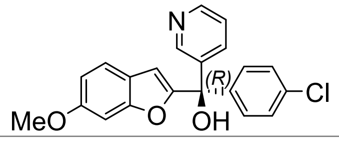

89R [10]	89S [10]	90R [10]
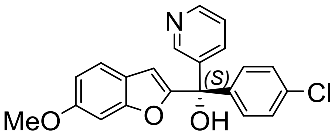	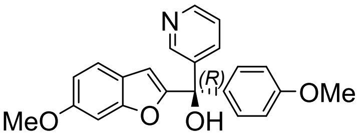	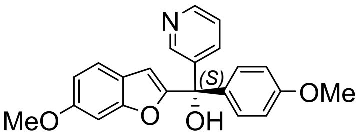
90S [10]	91R [10]	91S [10]
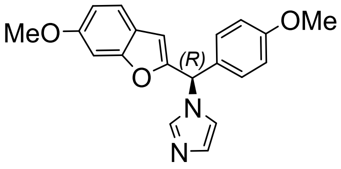	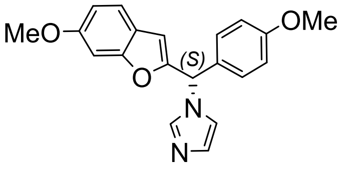	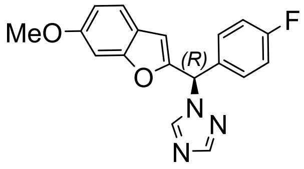
92R [10]	92S [10]	93R [10]
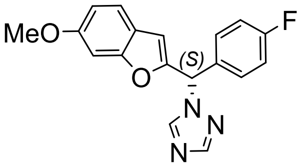	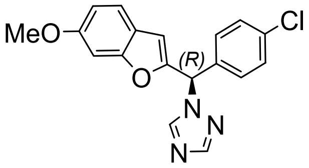	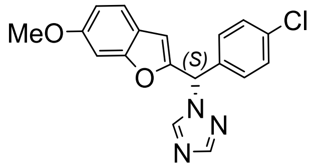
93S [10]	94R [10]	94S [10]
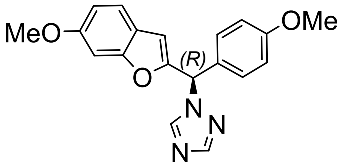	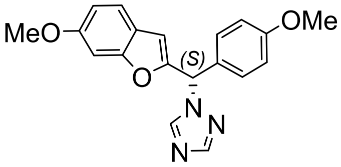	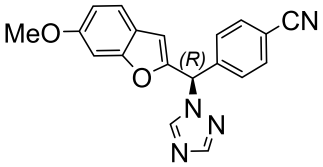
95R [10]	95S [10]	96R [10]
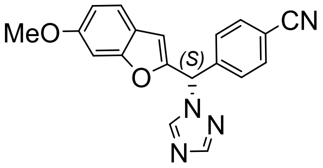	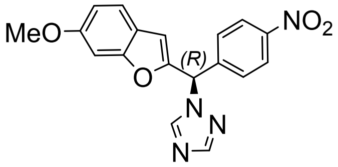	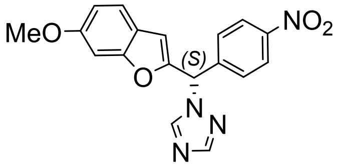
96S [10]	97R [10]	97S [10]
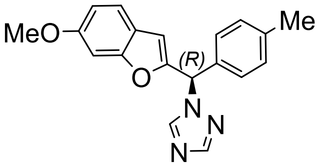	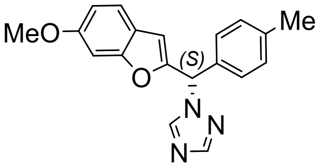	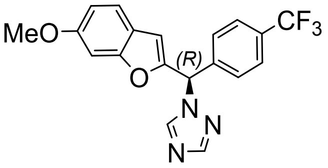
98R [10]	98S [10]	99R [10]
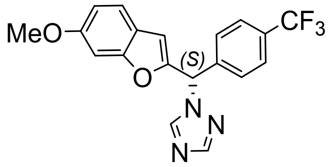	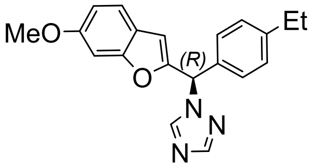	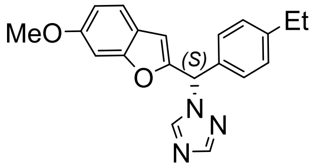
99S [10]	100R [10]	100S [10]

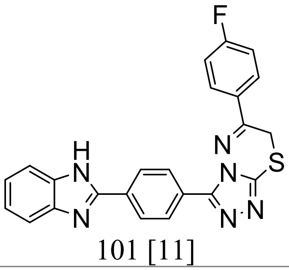	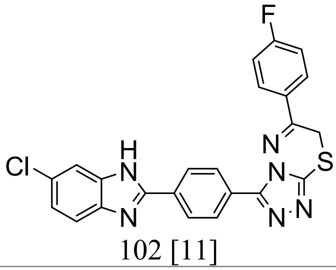	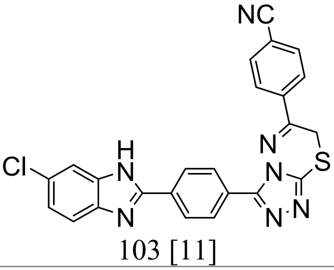
101 [11]	102 [11]	103 [11]

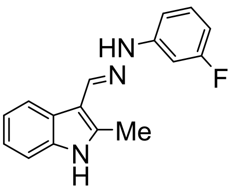 104 [12]	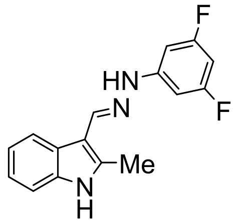 105 [12]	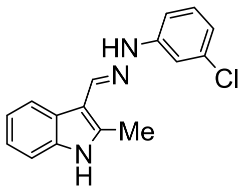 106 [12]
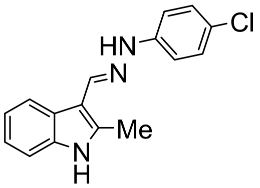 107 [12]	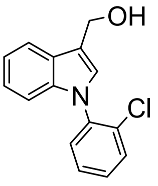 108 [13]	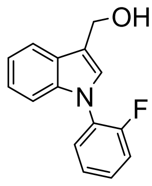 109 [13]
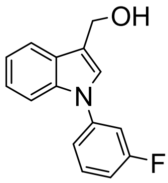 110 [13]	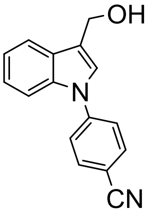 111 [13]	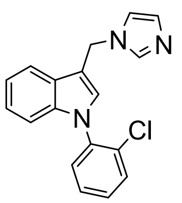 112 [13]
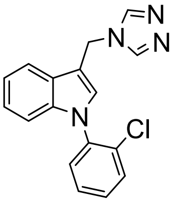 113 [13]	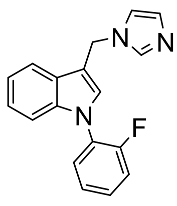 114 [13]	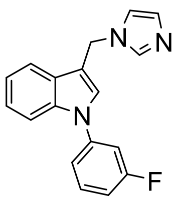 115 [13]
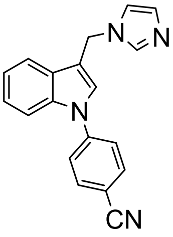 116 [13]	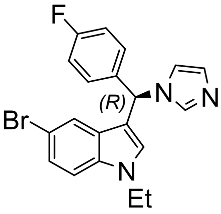 117R [13]	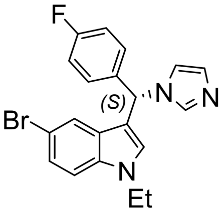 117S [13]
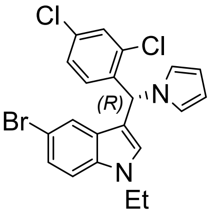 118R [13]	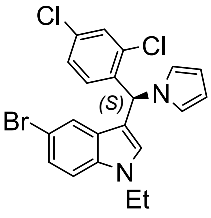 118S [13]	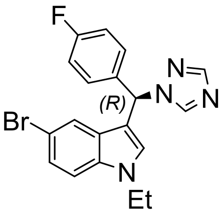 119R [13]
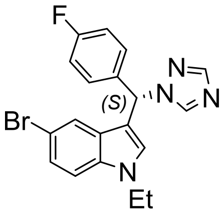 119S [13]	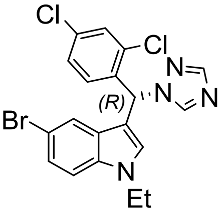 120R [13]	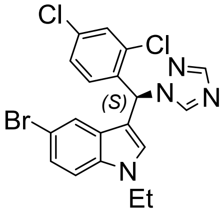 120S [13]

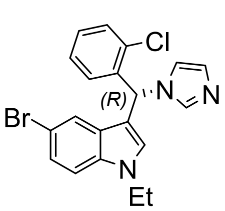 121R [13]	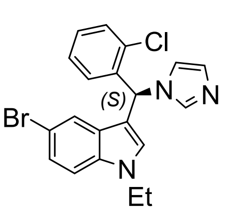 121S [13]	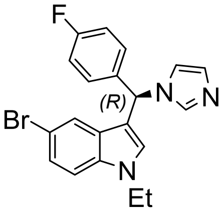 122R [13]
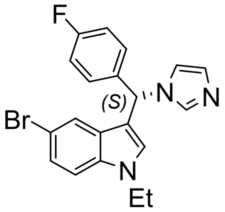 122S [13]	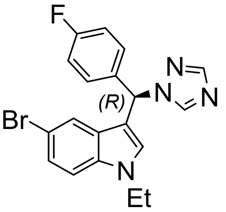 123R [13]	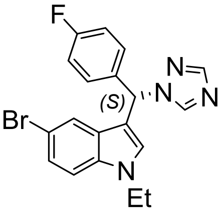 123S [13]

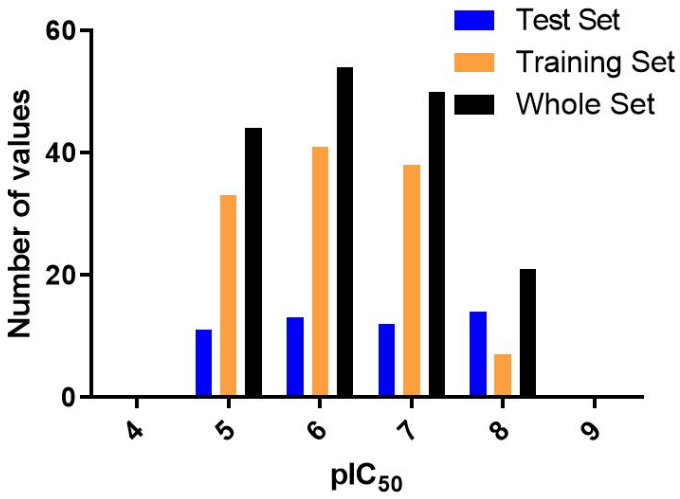

Figure s28. Histogram of frequency distribution data.

1.4. Internal validation and Partial Least Squares (PLS) Analysis

PLS analysis was used to construct a linear correlation between the CoMFA descriptors (independent variables) and the activity values (dependent variables).[14] To select the best model, the cross-validation analysis was performed by using the LOO method (and SAMPLS), which generates the square of the cross-validation coefficient (q^2) and the optimum number of components (N). The non-cross-validation was performed with a column filter value of 2.0 to speed up the analysis and reduce the noise. The q^2, which is a measure of the internal quality of the models, was obtained according to the following equation (1):

$$q^2 = 1 - \frac{\Sigma(y_i - y_{pred})^2}{\Sigma(y_i - \bar{y})^2} \quad (1)$$

Where y_i,$\bar{y}$ and y_{pred} are observed, mean, and predicted activity in the training set, respectively.

1.5. External validation

The models were subjected to external validation criteria according to the proposed test by Golbraikh and Tropsha,[15, 16] which considers a QSAR model predictive, if the following conditions are satisfied:

$$q^2 > 0.5 \quad (2)$$

$$r^2 > 0.6 r_{test}^2 > 0.6 \quad (3)$$

$$\frac{(r^2 - r_0^2)}{r^2} < 0.1 \text{ or } \frac{(r^2 - r_0'^2)}{r^2} < 0.1 \quad (4)$$

$$0.85 \leq k \leq 1.15 \text{ or } 0.85 \leq k' \leq 1.15 \quad (5)$$

It has been demonstrated that all of the above criteria are indeed necessary to adequately assess the predictive ability of a QSAR model.

Furthermore, the external predictive power of the developed 3D-QSAR models using the test set was examined by considering r_m^2 metrics as shown below: [17]

$$r_m^2 = r^2 \left(1 - \left|\sqrt{r^2 - r_0^2}\right|\right) \quad (6)$$

Where r^2 and r_0^2 are squared correlation coefficients between the observed and predicted activities of the test set with and without intercept, respectively. For a significant external model validation, the value of r_m^2 should be greater than 0.5.

Additionally, the following descriptors were calculated:

$$Q_{F1}^2 = 1 - \frac{\sum_{i=1}^{n_{EXT}} (y_i - \hat{y}_i)^2}{\sum_{i=1}^{n_{EXT}} (y_i - \bar{y}_{TR})^2} \quad (7)$$

$$Q_{F2}^2 = 1 - \frac{\sum_{i=1}^{n_{EXT}} (y_i - \hat{y}_i)^2}{\sum_{i=1}^{n_{EXT}} (y_i - \bar{y}_{EXT})^2} \quad (8)$$

$$CCC = \frac{2\sum_{i=1}^{n_{EXT}} (y_i - \bar{y})(\hat{y}_i - \bar{\hat{y}})}{\sum_{i=1}^{n_{EXT}} (y_i - \bar{y})^2 + \sum_{i=1}^{n_{EXT}} (\hat{y}_i - \bar{\hat{y}})^2 + n_{EXT}(\bar{y} - \bar{\hat{y}})^2} \quad (9)$$

$$\Delta r_m^2 = \left|r_m^2 - r_m'^2\right| \quad (10)$$

Where, TR = training set, EXT = external prediction set, y_i= experimental data values, $\hat{y}_i$=predicted data values, $\bar{y}$=average of the experimental data values, $\bar{\hat{y}}$=average of the predicted data values. Finally, r_m^2 is calculated using the experimental values on the ordinate axis, while $r_m'^2$ using them on the abscissa.

1.6. Applicability domain calculation

The AD was evaluated based on the simple standardization method reported by Roy *et al.*[18] First, each descriptor "i" for each compound "k" is standardized (S_{ik}). Every compound must have a maximum value $[S_i]_{max(k)} \leq 3$. In the case that $[S_i]_{max(k)} > 3$ and its minimum value $[s_i]_{min(k)} < 3$, then the $S_{new(k)}$ parameter must be calculated and has to fulfill the condition: $S_{new(k)} = \bar{S}_k + 1.28 * \sigma_{S_k}$, where $\bar{S}_k$ is the mean of Sik values for compound k and σ_{S_k} is the standard deviation for such values. The software is available free of charge on the authors' website: http://dtclab.webs.com/software-tools and http://teqip.jdvu.ac.in/QSAR_Tools/.

Table s2: Combination fields and statistical values of CoMFA model on aromatase inhibitor.

Field Comb.	q^2	N	SEP	SEE	r^2_{ncv}	F	Field Contribution	
							S	E
**S**	0.838	4	0.394	0.25	0.935	408.4	1	
**E**	-0.017	1	0.975	0.948	0.039	4.7		1
**SE**	0.842	7	0.4	0.214	0.953	324.6	0.871	0.129

q^2, the square of the LOO cross-validation (CV) coefficient; N, the optimum number of components; SEP, standard error of prediction; SEE, standard error of estimation of non CV analysis; r^2_{ncv}, square of the non CV coefficient; F, F-test value; S and E are the steric and electrostatic contributions respectively.

Table s3: Combination fields and statistical values of CoMFA model on aromatase inhibitor.

Field Comb.	q^2	N	SEP	SEE	r^2_{ncv}	F	Field Contribution				
							S	E	H	D	A
**S**	0.814	4	0.422	0.309	0.901	258.4	1				
**E**	-0.035	1	0.983	0.939	0.056	6.7		1			
**H**	0.737	8	0.511	0.181	0.967	403.9			1		
**D**	0.312	3	0.808	0.695	0.492	37.1				1	
**A**	0.332	2	0.794	0.729	0.436	44.9					1
**SE**	0.818	8	0.426	0.283	0.919	157.1	0.893	0.107			
**SEH**	0.833	7	0.406	0.218	0.952	312.4	0.403	0.082	0.515		
**SEHD**	0.817	5	0.421	0.931	0.258	305.7	0.29	0.06	0.399	0.252	
**SEHA**	0.81	11	0.441	0.141	0.98	486.9	0.309	0.039	0.437		0.215
**SED**	0.802	9	0.446	0.25	0.938	183	0.614	0.067		0.319	
**SEA**	0.795	17	0.471	0.141	0.982	317.5	0.591	0.048			0.361
**SEDA**	0.785	7	0.46	0.281	0.92	181.5	0.481	0.07		0.23	0.219
**SH**	0.836	6	0.401	0.179	0.967	549.9	0.428		0.572		
**SD**	0.808	6	0.445	0.259	0.931	251.9	0.632			0.368	
**SA**	0.816	12	0.436	0.159	0.976	351.9	0.612				0.388
**SHD**	0.806	3	0.429	0.319	0.893	319	0.315		0.415	0.269	
**SHA**	0.817	8	0.426	0.15	0.977	592.7	0.314		0.453		0.232
**SDA**	0.789	5	0.452	0.311	0.9	203.9	0.498			0.261	0.241
**SHDA**	0.789	8	0.458	0.166	0.972	480.9	0.274		0.393	0.163	0.169
**EH**	0.722	5	0.519	0.343	0.879	163.7		0.105	0.895		
**ED**	0.294	3	0.819	0.713	0.465	33.3		0.171		0.829	
**EA**	0.33	3	0.798	0.712	0.466	33.5		0.184			0.816
**EHD**	0.735	5	0.506	0.322	0.893	188.4		0.086	0.575	0.339	
**EHA**	0.691	12	0.564	0.163	0.974	332.8		0.042	0.653		0.305
**EDA**	0.34	2	0.789	0.475	0.703	52.5		0.184		0.376	0.439
**EHDA**	0.688	5	0.55	0.328	0.889	181		0.077	0.479	0.231	0.213
**HD**	0.726	3	0.511	0.357	0.866	247.5			0.599	0.401	
**HA**	0.712	9	0.538	0.167	0.972	425.8			0.68		0.32

**HDA**	0.692	4	0.543	0.344	0.876	202.2			0.51	0.247	0.243
**DA**	0.336	3	0.794	0.654	0.55	46.8				0.481	0.519
**ALL**	0.783	5	0.459	0.271	0.924	274	0.243	0.064	0.356	0.191	0.146

q^2, the square of the LOO cross-validation (CV) coefficient; N, the optimum number of components; SEP, standard error of prediction; SEE, standard error of estimation of non CV analysis; r^2_{ncv}, square of the non CV coefficient; F, F-test value; S, E, H, D and A are the steric, electrostatic, hydrophobic, hydrogen-bond donor, and hydrogen-bond acceptor contributions respectively.

Table s4: Summary of CoMFA and CoMSIA Models of aromatase inhibitors and their r^2 predictive values.

**Model**	**q^2**	**N**	**SEP**	**SEE**	**r^2_{ncv}**	**F**	**PRESS**	**SD**	**r^2_{Pred}**
CoMFA (SE)	0.814	4	0.422	0.309	0.901	258.4	6.5688	26.7509	0.7544
CoMSIA (SH)	0.836	6	0.401	0.179	0.967	549.9	7.1562	30.3186	0.7640

q^2, the square of the LOO cross-validation (CV) coefficient; N, the optimum number of components; SEP, standard error of prediction; SEE, standard error of estimation of non CV analysis; r^2_{ncv}, square of the non CV coefficient; F, F-test value; PRESS= the sum of the squared deviations between predicted and actual biological activity values for every molecule in the test set; SD= is the sum of the squared deviation between the biological activity of molecules in the test set and the mean activity of the training set molecules; r^2_{Pred}= is the predictive r^2 based only on the test set molecules.

Table s5: Experimental pIC_{50}, predicted pIC_{50} and residual values for the analyzed compounds obtained with the CoMFA and CoMSIA models.

Compound	Expetimental pIC_{50}	CoMFA		CoMSIA	
		pIC_{50}	Δ	pIC_{50}	Δ
**1R**	7.580	7.495	0.085	7.518	0.062
**1S**	7.580	7.520	0.060	7.649	-0.069
**2R**[a,b]	7.815	7.601	0.214	7.609	0.206
**2S**	7.815	7.498	0.317	7.683	0.132
**3R**	6.527	6.589	-0.062	6.404	0.123
**3S**	6.527	6.571	-0.044	6.363	0.164
**4R**[a,b]	7.625	7.528	0.097	7.688	-0.062
**4S**	7.625	7.557	0.068	7.704	-0.079
**5R**	7.714	7.553	0.161	7.640	0.074
**5S**	7.714	7.318	0.396	7.569	0.145
**6R**	7.939	7.893	0.046	7.842	0.097
**6S**	7.939	7.891	0.048	7.850	0.089
**7R**	7.028	7.301	-0.273	7.100	-0.072
**7S**[a,b]	7.028	7.433	-0.406	7.236	-0.209
**8R**[a,b]	7.728	6.878	0.850	7.060	0.668
**8S**[a,b]	7.728	6.938	0.790	7.106	0.622
**9R**[a,b]	5.790	6.466	-0.676	6.724	-0.934
**9S**[a,b]	5.790	6.476	-0.685	6.637	-0.846
**10**	8.045	7.636	0.409	7.725	0.320
**11**	6.827	6.850	-0.023	6.685	0.142
**12**	7.111	7.334	-0.223	7.367	-0.255
**13**	8.307	7.377	0.930	7.669	0.638
**14**[a,b]	7.780	7.520	0.260	7.655	0.125
**15**[a,b]	7.670	7.580	0.090	7.719	-0.049
**16**[a,b]	6.785	6.343	0.442	6.623	0.162
**17**[a,b]	6.858	6.939	-0.081	6.989	-0.132
**18**[a,b]	7.245	7.021	0.225	7.418	-0.172
**19**	7.568	7.737	-0.169	7.474	0.095
**20**	8.206	8.095	0.111	7.926	0.280
**21**	7.310	7.565	-0.255	7.461	-0.151
**22**	7.592	7.865	-0.273	7.843	-0.251
**23**	7.329	7.640	-0.311	7.374	-0.045
**24**	7.241	7.552	-0.311	7.599	-0.358
**25**	6.954	7.147	-0.193	7.020	-0.066
**26**	6.692	6.923	-0.231	6.638	0.054
**27**	6.628	6.831	-0.203	6.830	-0.202
**28**	6.663	6.761	-0.098	6.976	-0.313
**29**	4.924	4.781	0.143	4.979	-0.054
**30**	4.951	5.151	-0.200	5.017	-0.066
**31**[a,b]	5.456	5.417	0.039	5.380	0.076
**32**[a,b]	5.569	5.518	0.051	5.279	0.290
**34**	5.398	5.250	0.148	5.326	0.072

**35**[a,b]	5.260	5.245	0.014	5.348	-0.089
**36**	5.009	4.978	0.031	5.027	-0.018
**37**[a,b]	4.815	4.891	-0.076	5.444	-0.628
**38**	5.569	5.417	0.152	5.587	-0.018
**39**	5.469	5.318	0.151	5.455	0.014
**40**	5.119	5.183	-0.064	5.055	0.064
**41**[a,b]	5.268	5.296	-0.029	5.309	-0.042
**42**	5.357	5.378	-0.021	5.456	-0.099
**43**	5.420	5.296	0.124	5.357	0.063
**44**	5.495	5.570	-0.075	5.606	-0.111
**45**	5.638	5.511	0.127	5.653	-0.015
**46**	6.155	5.973	0.182	6.193	-0.038
**47**	5.854	5.941	-0.087	6.108	-0.254
**48**	5.886	5.800	0.086	5.808	0.078
**49**	5.638	5.584	0.054	5.573	0.065
**50**	5.387	5.296	0.091	5.227	0.160
**51**	5.553	5.312	0.241	5.546	0.007
**52**[a,b]	6.097	5.809	0.287	6.232	-0.135
**53**	5.377	5.375	0.002	5.263	0.114
**54**[a,b]	5.770	5.749	0.020	5.689	0.080
**55**[a,b]	5.337	5.333	0.004	5.377	-0.040
**56**	5.602	5.566	0.036	5.481	0.121
**57**	5.056	5.156	-0.100	4.735	0.321
**58**[a,b]	5.409	5.528	-0.119	5.321	0.088
**59**	5.553	5.513	0.040	5.595	-0.042
**60**[a,b]	5.081	5.087	-0.006	5.296	-0.215
**61**[a,b]	4.924	4.768	0.157	5.037	-0.112
**62**	5.009	5.083	-0.074	5.229	-0.220
**63**	6.796	6.960	-0.164	6.883	-0.087
**64**	6.125	5.935	0.190	6.243	-0.118
**65**	6.699	6.412	0.287	6.719	-0.020
**66R**	5.461	5.219	0.242	5.188	0.273
**66S**[a,b]	5.461	5.451	0.010	5.464	-0.003
**67R**	6.377	6.311	0.066	6.334	0.043
**67S**	6.377	6.577	-0.200	6.631	-0.254
**68R**	5.485	5.260	0.225	5.352	0.134
**68S**[a,b]	5.485	5.549	-0.064	5.719	-0.234
**69R**[a,b]	6.027	6.141	-0.115	6.021	0.006
**69S**[a,b]	6.027	5.993	0.033	6.054	-0.027
**70R**	4.757	4.838	-0.081	4.679	0.078
**70S**	4.757	4.830	-0.073	4.731	0.026
**71R**	6.387	6.604	-0.217	6.640	-0.253
**71S**[a,b]	6.387	6.555	-0.168	6.232	0.155
**72R**[a,b]	5.726	5.558	0.168	5.537	0.189
**72S**	5.726	5.914	-0.188	5.928	-0.202
**73R**	6.921	6.680	0.241	6.778	0.143

**73S**[a,b]	6.921	6.187	0.734	6.921	0.000
**74R**	6.824	6.728	0.096	6.866	-0.042
**74S**	6.824	6.728	0.096	6.866	-0.042
**75R**	7.222	7.213	0.009	7.170	0.052
**75S**	7.222	7.213	0.009	7.170	0.052
**76R**	6.495	6.709	-0.214	6.658	-0.163
**76S**	6.495	6.675	-0.180	6.655	-0.160
**77R**[a,b]	6.469	6.035	0.433	6.139	0.330
**77S**[a,b]	6.469	6.084	0.384	6.139	0.330
**78R**[a,b]	6.495	6.523	-0.028	6.444	0.051
**78S**[a,b]	6.495	6.523	-0.028	6.444	0.051
**79**	5.927	5.949	-0.022	6.149	-0.222
**80**	5.906	6.158	-0.252	6.129	-0.223
**81**[a,b]	5.871	5.946	-0.075	5.927	-0.056
**82**[a,b]	7.432	6.826	0.606	6.867	0.565
**83R**	5.155	5.351	-0.196	4.983	0.172
**83S**	5.155	5.198	-0.043	5.292	-0.137
**84R**	5.367	5.256	0.111	5.217	0.150
**84S**	5.367	5.480	-0.113	5.534	-0.167
**85R**	4.600	4.931	-0.331	4.692	-0.092
**85S**	4.600	4.534	0.066	4.619	-0.019
**86R**	4.893	4.611	0.282	4.814	0.079
**86S**	4.893	4.835	0.058	4.761	0.132
**87R**[a,b]	5.658	5.858	-0.200	5.755	-0.097
**87S**	5.658	5.603	0.055	5.486	0.172
**88R**	5.886	6.052	-0.166	5.898	-0.012
**88S**	5.886	5.952	-0.066	6.079	-0.193
**89R**	7.357	7.218	0.139	7.361	-0.004
**89S**[a,b]	7.357	7.119	0.237	7.359	-0.002
**90R**	7.310	7.216	0.094	7.373	-0.063
**90S**	7.310	7.488	-0.178	7.343	-0.033
**91R**	6.796	6.934	-0.138	7.050	-0.254
**91S**[a,b]	6.796	6.807	-0.011	6.780	0.016
**92R**	6.886	7.187	-0.301	7.138	-0.252
**92S**	6.886	7.036	-0.150	6.913	-0.027
**93R**	7.310	7.202	0.108	7.231	0.079
**93S**	7.310	7.328	-0.018	7.182	0.128
**94R**[a,b]	7.357	7.159	0.197	7.383	-0.026
**94S**	7.357	7.556	-0.199	7.386	-0.029
**95R**	6.886	7.179	-0.293	7.158	-0.272
**95S**	6.886	7.151	-0.265	6.939	-0.053
**96R**	8.000	7.524	0.476	7.555	0.445
**96S**	8.000	7.298	0.702	7.340	0.660
**97R**	6.222	6.106	0.116	6.340	-0.118
**97S**	6.222	6.489	-0.267	6.130	0.092
**98R**	7.000	7.176	-0.176	6.946	0.054

**98S**	7.000	7.054	-0.054	6.965	0.035
**99R**	6.886	7.042	-0.156	7.049	-0.163
**99S**[a,b]	6.886	6.853	0.033	6.900	-0.014
**100R**	5.910	5.828	0.082	5.778	0.132
**100S**	5.910	5.993	-0.083	5.906	0.004
**101**	5.765	5.657	0.108	5.735	0.031
**102**	5.643	5.754	-0.111	5.704	-0.061
**103**	5.806	6.140	-0.334	5.895	-0.089
**104**	4.939	5.139	-0.200	5.144	-0.205
**105**	4.886	4.991	-0.105	4.929	-0.043
**106**	4.718	4.910	-0.192	4.764	-0.046
**107**	5.062	5.128	-0.066	4.874	0.188
**108**	7.268	7.013	0.255	7.153	0.115
**109**	6.907	6.734	0.173	6.952	-0.045
**110**	7.086	7.031	0.055	7.263	-0.177
**111**	7.301	6.968	0.333	6.961	0.340
**112**	6.762	6.887	-0.125	6.867	-0.105
**113**[a,b]	5.456	6.108	-0.652	6.394	-0.938
**114**[a,b]	6.377	6.342	0.035	6.400	-0.023
**115**[a,b]	5.513	5.878	-0.365	6.208	-0.696
**116**	5.851	5.882	-0.031	6.099	-0.248
**117R**[a,b]	7.284	6.684	0.600	6.624	0.660
**117S**	7.284	7.195	0.089	7.126	0.158
**118R**	6.478	6.501	-0.023	6.451	0.027
**118S**[a,b]	6.478	6.084	0.394	5.810	0.668
**119R**[a,b]	6.016	5.900	0.116	6.226	-0.210
**119S**	6.016	5.695	0.321	5.795	0.221
**120R**	6.206	6.352	-0.146	6.414	-0.208
**120S**	6.206	6.193	0.013	6.324	-0.118
**121R**	6.009	5.888	0.121	5.948	0.061
**121S**	6.009	5.934	0.075	5.881	0.128
**122R**[a,b]	5.357	5.587	-0.231	5.399	-0.042
**122S**	5.357	5.477	-0.120	5.438	-0.081
**123R**[a,b]	5.149	5.699	-0.550	5.607	-0.459
**123S**[a,b]	5.149	5.993	-0.844	5.424	-0.275

[a]= Test Set compound used in CoMFA model of aromatase inhibitors. [b]= Test Set compound used in CoMSIA model of aromatase inhibitors. OUT= Outliers compound.

Table s6: External validation

Condition	Parameter	Threshold value	CoMFA	CoMSIA
**1**	q^2	>0.5	0.842	0.836
**2**	r^2	>0.6	0.845	0.836
**3a**	r_0^2	Close to value of r^2	0.997	0.997
**3b**	$r_0'^2$	Close to value of r^2	0.997	0.997
**4a**	k	$0.85 < k < 1.15$	0.992	1.001
**4b**	k'	$0.85 < k' < 1.15$	1.005	0.996
**5a**	$\lvert(r^2 - r_0^2)\rvert/r^2$	<0.1	0.180	0.193
**5b**	$\lvert(r^2 - r_0'^2)\rvert/r^2$	<0.1	0.180	0.193
**6**	$\lvert r_0^2 - r_0'^2\rvert$	<0.3	0.000	0.000
**7**	r_m^2	>0.5	0.516	0.501
**8**	Δr^2_m	<0.2	0.000	0.000
**9**	Q^2_{F1}	>0.7	0.842	0.835
**10**	Q^2_{F2}	>0.7	0.840	0.834
**11**	CCC	>0.85	0.907	0.905

q^2 = the square of the LOO cross-validation (CV) coefficient; r^2 is the regression coefficient for the test set exclusively; r_0^2 and k are the correlation coefficient between the experimental and predicted activities for test set and the respective slope of regression; and $r_0'^2$ and k' are the correlation coefficient between the predicted and experimental activities for test set and the respective slope of regression. r^2_mcorresponds to "r^2 metrics" for test set. Parameters 7-11 are defined in the methods section.

Table s7: *Y*-random test

	CoMFA-SE		CoMSIA-SH	
	q^2	r_{ncv}^2	q^2	r_{ncv}^2
**Random_1**	0.012	0.172	-0.093	0.388
**Random_2**	-0.071	0.130	-0.158	0.194
**Random_3**	0.002	0.166	-0.011	0.523
**Random_4**	-0.038	0.142	-0.225	0.194
**Random_5**	-0.046	0.150	-0.138	0.251
**Random_6**	-0.036	0.143	-0.135	0.217
**Random_7**	-0.048	0.143	-0.111	0.214
**Random_8**	0.011	0.186	-0.087	0.175
**Random_9**	-0.022	0.134	-0.091	0.226
**Random_10**	-0.024	0.126	-0.174	0.187

References

(1) Klebe, G.; Abraham, U.; Mietzner, T. Molecular similarity indices in a comparative analysis (CoMSIA) of drug molecules to correlate and predict their biological activity. *J. Med. Chem.* **1994**, *37*, 4130-4146.

(2) Lézé, M. P.; Le Borgne, M.; Pinson, P.; Palusczak, A.; Duflos, M.; Le Baut, G.; Hartmann, R. W. Synthesis and biological evaluation of 5-[(aryl)(1H-imidazol-1-yl) methyl]-1H-indoles: potent and selective aromatase inhibitors. *Bioorg. Med. Chem. Lett.* **2006**, *16*, 1134-1137.

(3) Lézé, M. P.; Palusczak, A.; Hartmann, R. W.; Le Borgne, M. Synthesis of 6-or 4-functionalized indoles via a reductive cyclization approach and evaluation as aromatase inhibitors. *Bioorg. Med. Chem. Lett*. **2008**, *18*, 4713-4715.

(4) Wang, R.; Shi, H. F.; Zhao, J. F.; He, Y. P.; Zhang, H. B.; Liu, J. P. Design, synthesis and aromatase inhibitory activities of novel indole-imidazole derivatives. *Bioorg. Med. Chem. Lett.* **2013**, *23*, 1760-1762.

(5) Pingaew, R.; Mandi, P.; Prachayasittikul, V.; Prachayasittikul, S.; Ruchirawat, S.; Prachayasittikul, V. Synthesis, molecular docking, and QSAR study of sulfonamide-based indoles as aromatase inhibitors. *Eur. J. Med. Chem.* **2018**, *143*, 1604-1615.

(6) Fantacuzzi, M.; De Filippis, B.; Gallorini, M.; Ammazzalorso, A.; Giampietro, L.; Maccallini, C., Aturki, Z.; Donati, E.; Ibrahim, R.S.; Shawky, E.; Cataldi, A.; Amoroso, R. Synthesis, biological evaluation, and docking study of indole aryl sulfonamides as aromatase inhibitors. *Eur. J. Med. Chem.* **2020**, *185*, 111815.

(7) Lézé, M. P.; Borgne, M. L.; Marchand, P.; Loquet, D.; Kogler, M.; Baut, G. L.; Paluscazk, A.; Hartmann, R.W. 2-and 3-[(aryl)(azolyl) methyl] indoles as potential non-steroidal aromatase inhibitors. *J. Enzyme Inhib. Med. Chem.* **2004**, *19*, 549-557.

(8) Acar Çevik, U.; Sağlık, B. N.; Osmaniye, D.; Levent, S.; Kaya Çavuşoğlu, B.; Karaduman, A. B.; Ozkay, Y.; Kaplancıklı, Z.A. Synthesis and docking study of benzimidazole–triazolothiadiazine hybrids as aromatase inhibitors. *Arch. Pharma.* **2020**, *353*, e2000008.

(9) Saberi, M. R.; Shah, K.; Simons, C. Benzofuran-and furan-2-yl-(phenyl)-3-pyridylmethanols: Synthesis and inhibition of P450 aromatase. *J. Enzyme Inhib. Med. Chem.* **2005**, *20*, 135-141.

(10) Saberi, M. R.; Vinh, T. K.; Yee, S. W.; Griffiths, B. N.; Evans, P. J.; Simons, C. Potent CYP19 (aromatase) 1-[(benzofuran-2-yl)(phenylmethyl) pyridine,-imidazole, and-triazole inhibitors: synthesis and biological evaluation. *J. Med. Chem.* **2006**, *49*, 1016-1022.

(11) Acar Çevik, U.; Kaya Çavuşoğlu, B.; Sağlık, B. N.; Osmaniye, D.; Levent, S.; Ilgın, S.; Ozkay, Yusuf.; Kaplancıklı, Z. A. Synthesis, docking studies and biological activity of new benzimidazole-triazolothiadiazine derivatives as aromatase inhibitor. *Molecules.* **2020**, *25*, 1642.

(12) Ozcan-Sezer, S.; Ince, E.; Akdemir, A.; Ceylan, Ö. Ö.; Suzen, S.; Gurer-Orhan, H. Aromatase inhibition by 2-methyl indole hydrazone derivatives evaluated via molecular docking and in vitro activity studies. *Xenobiotica.* **2019**, *49*, 549-556.

(13) Borgne, M. L.; Marchand, P.; Nourrisson, M. R.; Loquet, D.; Palzer, M.; Le Baut, G.; Hartmann, R. W. Synthesis and biological evaluation of 3-(azolylmethyl)-1 H-indoles and 3-(α-azolylbenzyl)-1 H-indoles as selective aromatase inhibitors. *J. Enzyme Inhib. Med. Chem.* **2007**, *22*, 667-676.

(14) Clark, M.; Cramer III, R. D.; Van Opdenbosch, N. Validation of the general purpose tripos 5.2 force field. *J. Comput. Chem.* **1989**, *10*, 982-1012.

(15) Golbraikh, A.; Tropsha, A. Beware of q2!. *J. Mol. Graph. Model.* **2002**, *20*, 269-276.

(16) Tropsha, A. Best practices for QSAR model development, validation, and exploitation. *Mol. Inform.* **2010**, *29*, 476-488.

(17) Roy, K.; Chakraborty, P.; Mitra, I.; Ojha, P. K.; Kar, S.; Das, R. N. Some case studies on application of "rm2" metrics for judging quality of quantitative structure–activity

relationship predictions: emphasis on scaling of response data. *J. Comput. Chem.* **2013**, *34*, 1071-1082.
(18) Roy, K.; Kar, S.; Ambure, P. On a simple approach for determining applicability domain of QSAR models. *Chemom. Intell. Lab. Syst.* **2015**, *145*, 22-29.
